# HSP90 inhibition potentiates oxidant-based antimelanoma action of novel thioquercetin derivatives by compromising AhR/CYP1A1 pathway

**DOI:** 10.1007/s10495-026-02311-4

**Published:** 2026-03-28

**Authors:** Wojciech Witkowski, Julia Słaby, Maciej Wnuk, Paulina Stec, Piotr Piotrowski, Michał Żebrowski, Martyna Cybularczyk-Cecotka, Anna Deręgowska, Nadezhda Romanchikova, Pawel Zayakin, Aija Linē, María Moros, Grzegorz Litwinienko, Anna Lewińska

**Affiliations:** 1 Faculty of Chemistry, University of Warsaw, Pasteura 1, 02-093 Warsaw, Poland; 2 Doctoral School, University of Rzeszow, Pigonia 1, 35-310 Rzeszow, Poland; 3 Faculty of Biotechnology, Collegium Medicum, University of Rzeszow, Pigonia 1, 35-310 Rzeszow, Poland; 4 Cancer Biomarker Group, Latvian Biomedical Research and Study Centre, Ratsupites 1, Riga, LV-1067 Latvia; 5 Faculty of Medicine and Life Sciences, University of Latvia, Jelgavas 1, Riga, LV- 1004 Latvia; 6 Instituto de Nanociencia y Materiales de Aragón, INMA (CSIC-Universidad de Zaragoza), C/Pedro Cerbuna 12, 50009 Zaragoza, Spain; 7 Centro de Investigación Biomédica en Red de Bioingeniería, Biomateriales y Nanomedicina (CIBER-BBN), 28029 Madrid, Spain

**Keywords:** Melanoma, Thioquercetin, Oxidative stress, HSP90 inhibition, Cytochrome P450

## Abstract

Quercetin, a plant-derived dietary flavonoid, has multifunctional biological activities, including anticancer action; however, its applications may be restricted due to limited bioavailability. Thus, novel synthetic quercetin derivatives (QDs) with improved properties and/or drug combinations should be designed and tested. In the present study, anticancer activity of fourteen newly synthesized QDs was investigated using four cellular models of melanoma, namely A375, MM370, G-361, and SH-4 cells. Thioquercetins (thioQ, thioQ(OAc)_4_, and thioQ(OAc)_5_), when used at low micromolar range, induced apoptotic cell death in melanoma cells compared to normal cells. Thioquercetins also reduced the population of spheroid-forming cells and suppressed the growth of A375 cells in 3D spheroid models. Thioquercetin-mediated antimelanoma action was potentiated upon heat shock protein 90 (HSP90) inhibition. Co-treatment with the HSP90 inhibitor 17-DMAG and thioquercetins augmented oxidative stress (increased superoxide production, decreased levels of antioxidant proteins SOD1, and PRDX1-2), and impaired the aryl hydrocarbon receptor (AhR)/cytochrome P450 1A1 (CYP1A1) signaling pathway-based detoxification of thioquercetins by the inhibition of AhR translocation to the nucleus and AhR-mediated stimulation of *CYP1A1* expression leading to enhanced cytotoxic effects against melanoma cells. The senolytic activity of thioQ(OAc)_4_ with four acetylated hydroxy groups against cisplatin-induced senescent melanoma cells was also revealed in selected experimental settings. We suggest that the use of novel thioquercetin-based derivatives along with HSP90 inhibitors should be further validated in vivo and considered for the design of more effective antimelanoma strategies in the future.

**Graphical abstract:**

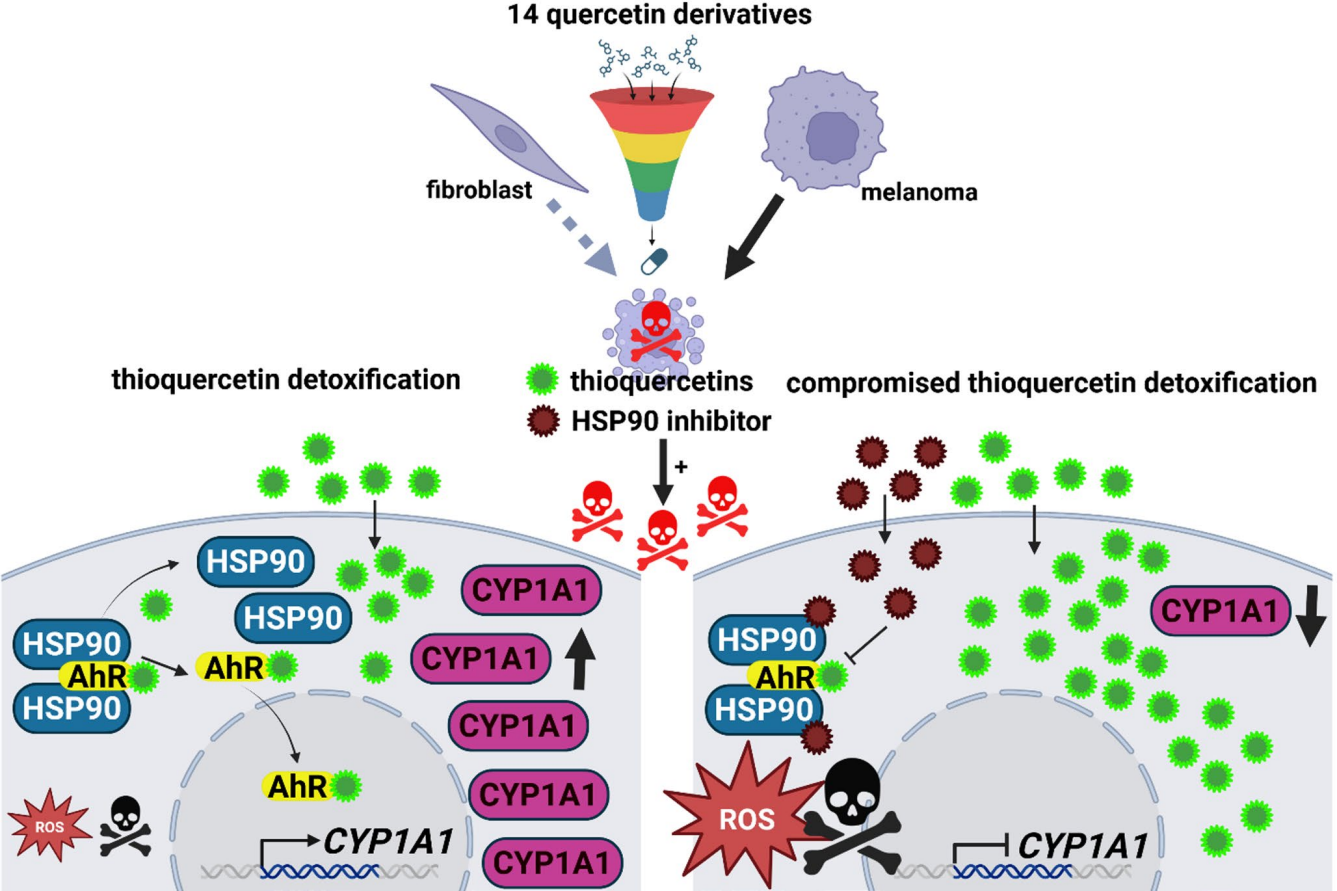

**Supplementary Information:**

The online version contains supplementary material available at 10.1007/s10495-026-02311-4.

## Introduction

A melanocyte-derived melanoma is the third commonest skin cancer characterized by its high invasiveness, metastatic activity and drug resistance, and is also associated with poor prognosis, if advanced [1–4]. Despite the development of novel immunotherapies involving immune checkpoint inhibitors and BRAF and MEK kinase inhibitor-based targeted therapies, metastatic melanoma, with diverse genetic profiles and molecular traits, is still a very hard-to-treat cancer with limited drug responsiveness and therapeutic success [1–4]. Among unique features of melanoma cells are the tumorigenesis-related expression and activity of a key oxidative enzyme in the pigmentation pathway, namely a tyrosinase that metabolizes polyphenolic compounds, which can be a target of polyphenol-based therapies [5]. For example, quercetin (3, 3′, 4′, 5, 7-pentahydroxyflavone), a natural yellow-colored flavonol from the flavonoid group of polyphenols, can be considered as a substrate of tyrosinase that can yield reactive o-quinone compounds and subsequent glutathionyl adducts, resulting in glutathione depletion-mediated oxidative stress and related cytotoxicity in melanoma cells [5, 6]. Quercetin-induced oxidative stress can be also utilized to stimulate the major regulator of antioxidant cytoprotective response, namely nuclear factor (erythroid-derived 2)-like 2 (NRF2) transcriptional activity, NRF2-induced expression of NAD(P)H dehydrogenase [quinone] 1 (NQO1), NQO1-based p53 stabilization, and p53-mediated cell death in cancer cells [5]. Taking into account that the majority of melanomas (up to 84%) are characterized by wild-type tumor suppressor protein p53 [7], quercetin-induced increase in the levels of p53 can indeed trigger apoptotic cell death signals also in melanoma cells.

The anticancer potential of quercetin has been already documented against numerous types of cancer cells in vitro and cell line- or patient-derived xenograft animal models in vivo, including melanoma [8–10]. A broad-spectrum anticancer effects of quercetin are achieved by its ability to modulate the activity of a plethora of cell proliferation, survival and death regulating signaling pathways such as phosphatidylinositol 3-kinase (PI3K)/Akt kinase (Akt), nuclear factor kappa B (NFκB), Ras/Raf/MEK/ERK (mitogen-activated protein kinase, MAPK), Notch, and wingless-type (Wnt)/β-catenin pathways leading to the induction of regulated modes of cell death such as apoptosis and the inhibition of cell growth and division, and cell migration [8–10]. Four aspects of quercetin-mediated antimelanoma strategy have been proposed, namely the stimulation of pro-oxidative activity of quercetin and related redox-active signal transduction cascades, quercetin-induced changes in gene transcription and epigenetic markers, the improvements of quercetin delivery systems, and the effectiveness of combination therapy using quercetin as an adjuvant to conventional melanoma treatment [5]. Despite its potential, therapeutic use of quercetin may have several limitations due to its hydrophobicity, instability in the gastrointestinal tract, rapid metabolism, low bioavailability, and limited target selectivity [9]. Indeed, pro-oxidant and related anticancer effects of quercetin, such as the inhibition of the activity of cytoprotective heat shock proteins (HSPs) affecting the action of key survival kinases of PI3K/Akt and MAPK pathways, are usually observed at the concentrations between 40 and 100 µM in cellular in vitro systems that are hard to be achieved in vivo [11]. Thus, to overcome these restrictions, biocompatible and targeted nano-based quercetin delivery systems such as poly (lactic-co-glycolic acid) (PLGA), chitosan, silk fibroin, metal oxide nanoparticles, and liposomes have been proposed [9, 12, 13]. Furthermore, chemical modifications to produce novel quercetin derivatives (QDs) with improved anticancer properties and/or new drug combinations, involving quercetin or quercetin derivatives as one of the components, are needed to be designed and validated.

In the present study, we have synthesized fourteen novel QDs (i.e., methoxy, aza, acetyl, and thiocarbonyl quercetin analogues and derivatives) and analyzed their antimelanoma effects using four cellular models of melanoma, namely A375, MM370, G-361, and SH-4 cells. Improved antimelanoma efficacy of three thioquercetins: thioQ, thioQ(OAc)_4_, and thioQ(OAc)_5_ was revealed compared to unmodified quercetin, when used at low micromolar concentrations of 5 and 10 µM, that was further potentiated by the co-treatment with a HSP90 inhibitor 17-DMAG (17-desmethoxy-17-N, N-dimethylaminoethylamino-geldanamycin). Thioquercetins promoted oxidative stress and activated the aryl hydrocarbon receptor (AhR)/cytochrome P450 1A1 (CYP1A1)-based detoxification, and HSP90 inhibition abolished CYP1A1-mediated adaptive response enhancing thioquercetin-induced oxidative stress and related cytotoxicity in melanoma cells. Thus, we propose that the use of thioquercetins, when combined with HSP90 inhibition, can facilitate the elimination of melanoma cells in vitro. Furthermore, in selected experimental settings, thioQ(OAc)_4_ can be considered as a novel senolytic agent against drug resistant non-proliferating melanoma cells, namely cisplatin-induced senescent melanoma cells.

## Materials and methods

### Synthesis and characterization of quercetin derivatives (QDs)

**Fig. 1:**
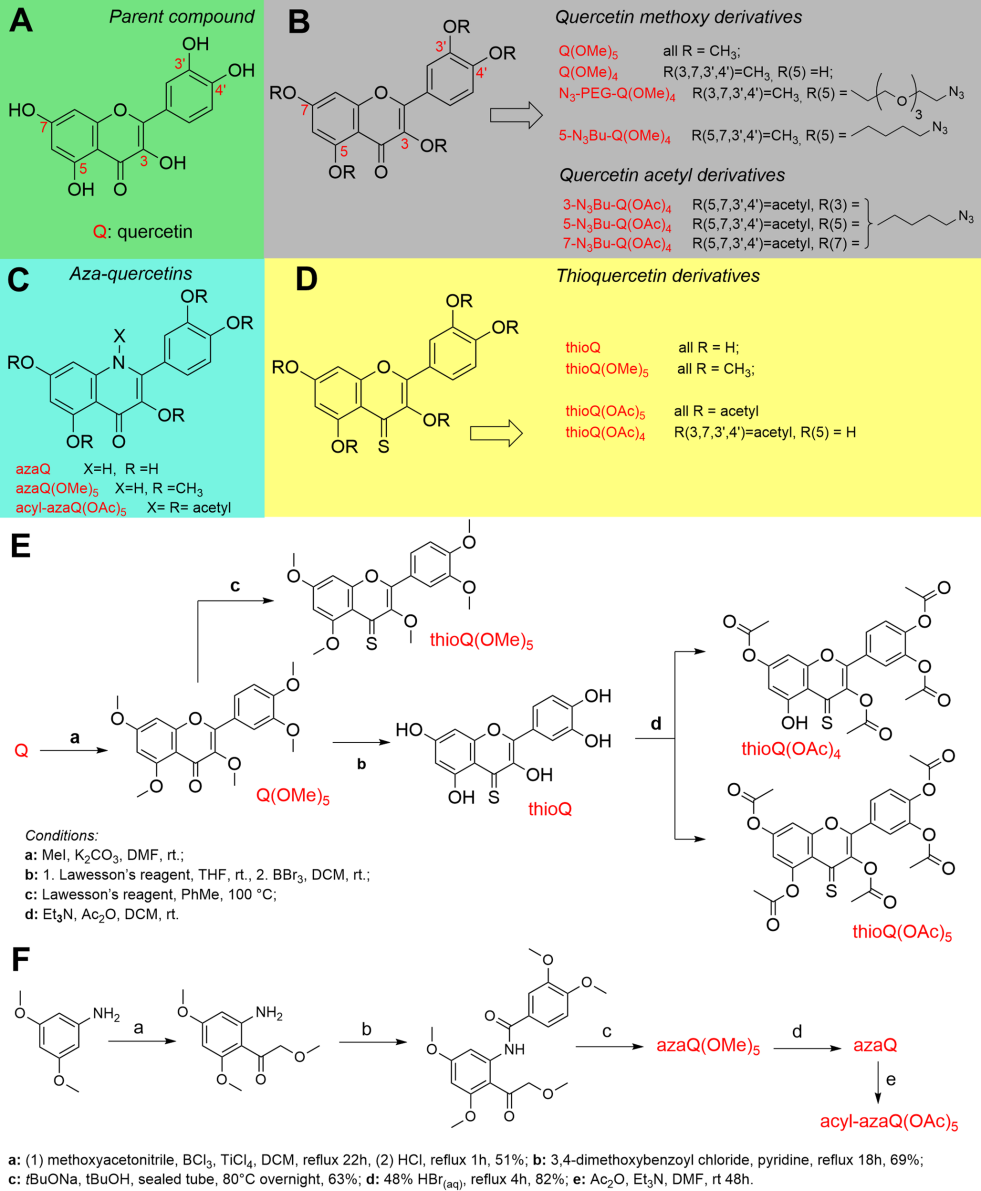
Quercetin (Q) as the parent compound, showing the positions of hydroxy groups (**A**); structures and corresponding abbreviations of methylated and acylated quercetin derivatives (**B**); aza-quercetin derivatives (azaQ, **C**); and derivatives of the thiocarbonyl analogue of quercetin (thioQ, **D**). The strategy for transforming Q into four thiocarbonyl analogues (**E**), and the de novo synthesis of aza-Q derivatives (**F**) are also shown. A complete description of the syntheses of all compounds from panels **B**–**D** is provided in the Supporting Information

The synthesis of the fourteen compounds presented in Fig. 1B–D was carried out through multistep synthetic procedures. While Fig. 1 presents only the synthetic schemes for two types of derivatives, thioQ and azaQ analogues, detailed experimental procedures, reaction conditions, stepwise synthetic schemes, and yields of both intermediate and final products for all fourteen compounds are provided in the Supporting Information (SI). Only a general summary of the methodology is presented here.

All reagents and solvents were obtained from commercial suppliers and used without further purification. Except for Q(OMe)_5_, all quercetin derivatives (Fig. 1B) were obtained via multistep syntheses. Partial methylation of quercetin with standard reagents (CH_3_I, K_2_CO_3_) afforded Q(OMe)_4_, whose unmodified 5-hydroxy group was used to introduce azide-containing linkers through reaction with either α,ω-dibromobutane or α,ω-dibromoPEG, followed by substitution of bromine with an azide group to yield 5-N_3_Bu-Q(OMe)_4_ and N_3_-PEG-Q(OMe)_4_, respectively [14]. Acylated analogues bearing azide moieties at positions 3, 5, and 7 (N_3_Bu-Q(OAc)_4_) were synthesized through regioselective protection, chloroalkylation, acylation, and halogen-azide substitution steps (Scheme S2). The synthetic pathway leading to the 3-substituted derivative was developed based on the high reactivity of position 3 toward alkylation once the catechol moiety had been appropriately protected. The 5-substituted derivative was obtained following a literature-reported method employing benzyl chloride as a protecting agent, which enables selective deprotection of the 5-hydroxy group for further modification. In contrast, the synthesis of the 7-substituted analogue exploited acetyl migration under the alkylation conditions applied to 3′,4′-O-diphenylmethane-3,7-diacetylquercetin. Despite its greater complexity, this route proved to be the only effective approach to the desired compound. The resulting O-alkyl linkages exhibited high stability under subsequent reaction conditions, whereas the acetyl protecting groups were prone to hydrolysis. Consequently, a second acetylation step was required in the synthesis of the 7-substituted derivative to ensure complete protection of OH groups throughout the process (Preparation of quercetin acetyl derivatives section in SI). Thioquercetin (thioQ) and its derivatives (Fig. 1D) were obtained by full methylation of quercetin (Q), followed by conversion of the carbonyl group to a thiocarbonyl using Lawesson’s reagent [15] (Fig. 1E and Preparation of thioquercetin (thioQ)and its derivatives section in SI)). Subsequent deprotection and acylation afforded thioQ and its O-acylated derivatives (Scheme S3). Aza-quercetin (azaQ) was synthesized de novo from 3,5-dimethoxyaniline and 3,4-dimethoxybenzoyl chloride, with intramolecular cyclization forming the central heterocyclic ring (Fig. 1F) [16, 17]. Demethylation of azaQ(OMe)_5_ yielded azaQ, which upon acylation produced acyl-azaQ(OAc)_5_ derivatives.

All final products were purified by column chromatography using silica gel 60 M (40–63 μm, 230–440 mesh). The structures of the target molecules as well as the intermediates were unambiguously confirmed by NMR analysis (with comparison to compounds described in the literature, if possible). NMR spectra were recorded at room temperature using Bruker 300 MHz (^1^H NMR) or 75 MHz (^13^C NMR) spectrometer. The purity analysis is provided in SI. The lowest recorded purity was 97.2% for thioQ, 99.0% for thioQ(OAc)_4_, and 98.7% for thioQ(OAc)_5_.

### Cell lines, culture conditions, and treatments

Four melanoma cell lines were used, namely A375 (malignant melanoma/epithelial, CRL-1619™, ATCC, Manassas, VA, USA), MM370 (malignant melanoma/epithelial, 10092316, CBA-1348, distributed by ECACC on behalf of CellBank Australia (CBA), Children’s Medical Research Institute, Westmead, Australia), G-361 (malignant melanoma/epithelial, 88030401, ECACC, Public Health England, Porton Down, Salisbury, UK), and SH-4 (melanoma/mixture of spindle-shaped and epithelial-like cells, CRL-7724™, ATCC, Manassas, VA, USA). As control cells, proliferatively active human foreskin fibroblasts (BJ cells at population doubling levels = 35 [18], CRL-2522™, ATCC, Manassas, VA, USA) were exploited. For 2D cell cultures, A375, G-361, SH-4, and BJ cells were cultured in Dulbecco’s modified Eagle medium (DMEM), whereas MM370 cells were cultured in Roswell Park Memorial Institute (RPMI) 1640 medium. All culture media were supplemented with 10% fetal bovine serum (FBS) and antibiotic/antimycotic mix (100 U/ml penicillin, 0.1 mg/ml streptomycin, and 0.25 µg/ml amphotericin B) (Corning, Tewksbury, MA, USA). Cells were maintained at 37 °C in a 5% CO_2_ incubator. For passaging, a solution of 0.25% trypsin/2.21 mM EDTA (Corning, Tewksbury, MA, USA) was used and for the majority of experiments, cells were seeded at a density of 10^4^ cells per cm^2^ of culture flask or plate. Cells were treated with unmodified quercetin (Q, Q0125, Merck KGaA, Darmstadt, Germany) and newly synthesized QDs at the concentrations of 5 and 10 µM for 24 h. The concentrations were selected based on MTT results. For selected experimental settings, melanoma cells were co-treated with 5 µM thioquercetins and 100 nM 17-DMAG (17-desmethoxy-17-N, N-dimethylaminoethylamino-geldanamycin, a HSP90 inhibitor, AA43412, Biosynth, Staad, Switzerland) for 24 h. For AhR nuclear translocation analysis, melanoma cells were also treated for 6 h.

### Multicellular spheroid-formation assay

To generate multicellular 3D spheroids, A375 single cell suspensions were seeded into ultra-low attachment U-bottom BIOFLOAT 96-well plate (faCellitate, Mannheim, Germany) at a density of 8 × 10^3^ viable cells per well. Cells were cultured in 200 µl of DMEM/F12 GlutaMax medium (Thermo Fisher Scientific, Waltham, MA, USA) supplemented with 1 x B27 (Thermo Fisher Scientific, Waltham, MA, USA), 2 mM L-glutamine, 1 x antibiotic-antimycotic mix (Thermo Fisher Scientific, Waltham, MA, USA), 20 ng/ml hEGF (R&D Systems, Minneapolis, MN, USA), 10 ng/ml basic hFGF (Santa Cruz Biotechnology, Dallas, TX, USA), 10 µg/ml insulin, and 0.5 µg/ml hydrocortisone (Merck KGaA, Darmstadt, Germany). Quercetin and selected QDs, namely thioQ, thioQ(OAc)_4_, and thioQ(OAc)_5_ were dissolved in DMSO and added to the culture medium at a final concentration of 5 µM. Control cells (CTR) were treated with an equivalent volume of DMSO. Spheroids were cultured for 120 h. The drug-containing medium was changed every 48 h. Live/dead cell staining was performed using 1 µg/ml Hoechst 33342 (Thermo Fisher Scientific, Waltham, MA, USA) and 5 µg/ml propidium iodide (Merck KGaA, Darmstadt, Germany) at 37 °C for 15 min. Images were captured using the BioTek Cytation 5 Cell Imaging Multimode Reader (Agilent Technologies, Inc., Santa Clara, CA, USA). Spheroid area was quantified by ImageJ software (https://imagej.nih.gov/ij/) using a custom macro developed with the BioVoxxel Toolbox and calculated as a percentage of CTR. All experiments were performed in 4–5 replicates and repeated independently on two separate days.

### Clonal spheroid-formation assay

To generate clonal spheroids, A375 single-cell suspensions were embedded in a semi-solid Matrigel matrix. Matrigel (Corning, Tewksbury, MA, USA) was thawed on ice overnight prior to use. Cells were seeded at a density of 250 cells per well in a 24-well plate (Sarstedt, Nümbrecht, Germany). For each well, a mixture of 100 µl of cold cell suspension and 100 µl of cold Matrigel was prepared in serum-free growth medium (DMEM/F12 GlutaMax; Thermo Fisher Scientific, Waltham, MA, USA) supplemented with 1× B27, 2 mM L-glutamine, 1× antibiotic–antimycotic mix, 20 ng/ml hEGF, 10 ng/ml basic hFGF, 10 µg/ml insulin, and 0.5 µg/ml hydrocortisone (all from the suppliers listed in a subsection *Multicellular spheroid-formation assay*). Q, thioQ, thioQ(OAc)_4_, and thioQ(OAc)_5_ were added to the culture medium at a final concentration of 5 µM. The Matrigel-cell mixture was carefully pipetted to the bottom of each well, avoiding bubble formation, and plates were incubated at 37 °C with 5% CO_2_ in a humidified incubator for 60 min to allow gel solidification. Following polymerization, 0.5 ml of warm culture medium containing the corresponding compounds was gently added to each well. The medium was replaced every 2–3 days. On day 8, spheroids larger than 100 μm were counted under an inverted microscope. Spheroid number is calculated as a percentage of CTR. All experiments were performed in triplicate and independently repeated on two separate days.

### Apoptosis

For screening of cytotoxic potential of quercetin derivatives, two markers of apoptotic cells death were considered, namely phosphatidylserine externalization and caspase 3/7 activity. Upon treatment, cells were subjected to dedicated stainings (Muse^®^ Annexin V & Dead Cell Kit and Muse^®^ Caspase-3/7 Kit, respectively) and flow cytometry-based analysis (Muse^®^ Cell Analyzer, Cytek Biosciences, Amsterdam, The Netherlands). Furthermore, both stainings were based on dual staining with a necrotic death marker, 7-aminoactinomycin D (7-AAD) to distinguish between early and late apoptosis events as well as solely necrotic morphotype of cell death. The same approach was used to analyze the cytotoxic effects of thioquercetins upon co-treatment with the HSP90 inhibitor 17-DMAG.

### Oxidative stress

Upon treatment, thioquercetin-mediated oxidative stress, as judged by changes in the levels of intracellular superoxide, was studied using a fluorescent dye dihydroethidium and flow cytometry (Muse^®^ Oxidative Stress Kit and Muse^®^ Cell Analyzer, Cytek Biosciences, Amsterdam, The Netherlands). Briefly, upon dedicated staining, superoxide-positive and superoxide-negative cell subpopulations were revealed (%). The same approach was used to analyze thioquercetin-mediated oxidative stress upon co-treatment with the HSP90 inhibitor 17-DMAG.

### Imaging cytometry

Upon treatment or co-treatment, melanoma cells were fixed and subjected to immunofluorescence protocol as previously described [19]. The following primary and secondary antibodies were applied, namely anti-NRF2 (1:200, PA5-27882), anti-FOXO3a (1:200, MA5-14932), anti-SOD1 (1:200, PA5-23245), anti-SOD2 (1:500, MA1-106), anti-PRDX1 (1:250, PA3-750), anti-PRDX2 (1:250, 10545-2-AP), anti-CYP1A1 (1:50, PA5-15213), anti-AhR (1:50, 83200), anti-HSP90 (1:100, MA1-10373), goat anti-rabbit IgG (H + L) cross-adsorbed secondary antibody conjugated with Texas Red (1:1000, T-2767), goat anti-mouse IgG (H + L) cross-adsorbed secondary antibody conjugated with FITC (1:1000, F-2761), and F(ab’)2-goat anti-rabbit IgG (H + L) secondary antibody conjugated with PE-Cyanine5.5 (1:1000, L43018) (Thermo Fisher Scientific, Waltham, MA, USA, Cell Signaling Technology, Danvers, MA, USA). Nuclei were stained using Hoechst 33342 staining. Fluorescence signals were acquired and analyzed using confocal imaging system IN Cell Analyzer 6500 HS and IN Carta software (Cytiva, Marlborough, MA, USA). Total protein levels or nuclear pools of selected proteins are presented as relative fluorescence units (RFU).

### Real-time PCR

Upon treatment or co-treatment, total RNA was isolated using GenElute™ Mammalian Total RNA Miniprep Kit (Merck KGaA, Darmstadt, Germany) and cDNA was then synthesized using Transcriptor First Strand cDNA Synthesis Kit (Roche, Basel, Switzerland). For the analysis of mRNA levels of *CYP1A1*, Applied Biosystems StepOnePlus™ Real-Time PCR System, TaqMan™ Fast Advanced Master Mix (4444963) and dedicated TaqMan™ Gene Expression Assay (FAM) (4331182, Thermo Fisher Scientific, Waltham, MA, USA), namely *CYP1A1* Hs01054796_g1 were used according to the manufacturer’s instructions. *GAPDH* Hs03929097_g1 was considered as a reference gene.

### Interaction analysis between 17-DMAG and/or thioquercetins and HSP90

The interaction between a HSP90 inhibitor 17-DMAG and/or thioquercetins and HSP90 was investigated using a cell-free in vitro system as previously described [20]. Briefly, 50 ng of recombinant human HSP90 (SRP4858) (Merck KGaA, Darmstadt, Germany) was incubated with 100 nM 17-DMAG and/or 5 µM thioquercetins or appropriate aliquots of DMSO (solvent control) in 20 mM Tris-HCl buffer (pH 8) containing 150 mM NaCl and 1 mM DTT at 37 °C overnight. Samples in a volume of 2 µl were then pipetted into an Amersham Protran nitrocellulose membrane (10600003, Merck KGaA, Darmstadt, Germany) and incubated with a primary antibody anti-HSP90 (1:1000, MA1-10372, Thermo Fisher Scientific, Waltham, MA, USA) at 4 °C overnight. The membranes were then incubated with a secondary antibody anti-mouse IgG (1:3000, 7076) (Cell Signaling Technology, Danvers, MA, USA) at room temperature for 1 h. Next, the membranes were subjected to the incubation with Clarity Western ECL substrate chemiluminescent detection reagent (Bio-Rad, Hercules, CA, USA) for 5 min and proceeded to image acquisition (ChemiDoc imaging system, Bio-Rad, Hercules, CA, USA). Densitometry-based dot analysis was performed using ImageJ software (https://imagej.nih.gov/ij/). Chemiluminescence data are presented as relative density units normalized to control conditions (CTR). Control conditions were assumed as appropriate aliquots of 20 mM Tris-HCl buffer (pH 8) containing 150 mM NaCl and 1 mM DTT (a buffer system used for interaction analysis) and 50 ng of recombinant human HSP90.

### Activation of chemotherapy-induced senescence

Cisplatin (232120, Merck KGaA, Darmstadt, Germany) treatment was used to promote drug-induced senescence in melanoma cells as previously reported [21]. Briefly, the senescence inducing concentration of cisplatin was optimized for each melanoma cell line, namely 4, 3, 2, and 5 µM for A375, MM370, G-361, and SH-4 cells, respectively [21]. Melanoma cells were treated with cisplatin for 24 h, the drug was then removed, and cells were cultured for additional 7 days to develop drug-induced senescent phenotype that was verified using several biomarkers of senescence as previously reported [21].

### Analysis of senolytic activity of thioquercetins and oxidative stress in senescent melanoma cells

Upon induction of cisplatin-stimulated senescence program, senescent melanoma cells were subjected to the treatment with thioquercetins or co-treatment with the HSP90 inhibitor, 17-DMAG for 24 h, and treatment or co-treatment-mediated apoptosis induction (senolytic activity) was investigated using Annexin V staining and flow cytometry as described in the subsection *Apoptosis*. Furthermore, thioquercetin and co-treatment-mediated induction of oxidative stress in cisplatin-induced senescent melanoma cells was studied as the analysis of the levels of superoxide using dihydroethidium fluorogenic probe as described in the subsection *Oxidative stress*.

### Statistical analysis

The presented results are calculated as arithmetic mean ± standard deviation or alternatively box and whisker plots with median, lowest, and highest values were applied. Three independent biological replicates were considered to study changes in analyzed parameters. Statistical significance of differences between treated samples and untreated control was assessed using one-way analysis of variance (ANOVA) and Dunnett’s multiple comparison test. Furthermore, differences between thioquercetin treatment and co-treatment with a HSP90 inhibitor 17-DMAG were revealed using one-way analysis of variance (ANOVA) and Tukey’s multiple comparison test. Statistical analysis was performed using GraphPad Prism 8 and *p* values of less than 0.05 were assumed as statistically significant.

## Results and discussion

### Synthesis of quercetin derivatives (QDs)

It is widely accepted that chemical modifications of flavones such as methylation, acetylation, and thiocarbonylation can improve their lipophilicity and bioavailability, and promote their reactivity and biological activity, respectively [15, 22]. For example, potentiated broad-spectrum anticancer action of synthetic thioflavones containing chloro and/or bromo groups was reported compared to the effects exerted by natural flavone chrysin [15]. Replacement of oxygen with sulfur or nitrogen in phenolic scaffolds represents a compelling molecular design strategy for tuning redox properties and expanding biological functionality. Both sulfur and nitrogen introduce distinct electronic and stereochemical features that can significantly modify the mechanisms underlying radical scavenging and redox cycling. Substituting oxygen with these heteroatoms affects parameters such as bond dissociation enthalpy (BDE), ionization potential, and proton affinity, thereby modulating the molecule’s reactivity toward reactive oxygen species (ROS) and its overall antioxidant/prooxidant efficiency [23–25]. Acetylated quercetin derivatives were employed as prodrug-like forms to enhance lipophilicity and cellular bioavailability, with the expectation that intracellular esterases will hydrolyze the acetyl groups and regenerate the active parent flavonoid after cellular uptake. In contrast, the corresponding methylated derivatives were included as a control group because O-methyl substituents are resistant to esterase-mediated hydrolysis, allowing discrimination between biological effects arising from enzymatic deacetylation and those intrinsic to permanent structural modification of the quercetin scaffold [26, 27]. Thus, in the present study, fourteen quercetin derivatives were synthesized (e.g., methoxy, acetyl, aza, and thiocarbonyl QDs) to analyze their antimelanoma activity in comparison with the corresponding activity induced by unmodified quercetin with limited bioavailability.

Except for Q(OMe)_5_, the quercetin derivatives shown in Fig. 1B were obtained through multistep syntheses. The methylated derivatives were prepared by methylation with iodomethane, and the choice of solvent and appropriate stoichiometric ratios enabled partial methylation, leaving the 5-hydroxy group of Q(OMe)_4_ unmodified. This free 5-OH group was subsequently utilized for the attachment of an N_3_-containing substituent via either an *n*-butyl linker, yielding 5-N_3_Bu-Q(OMe)_4_, or a polyether linker, as in N_3_-PEG-Q(OMe)_4_ (Fig. 1B and Scheme S1). The synthesis of acylated quercetin derivatives bearing a butyl azide moiety at three distinct positions, namely 3-, 5-, and 7-N_3_Bu-Q(OAc)_4_, was designed and performed based on prior experience with chemical modifications of quercetin and fisetin [20, 28]. Regioselective derivatives were obtained via six- to seven-step syntheses starting from unsubstituted Q. The synthetic route involved sequential protection of selected hydroxy groups with diphenylmethyl or benzyl protecting groups, chloroalkylation of the remaining free OH, deprotection and acylation, and subsequent substitution of chlorine with iodine, followed by conversion of the iodide to the corresponding azide functionality via the butyl linker (Scheme S2, including yields of particular steps). In the case of sulfur substitution, the lower electronegativity and higher polarizability of sulfur compared with oxygen stabilize radical intermediates and favor media-mechanisms involving hydrogen bonding, acid strengths and metal-binding. These changes frequently translate into improved biological outcomes, including modulation of oxidative stress pathways. Such mechanistic principles have guided the design of sulfur-enriched analogues of natural antioxidants. Thioquercetin (thioQ) and its derivatives (Fig. 1D) were prepared via methylation of all OH groups in quercetin, and transformation of carbonyl into thiocarbonyl upon reaction with Lawesson’s reagent (Fig. 1E and Scheme S3). Deprotection of OH groups resulted in thioQ, subsequently acylated to thioQ(OAc)_4_ or thioQ(OAc)_5_, depending on stoichiometric excess of acylating agent (acetic anhydride and triethyl amine, see SI). Nitrogen substitution offers a complementary approach. Incorporation of amine or amide functionalities can lower redox potentials, facilitate hydrogen bonding, and alter solubility and cellular uptake. Nitrogen-containing analogues are capable of reversible proton-coupled electron transfer and can serve as electron reservoirs in biological environments. To explore these effects, an aza-quercetin (azaQ) series was designed and synthesized. In contrast to thio-analogues, the azaQ scaffold was constructed de novo rather than derived directly from quercetin, using suitably functionalized aniline and benzoyl chloride precursors to assemble the heterocyclic core. Subsequent deprotection and acylation steps afforded the parent azaQ and its acylated derivatives, as detailed in Fig. 1F.

All designed compounds were successfully synthesized in satisfactory yields, taking into account the multistep nature of the synthetic routes, and were isolated with high purity.

### Thioquercetins are active against melanoma cells

**Fig. 2:**
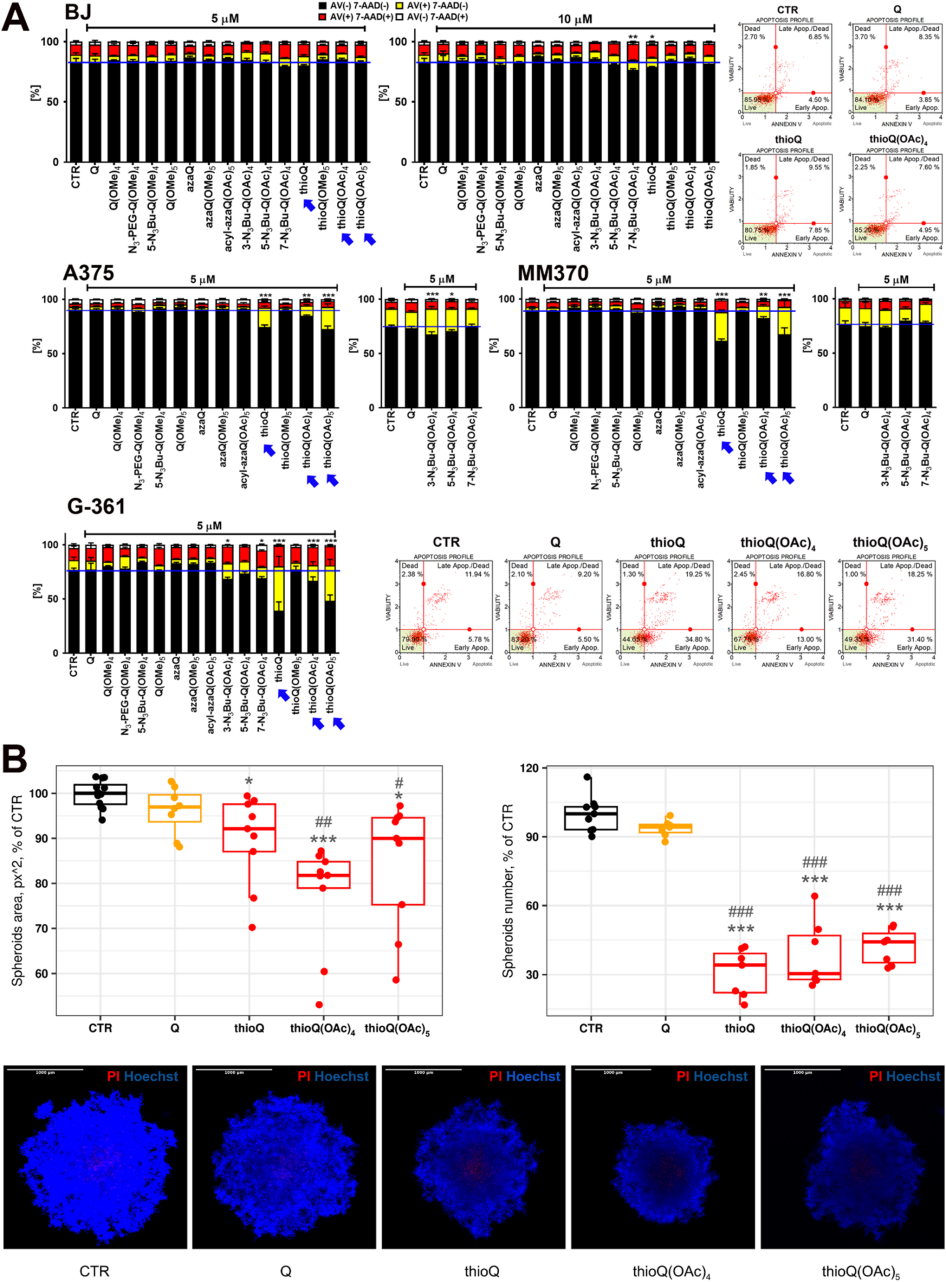
Quercetin derivative-induced apoptotic cell death in four melanoma cell lines, namely A375, MM370, G-361, and SH-4 using 2D cell culture system (**A**) and thioquercetin-mediated effects on A375 cell-based 3D melanoma spheroids (**B**). (**A**) Human normal fibroblasts (BJ cells) served as control cells. Cells were treated with quercetin (Q) and fourteen quercetin derivatives (5 µM for 24 h, BJ cells were also treated with 10 µM quercetin derivatives). Apoptosis and necrosis were evaluated using dual staining based on Annexin V staining and 7-AAD staining, and flow cytometry. Bars indicate SD, *n* = 3, ***p < 0.001, **p < 0.01, *p < 0.05 compared to control (CTR) (ANOVA and Dunnett’s a posteriori test). A blue horizontal line is used to emphasize the action of quercetin derivatives compared to CTR. Representative dot-plots are also shown. Blue arrows indicate the most pronounced action of three thioquercetins, namely thioQ, thioQ(OAc)_4_, and thioQ(OAc)_5_ against melanoma cells and no action against BJ normal cells. These three thioquercetins were selected for further analysis. (**B**) Multicellular spheroid-formation assay (left and bottom) and clonal spheroid-formation assay (right) were considered. (Left panel) Box plots showing the area of multicellular spheroids formed by seeding 8x10^3^ A375 cells in dedicated plates in the presence of quercetin or its derivatives at the concentration of 5 µM. Each dot represents the area of an individual spheroid, measured using the BioTek Cytation 5 Cell Imaging Multimode Reader, and expressed as a percentage relative to untreated controls (CTR). Live/dead staining of multicellular A375-based spheroids was also considered (bottom panel). Images were captured using the BioTek Cytation 5 Cell Imaging Multimode Reader and representative microphotographs are shown. Scale bar, 1x10^3^ μm; PI, propidium iodide (red); Hoechst, Hoechst 33342 (blue). (Right panel) Box plots showing the number of spheroids formed by seeding 250 cells in semi-solid Matrigel in the presence of quercetin or its derivatives at the concentration of 5 µM. Each dot represents the spheroid count per well, expressed as a percentage relative to untreated controls (CTR). Box and whisker plots are shown, *n* = 3, ***p < 0.001, *p < 0.05 compared to control (CTR) (ANOVA and Dunnett’s a posteriori test), ^###^*p* < 0.001, ^##^*p* < 0.01, ^#^*p* < 0.05 compared to the treatment with quercetin (ANOVA and Tukey’s a posteriori test)

The effectiveness of fourteen newly synthesized quercetin derivatives against three melanoma cell lines (A375, MM370, and G-361 classified as malignant melanoma with epithelial morphology) was then initially screened using Annexin V staining to reveal QD-induced apoptotic cell death (Fig. 2A). First, QD-mediated apoptosis was analyzed in human normal fibroblasts considered as control cells. We did focus on low micromolar concentrations of QDs (5 and 10 µM) due to the fact that plant-derived flavonoid quercetin and related compounds are characterized by low bioavailability in biological systems [9]. 5 µM quercetin and 5 µM QDs did not promote cytotoxic effects in normal fibroblasts cells (Fig. 2A). 5 µM quercetin also did not induce apoptosis in three melanoma cell lines (Fig. 2A). In contrast, treatment with 5 µM thioquercetin derivatives resulted in moderate induction of apoptotic cell death in all melanoma cell lines and G-361 cells were the most sensitive to the treatment with thioquercetins, especially with a thioquercetin with unmodified hydroxy groups (thioQ) (Fig. 2A). Upon treatment with 10 µM thioQ and other thioquercetins such as thioQ(OAc)_4_ and thioQ(OAc)_5_ with four and five acetylated hydroxy groups, respectively, apoptosis induction was much more pronounced than after treatment with 5 µM thioquercetin derivatives in melanoma cells (Fig. S46). One exception was thioQ(OMe)_5_, a thioquercetin with five methylated hydroxy groups, as thioQ(OMe)_5_ was not active against three melanoma cell lines, when used at the concentrations of 5 and 10 µM (Fig. 2A and Fig. S46). Thus, thioQ(OMe)_5_ was excluded from further analysis. Other QDs did not induce cytotoxic effects or their cytotoxic effects were less pronounced than thioquercetins, thus thioQ, thioQ(OAc)_4_ and thioQ(OAc)_5_ at the concentration of 5 µM were selected for further analysis (Fig. 2A, denoted with blue arrows).

The rationale for the selection of 5 µM concentration of thioquercetins was also based on the fact that 10 µM concentration of thioQ was slightly cytotoxic against normal BJ cells (Fig. 2A). Thus, the selectivity (i.e., antimelanoma activity) of concentration of 10 µM of thioQ was limited compared to lower one (5 µM) in our experimental settings (Fig. 2A). To compare the antimelanoma action of fourteen QDs at 5 µM in terms of the impact of chemical modification on apoptosis induction, semiquantitative analysis was also performed (four QD-mediated effects were distinguished, namely no effect, minor effect, moderate effect, strong effect) (Table 1).

**Table 1:** Comparative analysis of apoptotic activity of different quercetin derivatives (QDs) and parent compound (unmodified quercetin, Q) in normal human BJ fibroblasts and three melanoma cell lines (A375, MM370, and G-361)

Compound	Cell line			
	Normal	Melanoma		
	BJ	A375	MM370	G-361
Q	0	0	0	0
Q(OMe)_4_	0	0	0	0
N_3_-PEG-Q(OMe)_4_	0	0	0	0
5-N_3_Bu-Q(OMe)_4_	0	0	0	0
Q(OMe)_5_	0	0	0	0
azaQ	0	0	0	0
azaQ(OMe)_5_	0	0	0	0
acyl-azaQ(OAc)_5_	0	0	0	0
3-N_3_Bu-Q(OAc)_4_	0	+	0	+
5-N_3_Bu-Q(OAc)_4_	0	+	0	0
7-N_3_Bu-Q(OAc)_4_	0	0	0	+
thioQ	0	++	++	+++
thioQ(OMe)_5_	0	0	0	0
thioQ(OAc)_4_	0	+	+	+
thioQ(OAc)_5_	0	++	++	+++

Cells were treated with QDs and Q at 5 µM for 24 h and apoptosis was revealed using Annexin V staining. 0, no effect; statistically significant effects are denoted as +++ (strong effect), ++ (moderate effect), + (minor effect)

This can highlight improved effectiveness of thioquercetins against melanoma cells compared to other QDs and parent compound Q (Table 1). However, as we did not analyze the uptake of thioquercetins, one cannot claim that this increased cytotoxicity is based on improved bioavailability of thioquercetins in vitro. More studies are needed to address these issues in the future.

Antimelanoma effects mediated by 5 µM thioquercetins were also validated using A375 cell-based 3D spheroid model and two assays, namely multicellular spheroid-formation and clonal spheroid-formation tests (Fig. 2B). Thioquercetins markedly reduced the number of clonal spheroids as compared to untreated controls, indicating their ability to reduce the number of cells capable to initiate the spheroid formation. They also decreased the area of multicellular spheroids formed by the aggregation and proliferation of cells, thus demonstrating antiproliferative activity, while exhibiting only minimal cytotoxicity as assessed by propidium iodide (PI) staining. Among thioquercetins tested, the most effective in term of reduction in spheroid area and number was thioQ(OAc)_4_ (Fig. 2B).

### Thioquercetin-mediated cytotoxicity may be potentiated upon co-treatment with a HSP90 inhibitor

**Fig. 3:**
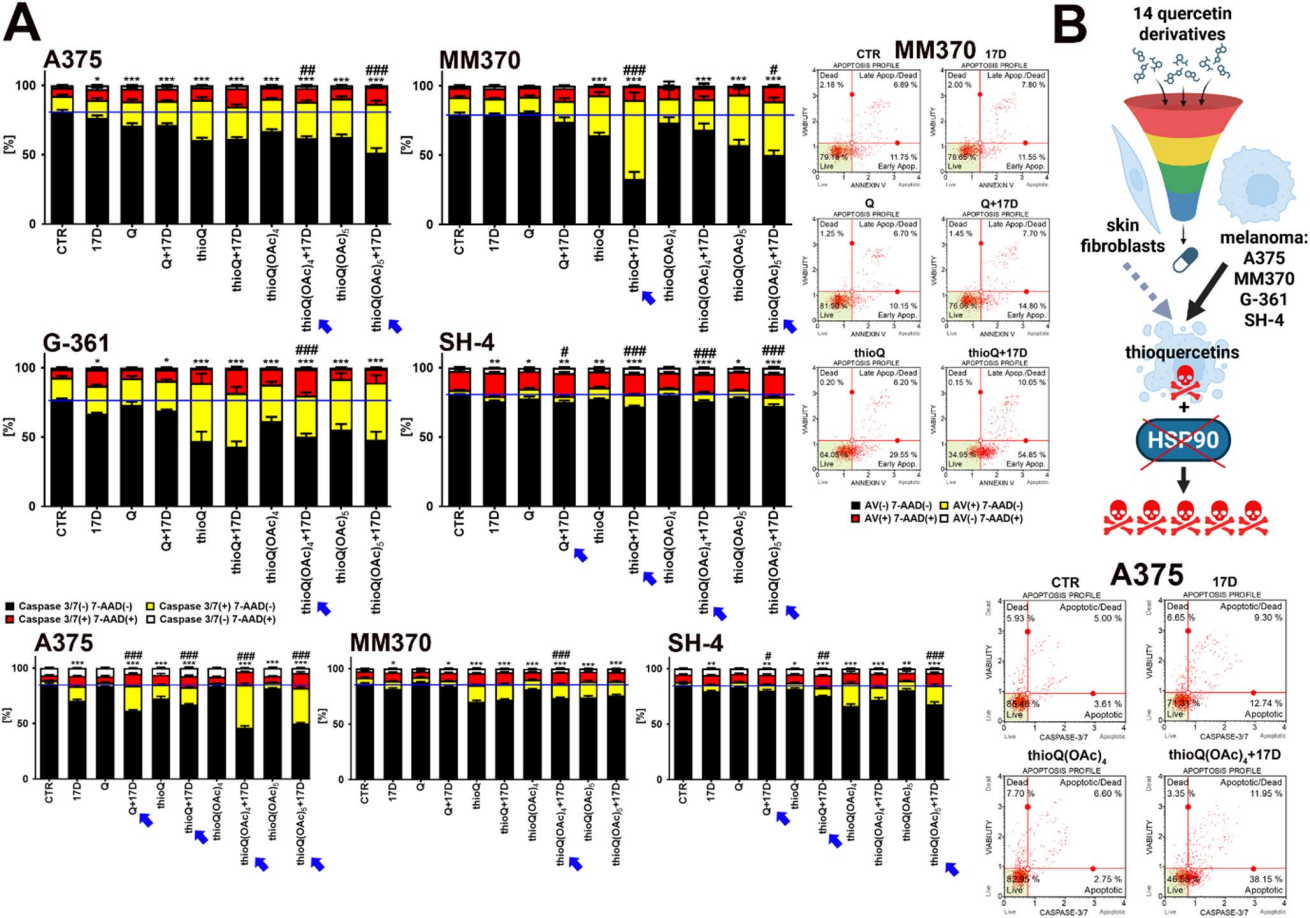
Synergistic action of HSP90 inhibition in thioquercetin derivative-treated melanoma cell lines, namely A375, MM370, G-361, and SH-4. (**A**) Melanoma cells were co-treated with 5 µM quercetin or thioquercetins and 100 nM 17-DMAG, a HSP90 inhibitor, for 24 h. Apoptosis was analyzed using two assays, namely Annexin V staining (top) and caspase 3/7 activity test (bottom), and flow cytometry. Bars indicate SD, *n* = 3, ***p < 0.001, **p < 0.01, *p < 0.05 compared to control (CTR) (ANOVA and Dunnett’s a posteriori test), ^###^*p* < 0.001, ^##^*p* < 0.01, ^#^*p* < 0.05 compared to the treatment with quercetin or corresponding thioquercetin derivative (ANOVA and Tukey’s a posteriori test). A blue horizontal line is used to emphasize the action of quercetin derivatives and HSP90 inhibitor compared to CTR. Representative dot-plots are also shown. Blue arrows indicate potentiated proapoptotic activity upon co-treatment. (**B**) A summarizing scheme showing the outcome of initial apoptosis-based cytotoxicity screening of fourteen quercetin derivatives against melanoma cells, namely the selection of the most effective quercetin derivatives - thioquercetins. The synergistic action of HSP90 inhibition is also denoted. CTR, control conditions; Q, quercetin treatment; 17D, treatment with the HSP90 inhibitor alvespimycin (17-DMAG)

As the cytotoxic effects of 5 µM thioquercetins were moderate in all melanoma cells except for G-361 cells (Fig. 2A), we next asked the question of whether thioquercetin-mediated apoptotic cell death may be potentiated as a result of co-treatment with the HSP90 inhibitor 17-DMAG (Fig. 3). HSP90, an ATP-dependent molecular chaperone involved in protein folding and maturation, is frequently overexpressed in cancer cells and tissues, including melanoma, promoting the activity of numerous HSP90 client proteins, considered as oncoproteins, and related pro-survival signals [29–32]. For example, HSP90 client oncoproteins are key components of melanoma-associated signaling pathways such as BRAF mutants (MAPK pathway), Akt and mTOR (PI3K/Akt pathway), IκB kinase (IKK) (NFκB pathway), β-catenin (Wnt/ β-catenin pathway), and IRE1α (UPR, unfolded protein response) facilitating, among others, melanoma proliferation, survival, invasion, migration, metabolism, self-renewal, protein synthesis and folding, and endoplasmic reticulum (ER) homeostasis [31]. Thus, HSP90 targeting has been considered as an attractive single or complementary antimelanoma strategy affecting the viability of both primary and drug resistant melanoma cells [31, 33]. Several classes of HSP90 inhibitors have been documented, namely N‑terminal domain inhibitors such as geldanamycin and their derivatives tanespimycin (17-AAG), alvespimycin (17-DMAG), and 17-aminogeldanamycin (17-AG), middle domain inhibitors and C-terminal domain inhibitors using different mechanisms to promote HSP90 inhibition-mediated antimelanoma activity [31, 32, 34, 35].

Indeed, co-treatment with 100 nM 17-DMAG also potentiated thioquercetin-mediated apoptosis in melanoma cells in selected experimental settings (Fig. 3A). This effect was most pronounced in MM370 cells co-treated with thioQ and 17-DMAG (Fig. 3A). We have also used another melanoma cell line SH-4 with distinct morphology, namely mixture of spindle-shaped and epithelial-like cells, and found that this less susceptible to thioquercetin stimulation cell line may be slightly sensitized to thioquercetins when co-treated with 17-DMAG (Fig. 3A). To further characterize the apoptotic cell death, we considered another marker of apoptosis, namely the activity of two executioner caspases 3 and 7, which is observed later than phosphatidylserine externalization revealed by Annexin V staining (Fig. 3A). In A375 cells co-treated with thioQ(OAc)_4_ and 17-DMAG, and thioQ(OAc)_5_ and 17-DMAG, caspase 3/7 activity was significantly augmented compared to thioQ(OAc)_4_ and thioQ(OAc)_5_ treatment, respectively (Fig. 3A). Thus, among fourteen QDs analyzed, we have documented three thioquercetin (thioQ, thioQ(OAc)_4_ and thioQ(OAc)_5_) with antimelanoma activity and no activity against normal control cells, and whose cytotoxicity may be enhanced upon HSP90 inhibition (Fig. 3B).

### Thioquercetin-based antimelanoma activity is associated with oxidative stress

**Fig. 4:**
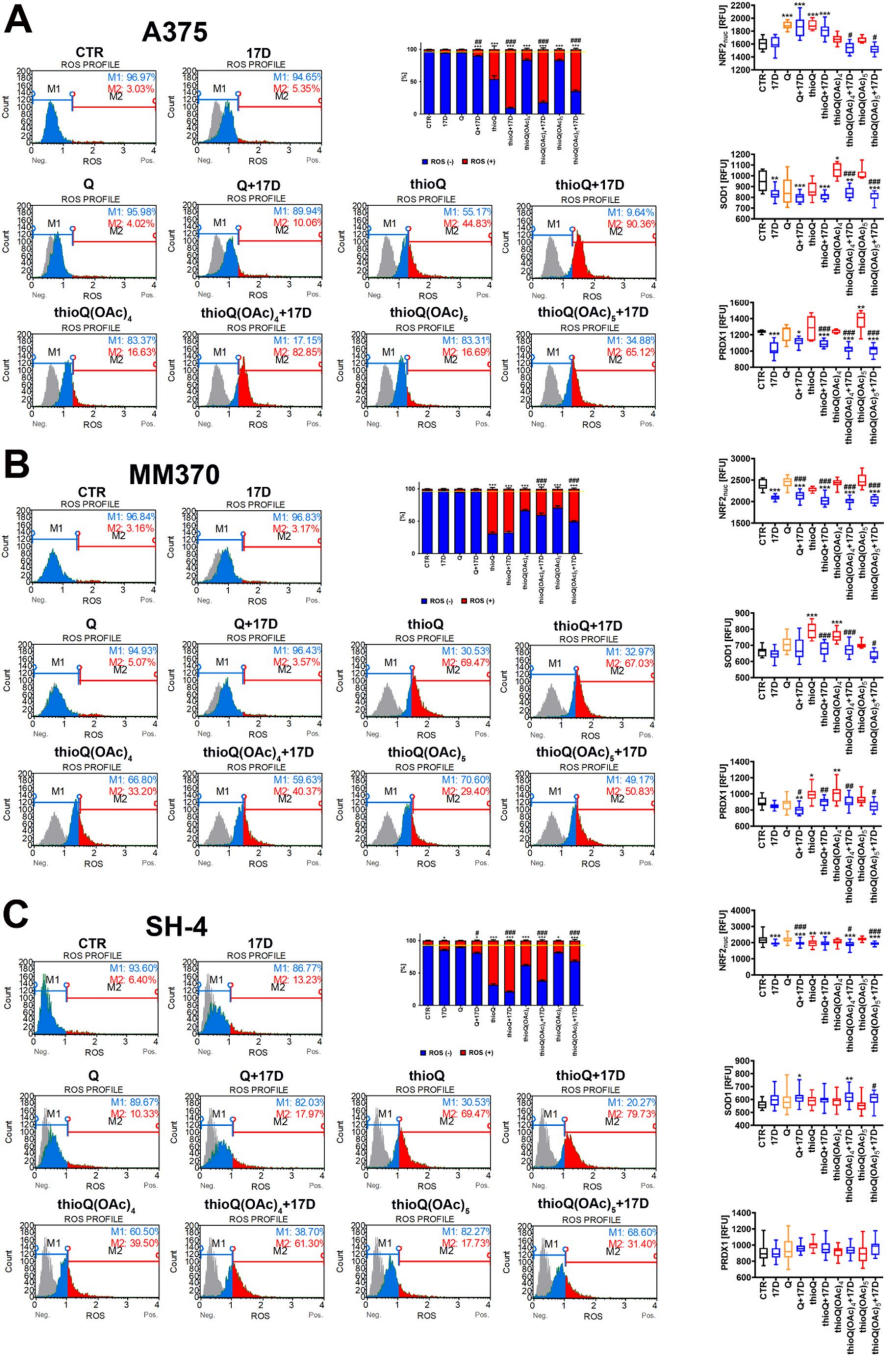
Thioquercetin-induced oxidative stress and related adaptive response in three melanoma cell lines (A375 (**A**), MM370 (**B**), and SH-4 cells (**C**)). Synergistic action of HSP90 inhibition was also considered. Cells were co-treated with 5 µM quercetin or thioquercetins (thioQ, thioQ(OAc)_4_, and thioQ(OAc)_5_) and 100 nM 17-DMAG, a HSP90 inhibitor, for 24 h. Oxidative stress was analyzed as the levels of intracellular superoxide using dihydroethidium staining and flow cytometry. Bars indicate SD, *n* = 3, ***p < 0.001, *p < 0.05 compared to control (CTR) (ANOVA and Dunnett’s a posteriori test), ^###^*p* < 0.001, ^##^*p* < 0.01, ^#^*p* < 0.05 compared to the treatment with quercetin or corresponding thioquercetin derivative (ANOVA and Tukey’s a posteriori test). A yellow horizontal line is used to emphasize the action of quercetin derivatives and HSP90 inhibitor compared to CTR. Representative histograms are also shown. Blue histograms (M1) indicate superoxide-negative subpopulation (ROS(-)) and red histograms (M2) denote superoxide-positive subpopulation (ROS(+)). A gray histogram (CTR control sample) is also included in each graph for comparison. The activation of a master regulator of antioxidant adaptive response, namely NRF2 transcription factor and the levels of selected antioxidant enzymes SOD1 and PRDX1 were also studied using dedicated antibodies, immunofluorescence protocol, and imaging cytometry. The nuclear levels of NRF2 and total levels of SOD1 and PRDX1 are presented as relative fluorescence units (RFU). Box and whisker plots are shown, *n* = 3, ***p < 0.001, **p < 0.01, *p < 0.05 compared to control (CTR) (ANOVA and Dunnett’s a posteriori test), ^###^*p* < 0.001, ^##^*p* < 0.01, ^#^*p* < 0.05 compared to the treatment with quercetin or corresponding thioquercetin derivative (ANOVA and Tukey’s a posteriori test). CTR, control conditions; Q, quercetin treatment; 17D, treatment with the HSP90 inhibitor alvespimycin (17-DMAG)

In terms of quercetin-mediated changes in intracellular redox milieu, the biphasic nature of quercetin was established [5, 11]. At lower concentrations (< 40 µM), quercetin was noticed to be a potent scavenger of ROS as a result of the presence of phenolic hydroxy groups on the B-ring and at the 3-position being in turn a cancer chemopreventive agent [5, 11]. At higher concentrations (> 40 µM), quercetin may be oxidized to produce reactive pro-oxidant o-quinone derivatives stimulating apoptotic cell death in cancer cells and sensitizing cancer cells to chemotherapeutic treatment [5, 11]. However, the effects of quercetin on redox homeostasis may be more complex. Quercetin may activate NRF2, a basic leucine zipper transcription factor orchestrating oxidative stress responses via the stimulation of antioxidant gene expression such as glutathione peroxidases, peroxiredoxins, and thioredoxins, glutathione synthesis genes or genes involved in drug detoxification such as glutathione *S*-transferases or members of the cytochrome P450 superfamily of enzymes [5, 36–38]. Quercetin-mediated changes in NRF2 activity may have cellular context-dependent effects in cancer cells, including melanoma [5, 36–38]. Thus, we then studied if thioquercetins may also modulate intracellular redox equilibrium by analyzing the levels of superoxide, a sign of oxidative stress when elevated. Treatment with thioquercetins resulted in increased levels of superoxide, being thioQ the most potent inducer of oxidative stress in all three melanoma cell lines used (Fig. 4A–C). In most cases, co-treatment with 17-DMAG potentiated thioquercetin-mediated oxidative stress (Fig. 4A–C). We postulate that oxidative stress may be associated with thioquercetin-induced cytotoxicity in melanoma cells (Figs. 2A and 4). Thus, to analyze the cellular response to thioquercetin-mediated oxidative stress more comprehensively, the activation of two transcription factors, namely NRF2 [38] and forkhead box class O3a (FOXO3a) [39] and their related selected target genes was investigated (Fig. 4 and Fig. S47). Unexpectedly, thioquercetin-based effects on the activity of NRF2 and FOXO3a were limited (Fig. 4 and Fig. S47). Increased nuclear pools of NRF2 and FOXO3a were observed in thioQ-treated A375 cells and thioQ(OAc)_4_-treated MM370 cells, respectively (Fig. 4A and Fig. S47). However, HSP90 inhibition and co-treatment conditions caused a decrease in the nuclear levels of NRF2 in all experimental settings in MM370 and SH-4 cells. In A375 cells, co-treatment with 17-DMAG and thioQ(OAc)_4_ or thioQ(OAc)_5_ also promoted a decrease in the nuclear pools of NRF2 compared to thioQ(OAc)_4_ or thioQ(OAc)_5_ treatments (Fig. 4A). Thus, HSP90 inhibition may limit NRF2-based antioxidative response in thioquercetin-treated melanoma cells leading to oxidative stress-related cytotoxicity. Indeed, HSP90 inhibition resulted in diminished levels of cytoplasmic superoxide dismutase SOD1, which neutralizes superoxide anions, and of two peroxide-eliminating enzymes peroxiredoxins PRDX1 and PRDX2 [40] in thioquercetin-treated A375 and MM370 cells (Fig. 4A and B, and Fig. S47).

Conversely, when HSP90 activity was not affected, thioQ(OAc)_4_ or thioQ(OAc)_5_ treatments caused an increase in the levels of SOD1 and PRDX1 in MM370 cells (Fig. 4B), whereas thioQ(OAc)_4_ and thioQ(OAc)_5_ stimulation elevated the levels of SOD1 and PRDX1 in A375 cells, respectively (Fig. 4A). In contrast, treatments with thioquercetins did not affect the levels of mitochondrial SOD2 in all three melanoma cell lines (Fig. S47). Thus, one can conclude that thioquercetin-based adaptive response is rather associated with cytoplasmic antioxidant proteins than mitochondrial ones, and mitochondrial compartment is not affected by thioquercetin-induced oxidative stress.

### Thioquercetins are detoxified using AhR/CYP1A1 pathway that is affected by HSP90 inhibition

**Fig. 5:**
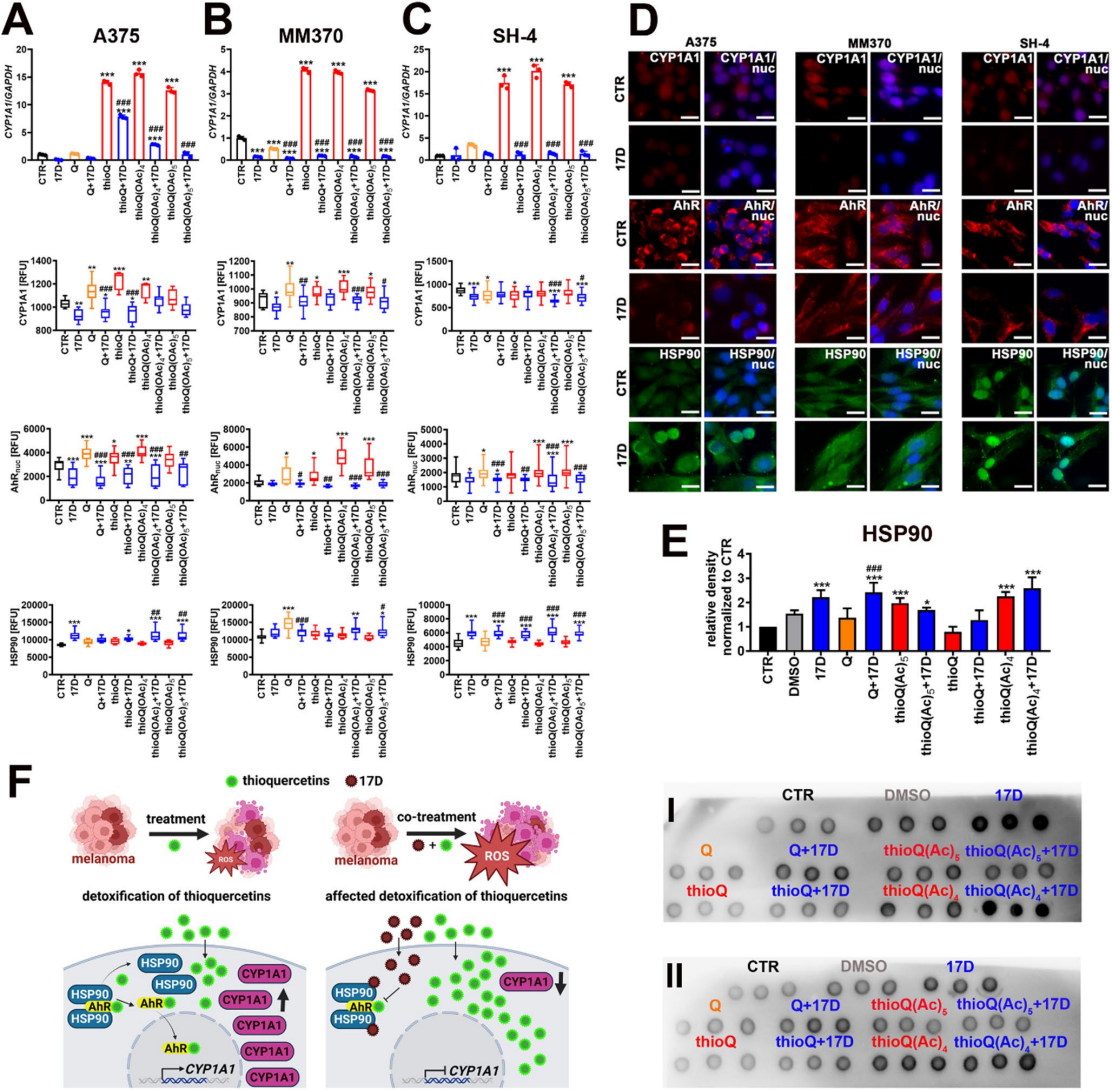
The activation of AhR/CYP1A1 detoxification-based signaling pathway upon stimulation with thioquercetins and its attenuation by HSP90 inhibition in melanoma cells (A375 (**A**), MM370 (**B**), and SH-4 cells (**C**)). Cells were co-treated with 5 µM quercetin or thioquercetins (thioQ, thioQ(OAc)_4_, and thioQ(OAc)_5_) and 100 nM 17-DMAG, a HSP90 inhibitor, for 24 h. For the analysis of AhR activation, cells were treated for 6 h. *CYP1A1* mRNA levels were investigated using real-time PCR and a dedicated probe. *GAPDH* served as a housekeeping gene. Expression data are presented as the ratio of *CYP1A1* to *GAPDH*. The protein levels of CYP1A1, AhR, and HSP90 were evaluated using dedicated antibodies, immunofluorescence protocol, and imaging cytometry. Nuclear levels of AhR and total levels of CYP1A1 and HSP90 are presented as relative fluorescence units (RFU). Bars indicate SD or box and whisker plots are shown, *n* = 3, ***p < 0.001, **p < 0.01, *p < 0.05 compared to control (CTR) (ANOVA and Dunnett’s a posteriori test), ^###^*p* < 0.001, ^##^*p* < 0.01, ^#^*p* < 0.05 compared to the treatment with quercetin or corresponding thioquercetin derivative (ANOVA and Tukey’s a posteriori test). CTR, control conditions; Q, quercetin treatment; 17D, treatment with a HSP90 inhibitor alvespimycin (17-DMAG). (**D**) Representative microphotographs of CYP1A1 (red), AhR (red), and HSP90 (green) immunostaining in three melanoma cell lines in the control conditions (CTR) and upon stimulation with a HSP90 inhibitor alvespimycin (denoted as 17D). Nuclei were visualized using Hoechst 33342 staining (blue). (**E**) Interaction analysis between a HSP90 inhibitor alvespimycin (100 nM) and/or thioquercetins (5 µM) and HSP90 (50 ng) in a cell-free system upon incubation at 37 °C overnight using dot-blot approach and dedicated antibodies. Results are presented as relative density units normalized to control (CTR). Representative dot-blots are shown (two replicates I and II). The effect of the solvent used (DMSO) is also included. Bars indicate SD, *n* = 3, ***p < 0.001, *p < 0.05 compared to control (CTR) (ANOVA and Dunnett’s a posteriori test), ^###^*p* < 0.001 compared to the treatment with quercetin or corresponding thioquercetin derivative (ANOVA and Tukey’s a posteriori test). CTR, control conditions; DMSO, solvent treatment; Q, quercetin treatment; 17D, treatment with the HSP90 inhibitor alvespimycin (17-DMAG). (**F**) A summarizing scheme showing the basis of AhR/CYP1A1-associated detoxification of thioquercetins and impaired detoxification upon HSP90 inhibition in melanoma cells. Thioquercetins are proposed as novel ligands of AhR stimulating its nuclear translocation and transcriptional activation of *CYP1A1* gene expression and corresponding CYP1A1 protein leading to CYP1A1-mediated detoxification of thioquercetins. Co-treatment with a HSP90 inhibitor alvespimycin (denoted as 17D) stabilized HSP90 and restricted AhR translocation to the nucleus resulting in attenuated expression of *CYP1A1* and CYP1A1-based detoxification of thioquercetins, and the subsequent accumulation of thioquercetins in melanoma cells leading to oxidative stress-associated cytotoxicity

Xenobiotics such as environmental pollutants, drugs, and selected dietary factors may stimulate the adaptive detoxification pathway, namely the AhR canonical transduction signaling by acting as AhR ligands [41, 42]. Upon ligand binding, AhR is translocated to the nucleus to serve as a transcription factor inducing, among others, the expression of xenobiotic metabolizing cytochrome P450 monooxygenases (P450s, CYPs) [41, 42]. In contrast, when not activated by a ligand, AhR stays in the cytoplasm as a component of multiprotein complex consisting of HSP90, HSP90-associated co-chaperone p23, AhR-interacting protein (AIP), and tyrosine kinase c-Src [41, 42]. Thus, we decided to analyze if also thioquercetins may be detoxified using an AhR-mediated mechanism. Treatment with the three thioquercetins for 24 h resulted in elevated mRNA levels of *CYP1A1*, a cytochrome P450 1A1, in all melanoma cell lines used (Fig. 5A–C). In contrast, co-treatment with 17-DMAG abolished CYP1A1-mediated response (Fig. 5A–C). Similar observations were also noticed in selected experimental settings at protein levels of CYP1A1 (Fig. 5A–D). We then asked the question of whether thioquercetin-mediated increase in *CYP1A1* expression is associated with AhR activation, thus, the nuclear levels of AhR were examined in treated and co-treated melanoma cells (Fig. 5A–D). Quercetin and thioquercetins caused an increase in the pools of nuclear AhR in all melanoma cell lines upon 6 h stimulation (Fig. 5A–D). HSP90 inhibition itself and HSP90 inhibition in thioquercetin-treated A375 and SH-4 cells promoted a decrease in nuclear pools of AhR (Fig. 5A, C and D). Co-treatment with 17-DMAG and thioquercetins also decreased the nuclear levels of AhR in MM370 cells (Fig. 5B and D).

These results may suggest that HSP90 inhibition could attenuate AhR-mediated stimulation of *CYP1A1* expression in thioquercetin-treated melanoma cells (Fig. 5A–D). Co-treatment with 17-DMAG and thioquercetins also stabilized the levels of HSP90 in A375 and SH-4 cells (Fig. 5A, C and D). HSP90 accumulation was also observed in 17-DMAG-treated A375 and SH-4 cells and after co-treatment with 17-DMAG and thioQ(OAc)_4_ or thioQ(OAc)_5_ in MM370 cells (Fig. 5A–D). We then analyzed the interactions between a HSP90 inhibitor 17-DMAG and human recombinant protein HSP90 using a cell-free in vitro system and dot-blot approach (Fig. 5E). Indeed, co-treatment with 17-DMAG and HSP90 resulted in an increase in chemiluminescence signals compared to control conditions suggesting 17-DMAG-mediated stabilization of HSP90 (Fig. 5E). Similar observations were noticed when HSP90 was incubated with 17-DMAG and thioquercetins, especially thioQ(OAc)_4_ (Fig. 5E). We postulate that the presence of thioquercetins in melanoma cells activated their detoxification pathway by the release of AhR from HSP90-AhR cytoplasmic complexes, AhR migration to the nucleus, and subsequent AhR-mediated induction of *CYP1A1* expression (Fig. 5F). Quercetin also promoted AhR nuclear translocation in three melanoma cell lines and this effect was accompanied by increased protein levels of CYP1A1 in A375 and MM370 cells (Fig. 5A–C). However, more direct binding data or co-immunoprecipitation approach are needed to further validate such assumptions. Quercetin was already reported to be an AhR ligand in MCF-7 human breast cancer cells [43]. Quercetin activated AhR binding to an oligonucleotide containing the xenobiotic-responsive element (XRE) of the *CYP1A1* promoter resulting in a time- and concentration-dependent increase in mRNA levels of *CYP1A1* [43]. Quercetin-mediated stimulation of CYP1A1 levels was abolished in AhR-deficient MCF-7 cells, thus, the authors concluded that quercetin is detoxified in MCF-7 cells by means of AhR/CYP1A1 signaling pathway [43]. Furthermore, quercetin treatment also caused an increase in mRNA expression and protein levels of CYP1A1 in MCF-10 F human noncancerous breast epithelial cells [44]. This observation was suggested to be a part of protective mechanism against estrogen-induced breast carcinogenesis by modulated expression of estrogen-metabolizing enzymes, namely more potent activation of CYP1A1 yielding less genotoxic 2-hydroxyestradiol (2-OHE_2_) than CYP1B1 producing carcinogenic estrogen metabolite 4-hydroxyestradiol (4-OHE_2_) [44]. When melanoma cells were co-treated with 17-DMAG and thioquercetins, HSP90 inhibition resulted in limited AhR nuclear translocation and AhR-related stimulation of *CYP1A1* gene transcription (Fig. 5F), thus HSP90 inhibition compromised AhR/CYP1A1 pathway-based detoxification of thioquercetins leading to potentiated cytotoxicity as judged by elevated apoptotic cell death in co-treated melanoma cells (Fig. 3A). According to the best of our knowledge, there are no published data on the effects of 17-DMAG-mediated HSP90 inhibition on the activity of AhR pathway and related affected drug detoxification in cancer cells. As *CYP1A1* is overexpressed in selected types of cancer, such as breast, lung, and colon cancer, CYP1A1 inhibition and/or attenuated expression may be considered as an attractive anticancer strategy, prolonging the exposure to the CYP1A1-substate anticancer drugs overcoming drug resistance and/or limiting cancer cell survival [45, 46]. This is in agreement with our current data that HSP90 inhibition may enhance thioquercetin-induced cytotoxicity and oxidative stress in melanoma cells as a result of affected AhR activation and related diminished CYP1A1-mediated detoxification of thioquercetins. Recently, CYP1A1 status was correlated with drug resistance in melanoma as elevated levels of CYP1A1 were observed in melanoma cells resistant to BRAF/MEK inhibitors (vemurafenib and cobimetinib) [47]. Furthermore, sustained activation of AhR promoted the resistance to BRAF inhibitors in melanoma cells that restricted targeting the BRAF-V600E/K mutated kinase, a common driver of malignant phenotype in melanoma, and facilitated disease relapse [48]. The HSP90 inhibitor XL888 was also reported to be effective at reversing BRAF inhibitor resistance in melanoma by affecting the status of key kinases regulating the cell cycle progression and cell survival, namely Wee1, Akt, and cyclin-dependent kinase 4 (CDK4) [49].

It is also worth noting that a complex cellular context-dependent cross-interaction between AhR and NRF2 transduction signaling has been reported [41, 50]. Besides NRF2 pathway, AhR may also regulate the expression of key antioxidant genes, for example, the promoter region of *SOD1* gene was shown to contain both XRE as well as antioxidant response element (ARE) regulated by AhR and NFR2, respectively [41]. NRF2 may also regulate the expression and activity of cytochrome P450 system [51], NRF2 may be a downstream target of AhR [52], and mixed AhR/NRF2 activators such as quercetin may be distinguished [50]. However, in our experimental conditions, the effects of quercetin and thioquercetins on NRF2 activation were limited and thioquercetin-mediated increase in the levels of SOD1 in selected experimental settings was achieved by the mechanisms other than NRF2-mediated effects, for example as a result of FOXO3a activation and/or AhR activation (Figs. 4 and 5A–C, and Fig. S47). AhR-based elevated expression of *CYP1A1* may be also associated with the induction of oxidative stress upon stimulation with thioquercetins (Fig. 4) as ROS production is tightly linked to the catalytic cycle of CYPs [41]. Nevertheless, upon HSP90 inhibition, CYP1A1 levels were decreased and oxidative stress was potentiated in thioquercetin-treated melanoma cells (Figs. 4 and 5A–C). HSP90 inhibition itself did not promote oxidative stress in A375 and MM370 cells (Fig. 4). Perhaps attenuation of CYP1A1-mediated detoxification of thioquercetins upon co-treatment with 17-DMAG resulted in the accumulation of thioquercetins in melanoma cells promoting pro-oxidant activity of thioquercetins and related cell death (Figs. 3A and 4). Treatment with 17-AAG also enhanced oxidative stress induced by a plant-derived alkaloid piperlongumine in colon cancer cells [53]. Synergistic action of 17-AAG and piperlongumine sensitized colon cancer cells to cell death by promoting ER stress, c-Jun N-terminal kinase (JNK) activation, and DNA damage [53]. More studies are needed to reveal the exact mechanism of enhanced oxidative stress as a result of combined treatment of 17-DMAG and thioquercetins in melanoma cells.

### Thioquercetins promote apoptotic cell death in drug-induced senescent melanoma cells

**Fig. 6:**
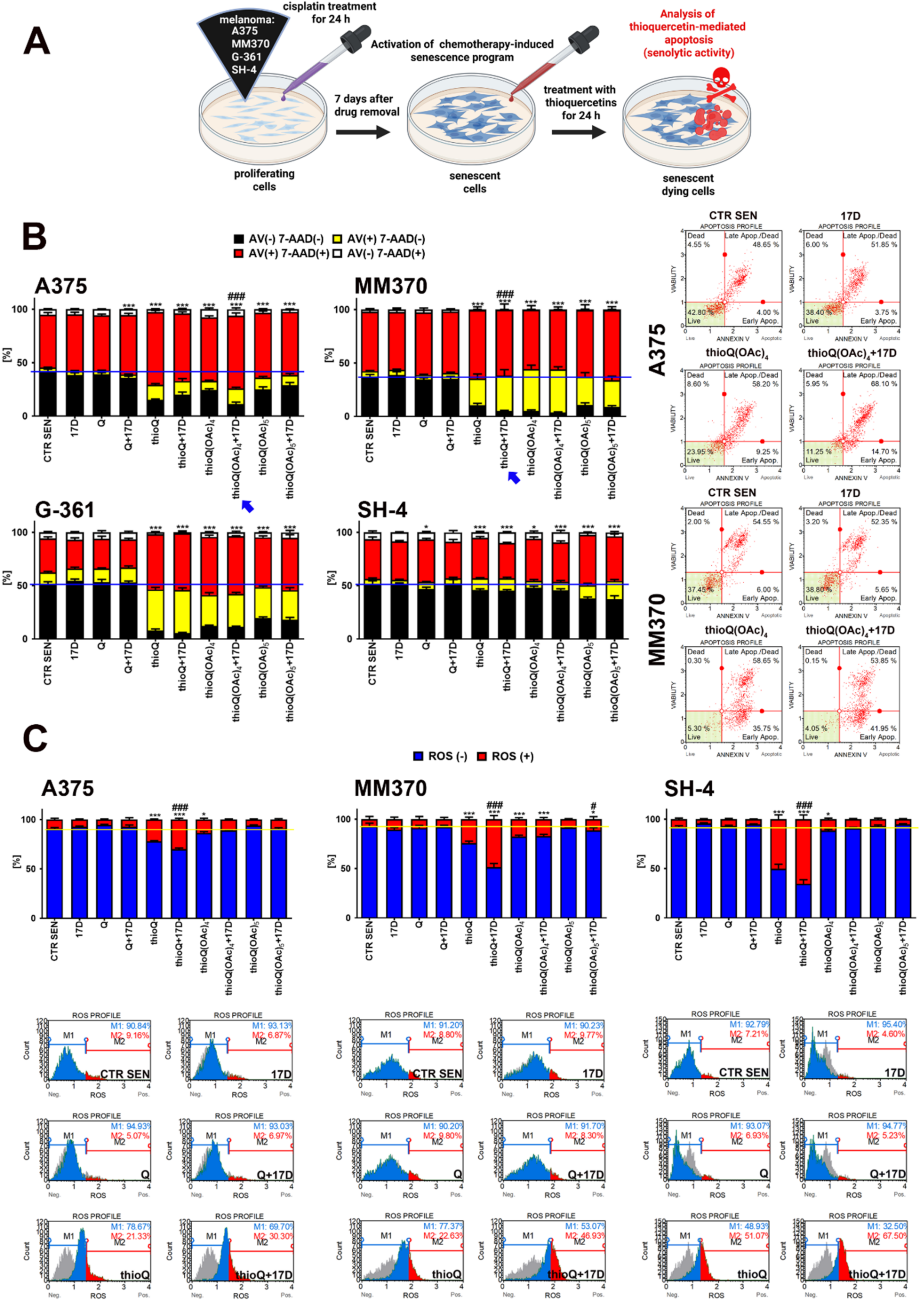
Thioquercetin-mediated senolytic activity (**A**,** B**) and oxidative stress (**C**) in drug-induced senescent melanoma cells. Synergistic action of HSP90 inhibition was also considered. (**A**) To activate drug-induced senescence program, melanoma cells were treated with cisplatin (2–5 µM depending on cell line used) for 24 h, and then the drug was removed, and cells were cultured for additional 7 days for the development of cisplatin-induced senescence phenotype. Senescent cells were then co-treated with 5 µM quercetin or thioquercetins (thioQ, thioQ(OAc)_4_, and thioQ(OAc)_5_) and 100 nM 17-DMAG, a HSP90 inhibitor, for 24 h to analyze senolytic activity of tested compounds. (**B**) Apoptosis and necrosis were evaluated using dual staining based on Annexin V staining and 7-AAD staining, and flow cytometry. Bars indicate SD, *n* = 3, ***p < 0.001, *p < 0.05 compared to senescence control (CTR SEN) (ANOVA and Dunnett’s a posteriori test), ^###^*p* < 0.001 compared to the treatment with quercetin or corresponding thioquercetin derivative (ANOVA and Tukey’s a posteriori test). A blue horizontal line is used to emphasize the action of quercetin derivatives and HSP90 inhibitor compared to CTR SEN. Representative dot-plots are also shown. Blue arrows indicate the potentiated senolytic activity upon co-treatment. (**C**) Oxidative stress was analyzed as the levels of intracellular superoxide using dihydroethidium staining and flow cytometry. Bars indicate SD, *n* = 3, ***p < 0.001, *p < 0.05 compared to senescence control (CTR SEN) (ANOVA and Dunnett’s a posteriori test), ^###^*p* < 0.001, ^#^*p* < 0.05 compared to the treatment with quercetin or corresponding thioquercetin derivative (ANOVA and Tukey’s a posteriori test). A yellow horizontal line is used to emphasize the action of quercetin derivatives and HSP90 inhibitor compared to CTR SEN. Representative histograms are also shown. Blue histograms (M1) indicate superoxide-negative subpopulation (ROS(-)) and red histograms (M2) denote superoxide-positive subpopulation (ROS(+)). A gray histogram (CTR SEN control sample) is also included in each graph for comparison. CTR SEN, control senescence conditions; Q, quercetin treatment; 17D, treatment with the HSP90 inhibitor alvespimycin (17-DMAG)

As chemotherapy-induced senescent cancer cells are considered as drug-resistant and hard-to-treat cancer cells [54–56], and the elimination of senescent cells (senolysis) by senolytic drugs is proposed a promising senotherapy and anticancer therapy [57–59], we decided then to investigate if the treatment with thioquercetins may also promote apoptotic cell death in drug-induced senescent melanoma cells and if HSP90 inhibition may have a synergistic senolytic effect upon thioquercetin stimulation in senescent melanoma cells. To activate chemotherapy-induced senescence program in melanoma cells, cisplatin treatment was used [21] (Fig. 6A). This in vitro senescence model was previously validated by us using eight melanoma cell lines and several senescence-related markers [21]. Thioquercetins induced the most pronounced apoptotic cell death in cisplatin-induced senescent G-361 cells (Fig. 6B). However, this melanoma cell line was also the most sensitive to the exposure of thioquercetins at proliferating state (Fig. 3A). Thus, thioquercetins are active against both proliferating and non-proliferating (senescent) G-361 cells (Figs. 3A and 6B). In contrast, the effects of thioQ(OAc)_4_ against proliferating MM370 cells were limited, whereas thioQ(OAc)_4_ promoted elevated apoptotic cell death in cisplatin-induced senescent MM370 cells (Figs. 3A and 6B). Furthermore, drug-induced senescent A375 cells were also more prone to thioQ(OAc)_4_ treatment than proliferating ones (Figs. 3A and 6B). Thus, senolytic activity of thioQ(OAc)_4_ was documented in selected experimental settings (Fig. 6B). HSP90 inhibition also potentiated senolytic effect of thioQ(OAc)_4_ in drug-induced senescent A375 cells (Fig. 6B). However, when 100 nM 17-DMAG was used alone, no elimination of cisplatin-induced senescent melanoma cells was observed (Fig. 6B). Thus, 17-DMAG did not possess senolytic activity in our experimental conditions. In contrast, N-terminal HSP90 inhibitors, including 17-DMAG, when used at the same concentration, were able to kill selectively senescent normal mouse fibroblasts that was achieved by 17-DMAG-mediated inhibition of the activity of HSP90 client protein, namely a pro-survival kinase Akt [60]. We have also previously observed a mild senolytic activity of 17-DMAG against etoposide-induced senescent A431 skin cancer cells that was potentiated when 17-DMAG was delivered as a component of iron oxide nanoparticle-based nanodevice targeting CD26, a senescent-associated cell surface marker using a dedicated anti-CD26 antibody [19].

Thioquercetins also induced oxidative stress in drug-induced senescent melanoma cells as judged by increased levels of superoxide, but this pro-oxidative effect was less accented than in thioquercetin-treated proliferating melanoma cells (Figs. 4 and 6C). Similar to proliferating melanoma cells, thioQ was the most potent inducer of oxidative stress in all senescent melanoma cell lines used and HSP90 inhibition enhanced pro-oxidative action of thioQ (Figs. 4 and 6C). Nevertheless, we did not address the involvement of AhR/CYP1A1 pathway during thioquercetin-mediated response in drug-induced senescent melanoma cells that should be treated as future research directions. We have also tested the senolytic activity of other eleven QDs (Fig. S48) with documented limited effects on proliferating melanoma cells (Fig. 2A). However, these QDs, when used at the concentrations of 5 and 10 µM, did not affect the viability of drug-induced senescent melanoma cells or their effects were minor (Fig. S48).

In conclusion, fourteen newly synthesized QDs were tested in terms of their antimelanoma activity and three thioquercetins, namely thioQ, thioQ(OAc)_4_ and thioQ(OAc)_5_, were found active against melanoma cells in vitro, when used at low micromolar range (up to 10 µM), compared to control normal cells. Thioquercetins also possessed antiproliferative potential against 3D melanoma spheroids as judged by thioquercetin-mediated decrease in A375 cell-based spheroid area and number. Antimelanoma effects of thioquercetins were potentiated upon HSP90 inhibition that was manifested by elevated oxidative stress and compromised AhR/CYP1A1-based detoxification of thioquercetins. In selected experimental settings, senolytic activity of 5 µM thioQ(OAc)_4_ against drug-induced senescent (non-proliferating) melanoma cells was also documented. Thus, cellular-context dependent antimelanoma effects of thioquercetins were shown along with proposed mechanism. However, our study has several limitations. Improved effectiveness of thioquercetins compared to parent compound should be also studied in terms of cellular uptake and bioavailability in vitro. More experiments are also needed to validate the antimelanoma activity of thioquercetins in vivo. Moreover, from mechanistic point of view, AhR/CYP1A1 pathway dependence and oxidative stress-mediated cytotoxicity upon co-treatment with thioquercetins and HSP90 inhibitor should be further verified using e.g., pharmacological and/or genetic inhibition of CYP1A1 and AhR, the analysis of related protein-protein interactions, and rescue assays using antioxidant treatment, respectively. We are convinced that these future research directions would allow for the design of novel antimelanoma strategies, especially based on HSP90 inhibition-mediated synergistic effects.

## Supplementary Information

Below is the link to the electronic supplementary material.

Supplementary Material 1

## Data Availability

The data presented in this study are available in the Supporting Information.

## References

[1] Lo JA, Fisher DE (2014) The melanoma revolution: from UV carcinogenesis to a new era in therapeutics. Science 346:945–949. 10.1126/science.1253735 25414302 10.1126/science.1253735 PMC4701046

[2] Ostrowski SM, Fisher DE (2021) Biology of melanoma. Hematol Oncol Clin North Am 35:29–56. 10.1016/j.hoc.2020.08.010 33759772 10.1016/j.hoc.2020.08.010

[3] Long GV, Swetter SM, Menzies AM et al (2023) Cutaneous melanoma. Lancet 402:485–502. 10.1016/S0140-6736(23)00821-8 37499671 10.1016/S0140-6736(23)00821-8

[4] Boutros A, Croce E, Ferrari M et al (2024) The treatment of advanced melanoma: Current approaches and new challenges. Crit Rev Oncol Hematol 196:104276. 10.1016/j.critrevonc.2024.104276 38295889 10.1016/j.critrevonc.2024.104276

[5] Harris Z, Donovan MG, Branco GM et al (2016) Quercetin as an emerging anti-melanoma agent: a four-focus area therapeutic development strategy. Front Nutr. 10.3389/fnut.2016.00048 10.3389/fnut.2016.00048 PMC5086580 27843913

[6] Thangasamy T, Sittadjody S, Lanza-Jacoby S et al (2007) Quercetin selectively inhibits bioreduction and enhances apoptosis in melanoma cells that overexpress tyrosinase. Nutr Cancer 59:258–268. 10.1080/01635580701499545 18001220 10.1080/01635580701499545

[7] Lu M, Miller P, Lu X (2014) Restoring the tumour suppressive function of p53 as a parallel strategy in melanoma therapy. FEBS Lett 588:2616–2621. 10.1016/j.febslet.2014.05.008 24844434 10.1016/j.febslet.2014.05.008

[8] Rauf A, Imran M, Khan IA et al (2018) Anticancer potential of quercetin: a comprehensive review. Phytother Res 32:2109–2130. 10.1002/ptr.6155 30039547 10.1002/ptr.6155

[9] Rajesh RU, Sangeetha D (2024) Therapeutic potentials and targeting strategies of quercetin on cancer cells: challenges and future prospects. Phytomedicine 133:155902. 10.1016/j.phymed.2024.155902 39059266 10.1016/j.phymed.2024.155902

[10] Rafiq RA, Quadri A, Nazir LA et al (2015) A potent inhibitor of phosphoinositide 3-kinase (PI3K) and mitogen activated protein (MAP) kinase signalling, quercetin (3, 3’, 4’, 5, 7-pentahydroxyflavone) promotes cell death in ultraviolet (UV)-B-irradiated B16F10 melanoma cells. PLoS ONE 10:e0131253. 10.1371/journal.pone.0131253 26148186 10.1371/journal.pone.0131253 PMC4493061

[11] Vargas AJ, Burd R (2010) Hormesis and synergy: pathways and mechanisms of quercetin in cancer prevention and management. Nutr Rev 68:418–428. 10.1111/j.1753-4887.2010.00301.x 20591109 10.1111/j.1753-4887.2010.00301.x

[12] Zang X, Cheng M, Zhang X, Chen X (2021) Quercetin nanoformulations: a promising strategy for tumor therapy. Food Funct 12:6664–6681. 10.1039/D1FO00851J 34152346 10.1039/d1fo00851j

[13] Joshi H, Gupta DS, Kaur G et al (2023) Nanoformulations of quercetin for controlled delivery: a review of preclinical anticancer studies. Naunyn Schmiedebergs Arch Pharmacol 396:3443–3458. 10.1007/s00210-023-02625-z 37490121 10.1007/s00210-023-02625-z

[14] Piotrowski P, Pawłowska J, Pawłowski J et al (2014) Nanostructured films of in situ deprotected thioacetyl-functionalized C60-fullerenes on a gold surface. J Mater Chem A 2:2353. 10.1039/c3ta13844e

[15] Ravishankar D, Watson KA, Greco F, Osborn HMI (2016) Novel synthesised flavone derivatives provide significant insight into the structural features required for enhanced anti-proliferative activity. RSC Adv 6:64544–64556. 10.1039/C6RA11041J

[16] Sui Z, Nguyen VN, Altom J et al (1999) Synthesis and topoisomerase inhibitory activities of novel aza-analogues of flavones. Eur J Med Chem 34:381–387. 10.1016/S0223-5234(99)80087-7

[17] Zima V, Radilová K, Kožíšek M et al (2020) Unraveling the anti-influenza effect of flavonoids: experimental validation of luteolin and its congeners as potent influenza endonuclease inhibitors. Eur J Med Chem 208:112754. 10.1016/j.ejmech.2020.112754 32883638 10.1016/j.ejmech.2020.112754

[18] Lewinska A, Adamczyk-Grochala J, Kwasniewicz E et al (2018) Reduced levels of methyltransferase DNMT2 sensitize human fibroblasts to oxidative stress and DNA damage that is accompanied by changes in proliferation-related miRNA expression. Redox Biol 14:20–34. 10.1016/j.redox.2017.08.012 28843151 10.1016/j.redox.2017.08.012 PMC5568885

[19] Wnuk M, Del Sol-Fernández S, Błoniarz D et al (2025) Design of a magnetic nanoplatform based on CD26 targeting and HSP90 inhibition for apoptosis and ferroptosis-mediated elimination of senescent cells. ACS Biomater Sci Eng 11:280–297. 10.1021/acsbiomaterials.4c00771 39631769 10.1021/acsbiomaterials.4c00771 PMC11733919

[20] Rzeszutek I, Cybularczyk-Cecotka M, Deręgowska A et al (2024) New mitochondria-targeted fisetin derivative compromises mitophagy and limits survival of drug-induced senescent breast cancer cells. J Med Chem 67:17676–17689. 10.1021/acs.jmedchem.4c01664 39322603 10.1021/acs.jmedchem.4c01664 PMC11472315

[21] Słaby J, Wnuk M, Błoniarz D et al (2025) ITGA1, the alpha 1 subunit of integrin receptor, is a novel marker of drug-resistant senescent melanoma cells in vitro. Arch Toxicol 99:2611–2625. 10.1007/s00204-025-04028-w 40202610 10.1007/s00204-025-04028-w

[22] Alizadeh SR, Ebrahimzadeh MA (2022) Quercetin derivatives: drug design, development, and biological activities, a review. Eur J Med Chem 229:114068. 10.1016/j.ejmech.2021.114068 34971873 10.1016/j.ejmech.2021.114068

[23] Martins IL, Charneira C, Gandin V et al (2015) Selenium-containing chrysin and quercetin derivatives: attractive scaffolds for cancer therapy. J Med Chem 58:4250–4265. 10.1021/acs.jmedchem.5b00230 25906385 10.1021/acs.jmedchem.5b00230

[24] Amorati R, Baschieri A, Valgimigli L (2019) The role of sulfur and heavier chalcogens in the chemistry of antioxidants. Phosphorus Sulfur Silicon Relat Elem 194:638–642. 10.1080/10426507.2019.1602620

[25] Alfieri ML, Panzella L, Amorati R et al (2022) Role of sulphur and heavier chalcogens on the antioxidant power and bioactivity of natural phenolic compounds. Biomolecules 12:90. 10.3390/biom12010090 35053239 10.3390/biom12010090 PMC8774257

[26] Walle T (2004) Absorption and metabolism of flavonoids. Free Radic Biol Med 36:829–837. 10.1016/j.freeradbiomed.2004.01.002 15019968 10.1016/j.freeradbiomed.2004.01.002

[27] Walle T, Ta N, Kawamori T et al (2007) Cancer chemopreventive properties of orally bioavailable flavonoids—Methylated versus unmethylated flavones. Biochem Pharmacol 73:1288–1296. 10.1016/j.bcp.2006.12.028 17250812 10.1016/j.bcp.2006.12.028 PMC1868573

[28] Przybylski P, Lewińska A, Rzeszutek I et al (2023) Mutation status and glucose availability affect the response to mitochondria-targeted quercetin derivative in breast cancer cells. Cancers 15:5614. 10.3390/cancers15235614 38067318 10.3390/cancers15235614 PMC10705313

[29] Shipp C, Weide B, Derhovanessian E, Pawelec G (2013) Hsps are up-regulated in melanoma tissue and correlate with patient clinical parameters. Cell Stress Chaperones 18:145–154. 10.1007/s12192-012-0363-1 22872370 10.1007/s12192-012-0363-1 PMC3581625

[30] Strickler AG, Vasquez JG, Yates N, Ho J (2014) Potential diagnostic significance of HSP90, ACS/TMS1, and L-plastin in the identification of melanoma. Melanoma Res 24:535–544. 10.1097/CMR.0000000000000115 25191796 10.1097/CMR.0000000000000115

[31] Mielczarek-Lewandowska A, Hartman ML, Czyz M (2020) Inhibitors of HSP90 in melanoma. Apoptosis 25:12–28. 10.1007/s10495-019-01577-1 31659567 10.1007/s10495-019-01577-1 PMC6965345

[32] Goel B, Jaiswal S, Tripathi N (2025) Recent advances in HSP90 inhibitors as targeted cancer therapy: chemical scaffolds, isoform selectivity, and clinical progress. Bioorg Chem 163:108782. 10.1016/j.bioorg.2025.108782 40706543 10.1016/j.bioorg.2025.108782

[33] Hsieh C-C, Shen C-H (2019) The potential of targeting P53 and HSP90 overcoming acquired MAPKi-resistant melanoma. Curr Treat Options Oncol 20:22. 10.1007/s11864-019-0622-9 30778775 10.1007/s11864-019-0622-9

[34] Shin MK, Jeong K-H, Choi H et al (2018) Heat shock protein 90 inhibitor enhances apoptosis by inhibiting the AKT pathway in thermal-stimulated SK-MEL-2 human melanoma cell line. J Dermatol Sci 90:357–360. 10.1016/j.jdermsci.2018.02.004 29433909 10.1016/j.jdermsci.2018.02.004

[35] Mielczarek-Lewandowska A, Sztiller-Sikorska M, Osrodek M et al (2019) 17-Aminogeldanamycin selectively diminishes IRE1α-XBP1s pathway activity and cooperatively induces apoptosis with MEK1/2 and BRAFV600E inhibitors in melanoma cells of different genetic subtypes. Apoptosis 24:596–611. 10.1007/s10495-019-01542-y 30989459 10.1007/s10495-019-01542-y PMC6598962

[36] Malakoutikhah Z, Mohajeri Z, Dana N, Haghjooy Javanmard S (2023) The dual role of Nrf2 in melanoma: a systematic review. BMC Mol Cell Biol 24:5. 10.1186/s12860-023-00466-5 36747120 10.1186/s12860-023-00466-5 PMC9900951

[37] Feng Q, Xu X, Zhang S (2024) Nrf2 protein in melanoma progression, as a new means of treatment. Pigment Cell Melanoma Res 37:247–258. 10.1111/pcmr.13137 37777339 10.1111/pcmr.13137

[38] Pei Y, Yin J, Liu J et al (2025) The role of NRF2 in human cancers: Pre-clinical insights paving the way for clinical trials. J Pharm Anal. 10.1016/j.jpha.2025.101358 10.1016/j.jpha.2025.101358 PMC13199830 42199534

[39] Liu Y, Wang Y, Li X et al (2022) FOXO3a in cancer drug resistance. Cancer Lett 540:215724. 10.1016/j.canlet.2022.215724 35545128 10.1016/j.canlet.2022.215724

[40] Liu Y, Wang P, Hu W, Chen D (2023) New insights into the roles of peroxiredoxins in cancer. Biomed Pharmacother 164:114896. 10.1016/j.biopha.2023.114896 37210897 10.1016/j.biopha.2023.114896

[41] Vogel CFA, Van Winkle LS, Esser C, Haarmann-Stemmann T (2020) The aryl hydrocarbon receptor as a target of environmental stressors – Implications for pollution mediated stress and inflammatory responses. Redox Biol 34:101530. 10.1016/j.redox.2020.101530 32354640 10.1016/j.redox.2020.101530 PMC7327980

[42] Sondermann NC, Faßbender S, Hartung F et al (2023) Functions of the aryl hydrocarbon receptor (AHR) beyond the canonical AHR/ARNT signaling pathway. Biochem Pharmacol 208:115371. 10.1016/j.bcp.2022.115371 36528068 10.1016/j.bcp.2022.115371 PMC9884176

[43] Ciolino HP, Daschner PJ, Yeh GC (1999) Dietary flavonols quercetin and kaempferol are ligands of the aryl hydrocarbon receptor that affect CYP1A1 transcription differentially. Biochem J 340(Pt 3):715–722 10359656 PMC1220303

[44] Mense SM, Chhabra J, Bhat HK (2008) Preferential induction of cytochrome P450 1A1 over cytochrome P450 1B1 in human breast epithelial cells following exposure to quercetin. J Steroid Biochem Mol Biol 110:157–162. 10.1016/j.jsbmb.2008.03.029 18456490 10.1016/j.jsbmb.2008.03.029 PMC2533731

[45] Dai Z, Wu Y, Xiong Y et al (2024) CYP1A inhibitors: recent progress, current challenges, and future perspectives. Med Res Rev 44:169–234. 10.1002/med.21982 37337403 10.1002/med.21982

[46] Rodriguez M, Potter DA (2013) CYP1A1 regulates breast cancer proliferation and survival. Mol Cancer Res 11:780–792. 10.1158/1541-7786.MCR-12-0675 23576571 10.1158/1541-7786.MCR-12-0675 PMC3720830

[47] Kot M, Simiczyjew A, Wądzyńska J et al (2024) Characterization of two melanoma cell lines resistant to BRAF/MEK inhibitors (vemurafenib and cobimetinib). Cell Commun Signal 22:410. 10.1186/s12964-024-01788-3 39175042 10.1186/s12964-024-01788-3 PMC11342534

[48] Corre S, Tardif N, Mouchet N et al (2018) Sustained activation of the Aryl hydrocarbon receptor transcription factor promotes resistance to BRAF-inhibitors in melanoma. Nat Commun 9:4775. 10.1038/s41467-018-06951-2 30429474 10.1038/s41467-018-06951-2 PMC6235830

[49] Haarberg HE, Paraiso KHT, Wood E et al (2013) Inhibition of Wee1, AKT, and CDK4 underlies the efficacy of the HSP90 inhibitor XL888 in an in vivo model of *NRAS* -mutant melanoma. Mol Cancer Ther 12:901–912. 10.1158/1535-7163.MCT-12-1003 23538902 10.1158/1535-7163.MCT-12-1003 PMC3683468

[50] Köhle C, Bock KW (2006) Activation of coupled Ah receptor and Nrf2 gene batteries by dietary phytochemicals in relation to chemoprevention. Biochem Pharmacol 72:795–805. 10.1016/j.bcp.2006.04.017 16780804 10.1016/j.bcp.2006.04.017

[51] Ashino T, Yamamoto M, Numazawa S (2020) Nrf2 antioxidative system is involved in cytochrome P450 gene expression and activity: a delay in pentobarbital metabolism in Nrf2-deficient mice. Drug Metab Dispos 48:673–680. 10.1124/dmd.120.000010 32503880 10.1124/dmd.120.000010

[52] Miao W, Hu L, Scrivens PJ, Batist G (2005) Transcriptional regulation of NF-E2 p45-related factor (NRF2) expression by the aryl hydrocarbon receptor-xenobiotic response element signaling pathway. J Biol Chem 280:20340–20348. 10.1074/jbc.M412081200 15790560 10.1074/jbc.M412081200

[53] Qiu C, Shen X, Lu H et al (2023) Combination therapy with HSP90 inhibitors and piperlongumine promotes ROS-mediated ER stress in colon cancer cells. Cell Death Discov 9:375. 10.1038/s41420-023-01672-y 37833257 10.1038/s41420-023-01672-y PMC10576049

[54] Guillon J, Petit C, Toutain B et al (2019) Chemotherapy-induced senescence, an adaptive mechanism driving resistance and tumor heterogeneity. Cell Cycle 18:2385–2397. 10.1080/15384101.2019.1652047 31397193 10.1080/15384101.2019.1652047 PMC6738909

[55] Thompson EL, Hu JJ, Niedernhofer LJ (2021) The role of senescent cells in acquired drug resistance and secondary cancer in BRAFi-treated melanoma. Cancers 13:2241. 10.3390/cancers13092241 34066966 10.3390/cancers13092241 PMC8125319

[56] Wang B, Kohli J, Demaria M (2020) Senescent cells in cancer therapy: friends or foes? Trends Cancer 6:838–857. 10.1016/j.trecan.2020.05.004 32482536 10.1016/j.trecan.2020.05.004

[57] Wang L, Lankhorst L, Bernards R (2022) Exploiting senescence for the treatment of cancer. Nat Rev Cancer. 10.1038/s41568-022-00450-9 35241831 10.1038/s41568-022-00450-9

[58] Power H, Valtchev P, Dehghani F, Schindeler A (2023) Strategies for senolytic drug discovery. Aging Cell 22:e13948. 10.1111/acel.13948 37548098 10.1111/acel.13948 PMC10577556

[59] Zhang L, Pitcher LE, Prahalad V et al (2023) Targeting cellular senescence with senotherapeutics: senolytics and senomorphics. FEBS J 290:1362–1383. 10.1111/febs.16350 35015337 10.1111/febs.16350

[60] Fuhrmann-Stroissnigg H, Ling YY, Zhao J et al (2017) Identification of HSP90 inhibitors as a novel class of senolytics. Nat Commun 8:422. 10.1038/s41467-017-00314-z 28871086 10.1038/s41467-017-00314-z PMC5583353

