# Supplementary material for: HSP90 inhibition potentiates oxidant-based antimelanoma action of novel thioquercetin derivatives by compromising AhR/CYP1A1 pathway
Source: Apoptosis. 2026 Mar 28;31(4):114. doi: 10.1007/s10495-026-02311-4 (PMC13032962; doi:10.1007/s10495-026-02311-4)
Supplement: Supplementary file 1 — Supplementary Material 1 [file 10495_2026_2311_MOESM1_ESM.docx]

**SUPPORTING INFORMATION**

**HSP90 inhibition potentiates oxidant-based antimelanoma action of novel thioquercetin derivatives by compromising AhR/CYP1A1 pathway**

Wojciech Witkowski^1#^, Julia Słaby^2,3#^, Maciej Wnuk^3^, Paulina Stec^3^, Piotr Piotrowski^1^, Michał Żebrowski^1^, Martyna Cybularczyk-Cecotka^1^, Anna Deręgowska^3^, Nadezhda Romanchikova^4^, Pawel Zayakin^4^, Aija Linē^4,5^, María Moros^6,7^, Grzegorz Litwinienko^1*^, Anna Lewińska^3*^

*^1^Faculty of Chemistry, University of Warsaw, Pasteura 1, 02-093 Warsaw, Poland*

*^2^Doctoral School, University of Rzeszow, Pigonia 1, 35-310 Rzeszow, Poland*

*^3^Faculty of Biotechnology, Collegium Medicum, University of Rzeszow, Pigonia 1, 35-310 Rzeszow, Poland*

*^4^Cancer Biomarker group, Latvian Biomedical Research and Study Centre, Ratsupites 1, LV-1067 Riga, Latvia*

*^5^Faculty of Medicine and Life Sciences, University of Latvia, Jelgavas 1, LV-1004 Riga, Latvia*

*^6^Instituto de Nanociencia y Materiales de Aragón, INMA (CSIC-Universidad de Zaragoza), C/Pedro Cerbuna 12, 50009 Zaragoza, Spain*

*^7^Centro de Investigación Biomédica en Red de Bioingeniería, Biomateriales y Nanomedicina (CIBER-BBN), 28029 Madrid, Spain.*

^#^Contributed equally

^*^Correspondence: Grzegorz Litwinienko ([litwin@chem.uw.edu.pl](mailto:litwin@chem.uw.edu.pl)), Anna Lewińska ([alewinska@ur.edu.pl](mailto:alewinska@ur.edu.pl)).

**Table of Contents**

|  | Page |
| --- | --- |
| **1. General information on the synthesis and characterization of quercetin derivatives.** | S-4 |
| **2. Preparation of quercetin methoxy derivatives.** | S-4 |
| **Scheme S1.** General route for synthesis of quercetin methoxy derivatives. | S-4 |
| 2.1. Compound Q(OMe)_5_ | S-4 |
| 2.2. Compound Q(OMe)_4_ | S-5 |
| 2.3. Compound N_3_-PEG-Q(OMe)_4_ | S-6 |
| 2.4 Compound 5-N_3_Bu-Q(OMe)_4_ | S-7 |
| **Figures S1-S4.** ^1^H spectra of quercetin methoxy derivatives. | S-8 |
| **3. Preparation of quercetin acetyl derivatives.** | S-10 |
| **Scheme S2.** Overall synthetic pathway for quercetin acetyl derivatives. | S-10 |
| 3.1. Compound 3-N_3_Bu-Q(OAc)_4_ | S-11 |
| **Figures S5-S14.** ^1^H and ^13^C NMR spectra of quercetin acetyl derivatives (part I). | S-15 |
| 3.2. Compound 5-N_3_Bu-Q(OAc)_4_ | S-20 |
| **Figures S15-S23.** ^1^H and ^13^C NMR spectra of quercetin acetyl derivatives (part II). | S-24 |
| 3.3. Compound 7-N_3_Bu-Q(OAc)_4_ | S-28 |
| **Figures S24-S30.** ^1^H and ^13^C NMR spectra of quercetin acetyl derivatives (part II). | S-32 |
| **4. Preparation of aza-quercetin (azaQ) and its derivatives.** | S-35 |
| 4.1. Compound azaQ(OMe)_5_ | S-35 |
| 4.2. Compound azaQ | S-37 |
| 4.3. Compound acyl-azaQ(OAc)_5_ | S-37 |
| **Figures S31-S38.** ^1^H and ^13^C NMR spectra of aza-quercetin derivatives. | S-38 |
| **5. Preparation of thioquercetin (thioQ)and its derivatives.** | S-43 |
| **Scheme S3.** Overall synthetic pathway for thioQ and its derivatives. | S-43 |
| 5.1. Compound thioQ | S-43 |
| 5.2. Compound thioQ(OMe)_5_ | S-44 |
| 5.3. Compound thioQ(OAc)_4_ | S-44 |
| 5.4. Compound thioQ(OAc)_5_ | S-46 |
| **Figures S39-S45.** ^1^H and ^13^C NMR spectra of thioquercetin (thioQ) and its derivatives. | S-47 |
| 5.5. Determination of purity | S-51 |
| 5.5.1. HPLC purity profile of thioQ | S-51 |
| 5.5.2. HPLC purity profile of thioQ(OAc)_4_ | S-55 |
| 5.5.3. HPLC purity profile of thioQ(OAc)_5_ | S-57 |
| **Figure S46.** Quercetin derivative-mediated apoptotic cell death in three melanoma cell lines (24 h treatment with 10 µM tested compounds). | S-60 |
| **Figure S47.** Thioquercetin-associated adaptive response to oxidative stress induction in three melanoma cell lines. The analysis of the levels of FOXO3a, SOD2, and PRDX2. | S-62 |
| **Figure S48.** Quercetin derivative-mediated senolytic activity in drug-induced senescent melanoma cells. | S-63 |
| **References** | S-64 |

**1. General information on the synthesis and characterization of quercetin derivatives.**

All reagents and solvents were purchased from commercial suppliers and used without further purification. Thin layer chromatography (TLC) was performed using Merck Silica Gel F254, 0.20 mm thickness and the visualization was accomplished by irradiation at 254 nm. All aqueous solutions were prepared using distilled water. Saturated brine refers to an aqueous saturated sodium chloride solution. All products were purified by column chromatography using silica gel 60 M (40-63 µm, 230-440 mesh). NMR spectra were recorded at room temperature using Bruker 300 MHz (^1^H NMR) or 75 MHz (^13^C NMR) spectrometer. Chemical shifts are reported relatively in δ-scale as parts per million (ppm) referenced to the residual solvent peak. Coupling constants *J* are given in Hertz (Hz) and the following abbreviations were used for indicating signal multiplicity: ^1^H NMR: s = singlet, d = doublet, t = triplet, q = quartet, hept = heptet, m = multiplet and the respective combinations.

**2. Preparation of quercetin methoxy derivatives.**

**Scheme S1.** General route for synthesis of quercetin methoxy derivatives.

**2.1.Compound Q(OMe)_5_**

*Full name: 2-(3,4-dimethoxyphenyl)-3,5,7-trimethoxy-4H-chromen-4-one*

Anhydrous quercetin (6.00 g, 19.9 mmol, 1 eq.) and anhydrous K_2_CO_3_ (110 g, 796 mmol, 40 eq.) were suspended in dry DMF (50 ml). After stirring for 15 min, methyl iodide was slowly added (37 ml, 596 mmol, 30 eq.). The mixture was allowed to heat upon exothermic reaction, vigorously shaken, and then stirred at room temperature (RT) for 2 h. The crude mixture was poured into ice-cold water and the precipitate was collected by filtration, washed extensively with water and dried *in vacuo*.

2-(3,4-dimethoxyphenyl)-3,5,7-trimethoxy-4H-chromen-4-one was obtained as a pale yellow solid (5.54 g, 14.9 mmol, 75%).

^1^H NMR (300 MHz, DMSO-*d*_6_) *δ* 7.71 – 7.61 (*m*, 2H, Ar-H), 7.13 (*d*, *J* = 8.6 Hz, 1H, Ar-H), 6.82 (*d*, *J* = 2.3 Hz, 1H, Ar-H), 6.48 (*d*, *J* = 2.3 Hz, 1H, Ar-H), 3.89 (*s*, 3H, OCH_3_), 3.85 (*s*, 3H, OCH_3_), 3,85 (*s*, 3H, OCH_3_) 3.84 (*s*, 3H, OCH_3_), 3.75 (*s*, 3H, OCH_3_). The spectrum is consistent with the literature [1].

**2.2. Compound Q(OMe)_4_**

*Full name: 2-(3,4-dimethoxyphenyl)-3,7-dimethoxy-4H-chromen-4-one*

Quercetin was alkylated to tetramethoxyquercetin (**Q**(**OMe**)**_4_**) with methyl iodide using method described previously in the literature [2]. To a well stirred solution of quercetin (302 mg, 1 mmol) in a mixture of methanol and acetonitrile (120 ml, 1:2, v/v) potassium carbonate (4.15 g, 30 mmol) along with methyl iodide (436 µl, 7 mmol) were added. Reaction was proceeded at 60^o^C for 6-8 h. Afterwards, solvents and methyl iodide were removed under reduced pressure. Resulting mixture containing mainly pentamethoxyquercetin and significantly lower amount of tetramethoxyquercetin was separated by column chromatography on silica gel using 4:1 mixture of dichloromethane and acetone. Desired product was recrystallized from ethanol, yield 29%.

^1^H NMR (300 MHz, CDCl_3_) *δ* 7.79-7.70(m, 1H, Ar-H), 7.38 (ABq, 2H, Δδ_AB_=214.21Hz, J_AB_=8.6Hz, Ar-H), 6.43 (ABq, 2H, Δδ_AB_= 26.39Hz, J_AB_=2.2Hz, Ar-H), 4.00 (*s*, 3H, OCH_3_), 3.99 (*s*, 3H, OCH_3_), 3.90 (*s*, 3H, OCH_3_) 3.89 (*s*, 3H, OCH_3_) ppm.

**2.3. Compound N_3_-PEG-Q(OMe)_4_**

*Full name: 5-(2-(2-(2-(2-azidoethoxy)ethoxy)ethoxy)ethoxy)-2-(3,4-dimethoxyphenyl)-3,7-dimethoxy-4H-chromen-4-one*

Alkylation was performed using bromo-PEG_3_-bromide (1-bromo-2-(2-(2-(2-bromoethoxy)ethoxy)ethoxy)ethane) and potassium carbonate in dimethylformamide using slightly modified procedure reported previously by our group [3]. Briefly, to a solution of bromo-PEG_3_-bromide (320 mg, 1 mmol) and potassium carbonate (69 mg, 0.5 mmol) in DMF (5 ml) at 80^o^C, a solution of Q(OMe)_4_ (89 mg, 0.25 mmol) in DMF (10 ml) was added dropwise. Reaction was then heated at 80^o^C for 2h under nitrogen atmosphere. After cooling to room temperature, mixture was filtrated to remove insoluble precipitate. After evaporation of the solvent from the filtrate, it was then washed with diethyl ether. Resulting crude product was proceeded to the next step without further purification [4]. To a solution of bromo-PEG functionalized Q(OMe)_4_ (99 mg, 0.17 mmol) in DMSO (5 ml), sodium azide (33 mg, 0.5 mmol) was added and obtained suspension was heated to 50^o^C under nitrogen atmosphere for 2 h. It was then filtered, diluted with deionized water, and dialyzed using MWCO 500 regenerated cellulose bag. Obtained solution was poured on Petri dish and solvent evaporated under nitrogen flow. Desired product was obtained as a pale yellow liquid, yield 24%.

^1^H NMR (300 MHz, DMSO) δ 7.71 – 7.63 (m, 1H, Ar-H), 7.41 (ABq, 2H, Δδ_AB_=155.20Hz, J_AB_=8.7Hz, Ar-H), 6.68 (ABq, 2H, Δδ_AB_= 95.80Hz, J_AB_=2.3Hz, Ar-H), 4.22 – 4.12 (m, 2H, CH_2_), 3.89 (s, 3H, OCH_3_), 3.86 (s, 3H, OCH_3_), 3.86 (s, 3H, OCH_3_), 3.76 (s, 3H, OCH_3_), 3.75-3.69 (m, 4H, CH_2_CH_2_), 3.61 – 3.53 (m, 10H, PEG).

**2.4 Compound 5-N_3_Bu-Q(OMe)_4_**

*Full name: 5-(4-azidobutoxy)-2-(3,4-dimethoxyphenyl)-3,7-dimethoxy-4H-chromen-4-one*

Similarly to N_3_-PEG-Q(OMe)_4_, its butyl analogue was synthesized in two steps using the same methods. In the first step tetramethoxyquercetin (**Q**(**OMe**)**_4_**) was alkylated with 1,4-dibromobutane. To a DMF (5 ml) solution of 1,4-dibromobutane (119 µl, 1 mmol) and K_2_CO_3_ (69 mg, 0.5 mmol) heated to 80^o^C, a solution of **Q(OMe)_4_** (89 mg, 0.25 mmol) dissolved in 10 ml of DMF was added dropwise. Reaction was continued at 80^o^C under N_2_ atmosphere for 2 h. Resulting mixture was allowed to cool to RT and was then filtrated. Afterwards, it was diluted with water and extracted with ethyl acetate. Organic layer was dried with sodium sulfate and evaporation of solvent yielded yellow solid. Obtained crude product was used in the next step without further purification. Bromobutyl functionalized Q(OMe)_4_ (84 mg, 0.17 mmol) was dissolved in DMSO (5 ml) and to the resulting solution sodium azide (33 mg, 0.5 mmol) was added. Obtained suspension was heated to 50^o^C under nitrogen atmosphere for 2 h. Reaction mixture was poured onto small amount of crushed ice and the resulting aqueous solution was then extracted with ethyl acetate. Combined extracts were dried over sodium sulfate and concentrated. Product was precipitated with hexane to yield light yellow powder, yield 91%.

^1^H NMR (300 MHz, CDCl_3_) δ 7.75 – 7.70 (m, 1H, Ar-H), 7.37 (ABq, 2H, Δδ_AB_=219.10Hz, J_AB_=9.2Hz, Ar-H), 6.44 (ABq, 2H, Δδ_AB_= 50.70Hz, J_AB_=2.3Hz, Ar-H), 4.13 (t, *J* = 5.9 Hz, 2H, CH_2_), 3.99 (s, 6H, OCH_3_), 3.92 (s, 3H, OCH_3_), 3.86 (s, 3H, OCH_3_), 3.60 (t, *J* = 6.4 Hz, 2H, CH_2_), 2.32-2.20 (m, 2H, CH_2_), 2.18-2.07 (m, 2H, CH_2_).

**Figure S1.** ^1^H NMR (300 MHz, DMSO-*d_6_*) of 2-(3,4-dimethoxyphenyl)-3,5,7-trimethoxy-4H-chromen-4-one (**Q(OMe)_5_**).

**Figure S2.** ^1^H NMR (300 MHz, CDCl_3_) spectrum of 2-(3,4-dimethoxyphenyl)-5-hydroxy-3,7-dimethoxy-4H-chromen-4-one (**Q(OMe)_4_**).

**Figure S3.** ^1^H NMR (300 MHz, DMSO) of 5-(2-(2-(2-(2-azidoethoxy)ethoxy)ethoxy)ethoxy) -2-(3,4-dimethoxyphenyl)-3,7-dimethoxy-4H-chromen-4-one (**N_3_-PEG-Q(OMe)_4_**).

**Figure S4.** ^1^H NMR (300 MHz, CDCl_3_) spectrum of 5-(4-azidobutoxy)-2-(3,4-dimethoxyphenyl)-3,7-dimethoxy-4H-chromen-4-one (**5-N_3_Bu-Q(OMe)_4_**).

**3. Preparation of quercetin acetyl derivatives.**

**Scheme S2.** Overall synthetic pathway for quercetin acetyl derivatives.

Conditions:

A) Ph_2_O, Ph_2_CCl_2_, 175^o^C, 6 h,

B) Ac_2_O, Et_3_N, DCM, 0°C → RT, 2 h,

C) Br(CH_2_)_4_Cl, K_2_CO_3_, DMF, RT, 20 h,

D) CH_3_COOH/H_2_O, reflux, 2 h,

E) Ac_2_O, Et_3_N, DCM, 0°C → RT, 72 h,

F) NaI, aceton, reflux, 20 h,

G) NaN_3_, DMF, RT, 1 h,

H) H_2_/Pd, EtOH/THF 1:1, RT, 20 h,

I) BnBr, K_2_CO_3_, DMF, 182^o^C, 2-3 h.

**3.1. Compound 3-N_3_Bu-Q(OAc)_4_**

*Full name: 3-(4-azidobutoxy)-2-(3,4-diacetoxyphenyl)-4-oxo-4H-chromene-5,7-diyl diacetate*

***Step A***

*formal name: 3′,4′-O-diphenylmethane quercetin 2-(2,2-diphenylbenzo[d][1,3]dioxol-5-yl)-3,5,7-trihydroxy-4H-chromen-4-one*

Quercetin (3.00 g, 8.87 mmol) and 1,1-dichlorodiphenylmethane (3.15 g, 13.3 mmol) were dissolved in diphenyl ether (100 ml) and the reaction mixture was heated to 182 °C with stirring. After 6 h, the mixture was cooled to RT and petroleum ether (250 ml) was added. The dark yellow crude product was obtained by filtration and purified by silica gel column chromatography (pure DCM as eluent) to give a product as a light yellow solid with isolated yield: 66% (2.73 g, 5.85 mmol).

^1^H NMR (300 MHz, CDCl_3_) δ 11.74 (s, 2H), 7.84 – 7.74 (m, 2H), 7.67 – 7.54 (m, 4H), 7.49 – 7.33 (m, 6H), 7.05 – 6.98 (m, 1H), 6.58 (s, 1H), 6.42 (d, *J* = 2.2 Hz, 1H), 6.29 (d, *J* = 2.2 Hz, 1H), 5.53 (s, 1H). The obtained spectral data are consistent with the literature reports [5].

***Step C***

*full name: 3-(4-O-chlorobutyl)-3′,4′-O-diphenylmethane quercetin or alternatively:*

*3-(4-chlorobutoxy)-2-(2,2-diphenylbenzo[d][1,3]dioxol-5-yl)-5,7-dihydroxy-4H-chromen-4-one*

3′,4′-O-diphenylmethane quercetin (1.51 g, 3.24 mmol) and K_2_CO_3_ (0.49 g, 3.56 mmol) were dissolved in DMF (10 ml) and 1-bromo-4-chlorobutane (0.83 g, 4.86 mmol) was added. The reaction mixture was stirred overnight at RT. After confirming by TLC that all substrate had reacted, the reaction mixture was diluted with ethyl acetate (100 ml), transfer into the separating funnel and washed 3 times with 50 ml of 1N HCl. Organic layer was dried with anhydrous MgSO_4_. The crude product was purified by column chromatography with elucidation by hexane : ethyl acetate (8:2) mixture to give 3-(4-*O*-chlorobutyl)-3′,4′-*O*-diphenylmethane quercetin as light yellow solid with isolated yield 36% (0.65 g, 1.17 mmol).

^1^H NMR (300 MHz, CDCl_3_) δ 12.60 (s, 1H), 7.77 – 7.52 (m, 6H), 7.40 (qd, *J* = 4.0, 1.6 Hz, 7H), 6.99 (d, *J* = 8.3 Hz, 1H), 6.43 (d, *J* = 2.0 Hz, 1H), 6.33 (d, *J* = 2.1 Hz, 1H), 4.03 – 3.87 (m, 2H), 3.57 – 3.36 (m, 2H), 1.95 – 1.73 (m, 4H). The obtained spectral data are consistent with the literature reports [5].

***Step H***

*full name: 3-(4-O-chlorobutyl) quercetin or alternatively:*

*3-(4-chlorobutoxy)-2-(3,4-dihydroxyphenyl)-5,7-dihydroxy-4H-chromen-4-one*

3-(4-*O*-chlorobutyl)-3′,4′-*O*-diphenylmethane quercetin (0.65 g, 1.17 mmol) was dissolved in a mixture of ethanol : tetrahydrofuran 1:1 (60 ml). The mixture was deoxygenated with the use of nitrogen and catalytic amount of palladium on carbon 10 wt % was added. The reaction was stirred overnight under hydrogen atmosphere at RT. Then, the reaction mixture was filtered on Celite^®^ and washed with ethanol (100 ml). The filtrate was concentrated under reduced pressure to afford the crude product, which was purified by column chromatography using a DCM:MeOH gradient (from 99:1 to 9:1). 3-(4-O-chlorobutyl)quercetin was obtained as a green-yellow solid in 83% yield (0.38 g, 0.97 mmol).

^1^H NMR (300 MHz, DMSO-*d*_6_) δ 12.71 (s, 1H), 9.82 (s, 3H), 7.51 (d, *J* = 2.2 Hz, 1H), 7.43 (dd, *J* = 8.4, 2.2 Hz, 1H), 6.89 (d, *J* = 8.4 Hz, 1H), 6.40 (d, *J* = 2.0 Hz, 1H), 6.19 (d, *J* = 2.0 Hz, 1H), 3.94 (t, *J* = 6.0 Hz, 2H), 3.66 (t, *J* = 6.3 Hz, 2H), 1.95 – 1.69 (m, 4H).

^13^C NMR (75 MHz, DMSO) δ 177.9, 164.3, 161.3, 156.4, 148.7, 145.2, 136.6, 120.9, 120.7, 115.6, 115.5, 104.1, 93.6, 71.1, 45.6, 45.1, 40.4, 40.1, 39.8, 39.5, 39.2, 39.0, 38.7, 28.7, 26.8, 9.7.

***Step B***

*full name: 3-(4-O-chlorobutyl)-3′,4′,5,7-tetraacetyl quercetin or alternatively:*

*3-(4-chlorobutoxy)-2-(3,4-diacetoxyphenyl)-4-oxo-4H-chromene-5,7-diyl diacetate*

3-(4-O-chlorobutyl) quercetin (0.380 g, 0.97 mmol) was dissolved in 10 ml DCM and Et_3_N (1.29 ml, 9.3 mmol) was added. The solution was cooled to 0°C and acetic anhydride (0.73 ml, 7.7 mmol) was added dropwise. The reaction mixture was allowed to warm up to RT and it was stirred until starting material disappeared completely (monitored by TLC, with petroleum ether : acetone 7:3 as eluent). After finishing, the reaction mixture was diluted by DCM and extracted 3 times with 1N HCl. Organic phase was dried over anhydrous MgSO_4_. After solvent evaporation, the crude product was recrystallized from DCM/petroleum ether mixture. Isolated yield: 70% (0.38 g, 0.68 mmol).

^1^H NMR (300 MHz, CDCl_3_) δ 8.02 – 7.89 (m, 2H), 7.41 – 7.27 (m, 2H), 6.82 (d, *J* = 2.2 Hz, 1H), 4.00 (t, *J* = 5.7 Hz, 2H), 3.56 (t, *J* = 6.1 Hz, 2H), 2.45 (s, 3H), 2.36-2.29 (m, 9H), 1.87 (dtd, *J* = 10.5, 5.2, 2.1 Hz, 4H).

^13^C NMR (75 MHz, CDCl_3_) δ 173.2, 169.5, 168.2, 168.1, 168.0, 156.6, 154.0, 153.2, 150.3, 144.1, 142.2, 141.2, 129.0, 126.9, 124.1, 123.8, 115.3, 113.5, 109.0, 72.1, 44.9, 29.2, 27.4, 21.3, 21.2, 20.8, 20.8.

***Step F***

*full name: 3-(4-O-iodobutyl)-3′,4′,5,7-tetraacetyl quercetin or alternatively:*

*4-(5,7-diacetoxy-3-(4-iodobutoxy)-4-oxo-4H-chromen-2-yl)-1,2-phenylene diacetate*

3-(4-O-chlorobutyl)-3′,4′,5,7-tetraacetyl quercetin (76 mg, 0.14 mmol) and NaI (0.41 g, 2.7 mmol) were dissolved in acetone (2 ml) and heat at reflux overnight. Next day, small amount of solution was taken, evaporated under reduced pressure and measured by ^1^H NMR to calculate the reaction conversion (Rf of substrate and product are the same). If there was no substrate traces on ^1^H NMR spectrum, the reaction mixture was cooled, diluted with 50 ml of ethyl acetate, filtered through paper filter directly into the separating funnel and washed 3 times with 25 ml of water. Organic layer was dried with anhydrous MgSO_4_. The crude product (81 mg, 0.12 mmol, 91% of isolated yield) was pure enough to be used in the next step without further purification.

^1^H NMR (300 MHz, CDCl_3_) δ 8.00 – 7.86 (m, 2H), 7.38 – 7.26 (m, 2H), 6.81 (d, J = 2.2 Hz, 1H), 3.97 (t, J = 6.1 Hz, 2H), 3.20 (t, J = 6.8 Hz, 2H), 2.44 (s, 3H), 2.39 – 2.23 (m, 9H), 1.92 (dqd, J = 9.1, 6.8, 1.8 Hz, 2H), 1.85 – 1.69 (m, 2H).

^13^C NMR (75 MHz, CDCl_3_) δ 173.1, 169.4, 168.0, 168.0, 167.9, 156.5, 153.9, 153.0, 150.2, 144.0, 142.1, 141.0, 128.8, 126.8, 123.9, 123.7, 115.1, 113.4, 108.9, 71.6, 30.8, 29.9, 21.2, 21.2, 20.7, 6.8.

***Step G***

*full name:3-(4-O-azidobutyl)-3′,4′,5,7-tetraacetyl quercetin (*3-N_3_Bu-Q(OAc)_4_ )

*or alternatively:*

*3-(4-azidobutoxy)-2-(3,4-diacetoxyphenyl)-4-oxo-4H-chromene-5,7-diyl diacetate*

3-(4-O-iodobutyl)-3′,4′,5,7-tetraacetyl quercetin (81 mg, 0.12 mmol) was placed in round-bottom flask equipped with dry molecular sieves and closed with septum. The atmosphere in the flask was exchanged for nitrogen by three vacuum-nitrogen cycles and 2 ml of dry DMF was added. Then, NaN_3_ (12 mg, 0.19 mmol) in 3 ml of dry DMF was slowly added dropwise. The reaction was monitored by taking 50 μl aliquots every 20 min and recording the ¹H NMR spectrum in CDCl₃. After 1 h, the reaction was quenched. The reaction mixture was diluted with 100 ml of ethyl acetate and washed 3 times with 50 ml of water. Organic phase was dried over anhydrous MgSO_4_. The crude product was purified by column chromatography with elucidation by petroleum ether:acetone (8:2) mixture to give 3-(4-O-azidobutyl)-3′,4′,5,7-tetraacetyl quercetin (3-N_3_Bu-Q(OAc)_4_ ) as light yellow solid with isolated yield 54% (38 mg, 67 µmol).

^1^H NMR (300 MHz, CDCl_3_) δ 8.01 – 7.90 (m, 2H), 7.41 – 7.28 (m, 2H), 6.83 (d, *J* = 2.2 Hz, 1H), 3.99 (t, *J* = 6.0 Hz, 2H), 3.29 (t, *J* = 6.5 Hz, 2H), 2.46 (s, 3H), 2.41 – 2.29 (m, 9H), 1.82 – 1.66 (m, 4H).

^13^C NMR (75 MHz, CDCl_3_) δ 173.2, 169.5, 168.2, 168.1, 168.0, 156.7, 154.0, 153.2, 150.4, 144.1, 142.2, 141.2, 129.0, 126.9, 124.1, 123.8, 115.3, 113.5, 109.0, 72.3, 51.2, 27.3, 25.6, 21.3, 21.2, 20.8, 20.8.


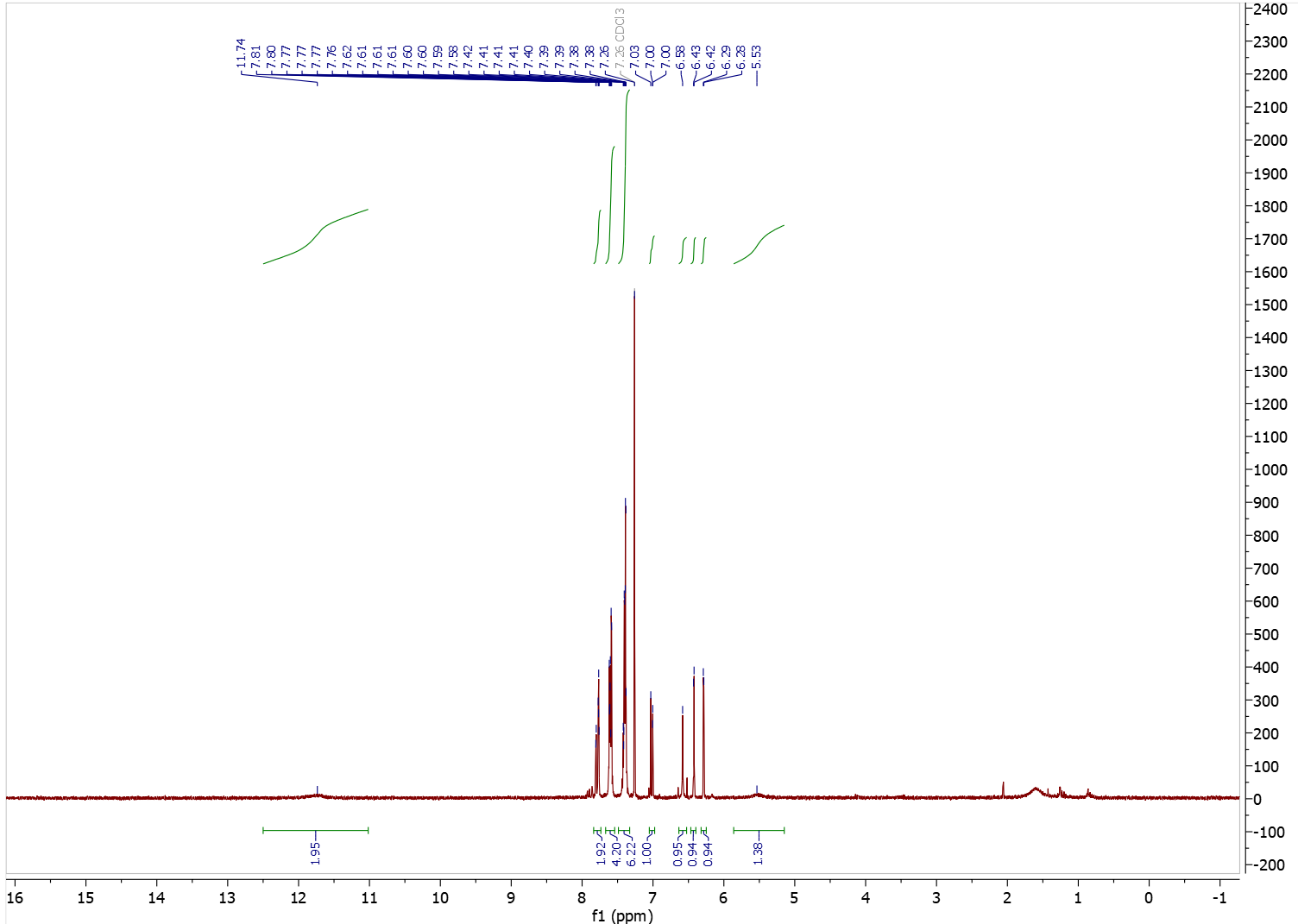

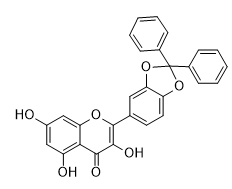


traces of water

**Figure S5.** ^1^H NMR (300 MHz, CDCl_3_) spectrum of 2-(2,2-diphenylbenzo[d][1,3]dioxol-5-yl)-3,5,7-trihydroxy-4H-chromen-4-one.


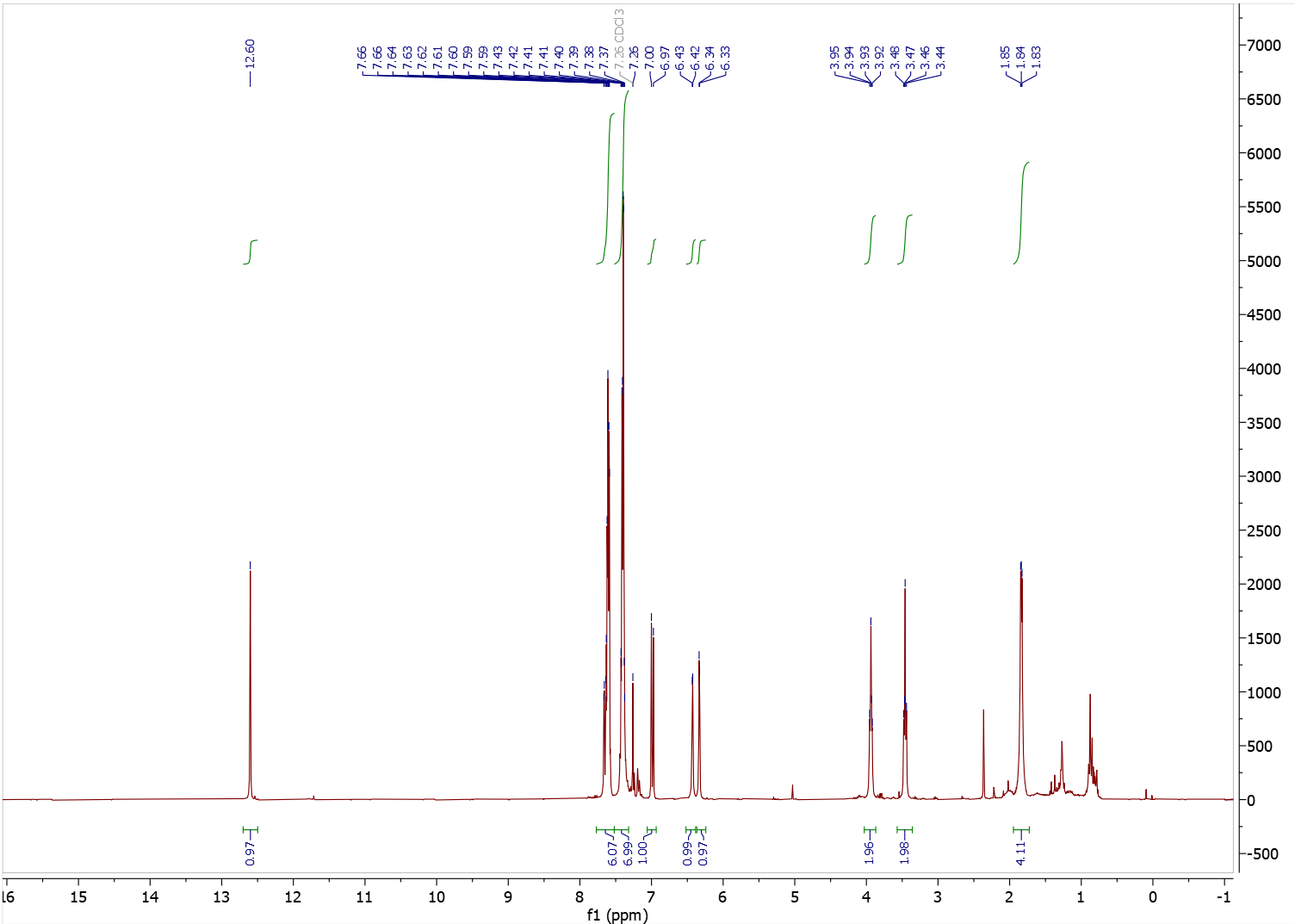

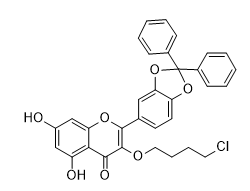


traces of n-hexane

**Figure S6.** ^1^H NMR (300 MHz, CDCl_3_) spectrum of 3-(4-chlorobutoxy)-2-(2,2-diphenylbenzo[d][1,3]dioxol-5-yl)-5,7-dihydroxy-4H-chromen-4-one.


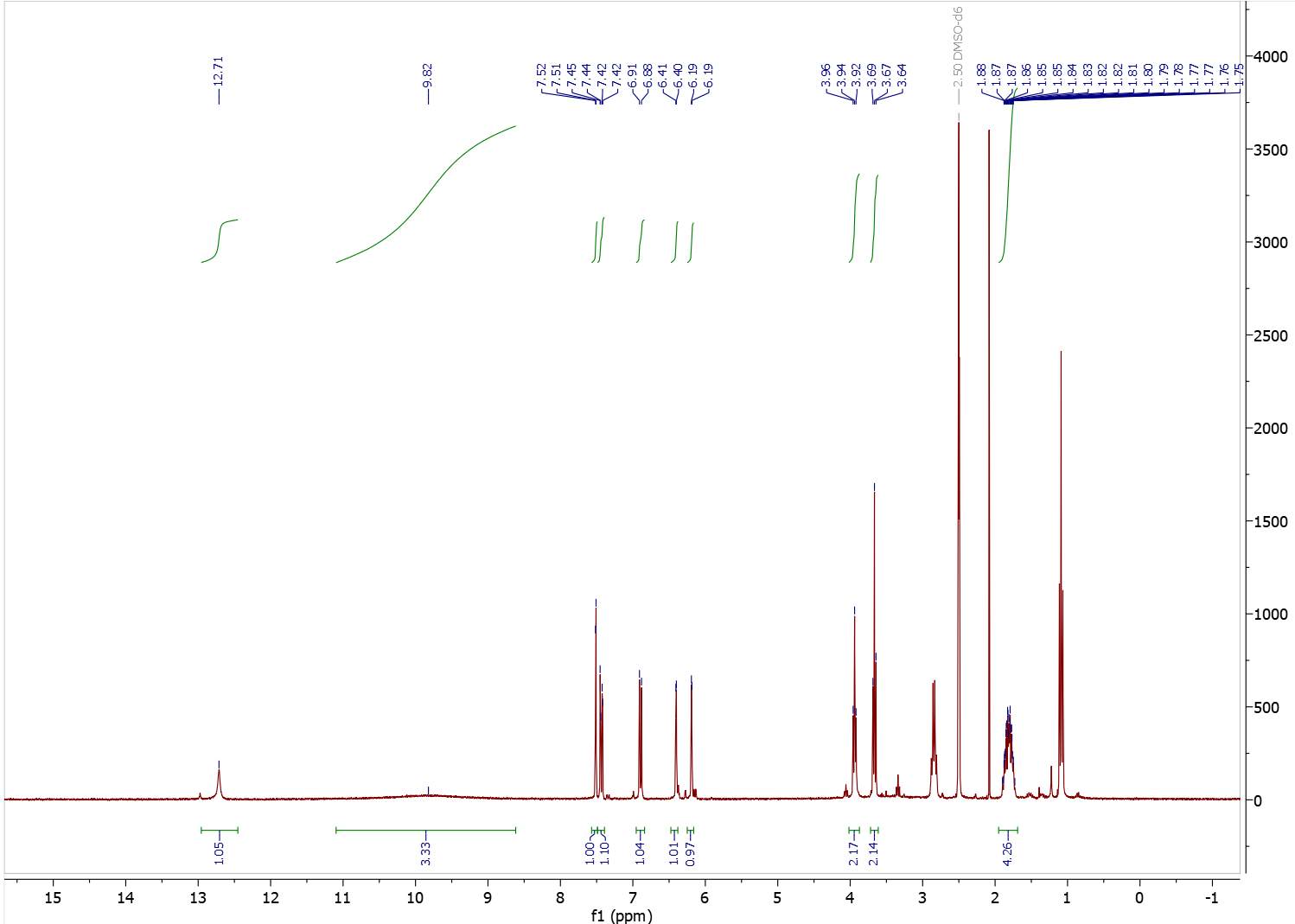

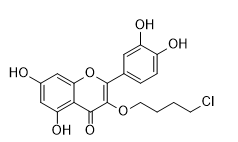


**Figure S7.** ^1^H NMR (300 MHz, DMSO-*d*_6_) spectrum of 3-(4-chlorobutoxy)-2-(3,4-dihydroxyphenyl)-5,7-dihydroxy-4H-chromen-4-one. Additional minor peaks at 2.8 (q, 2H) and 1.1 ppm (t, 3H) correspond to trace ethyl-containing impurities, and a small singlet at 2.09 ppm corresponds to acetone (solvent residue).


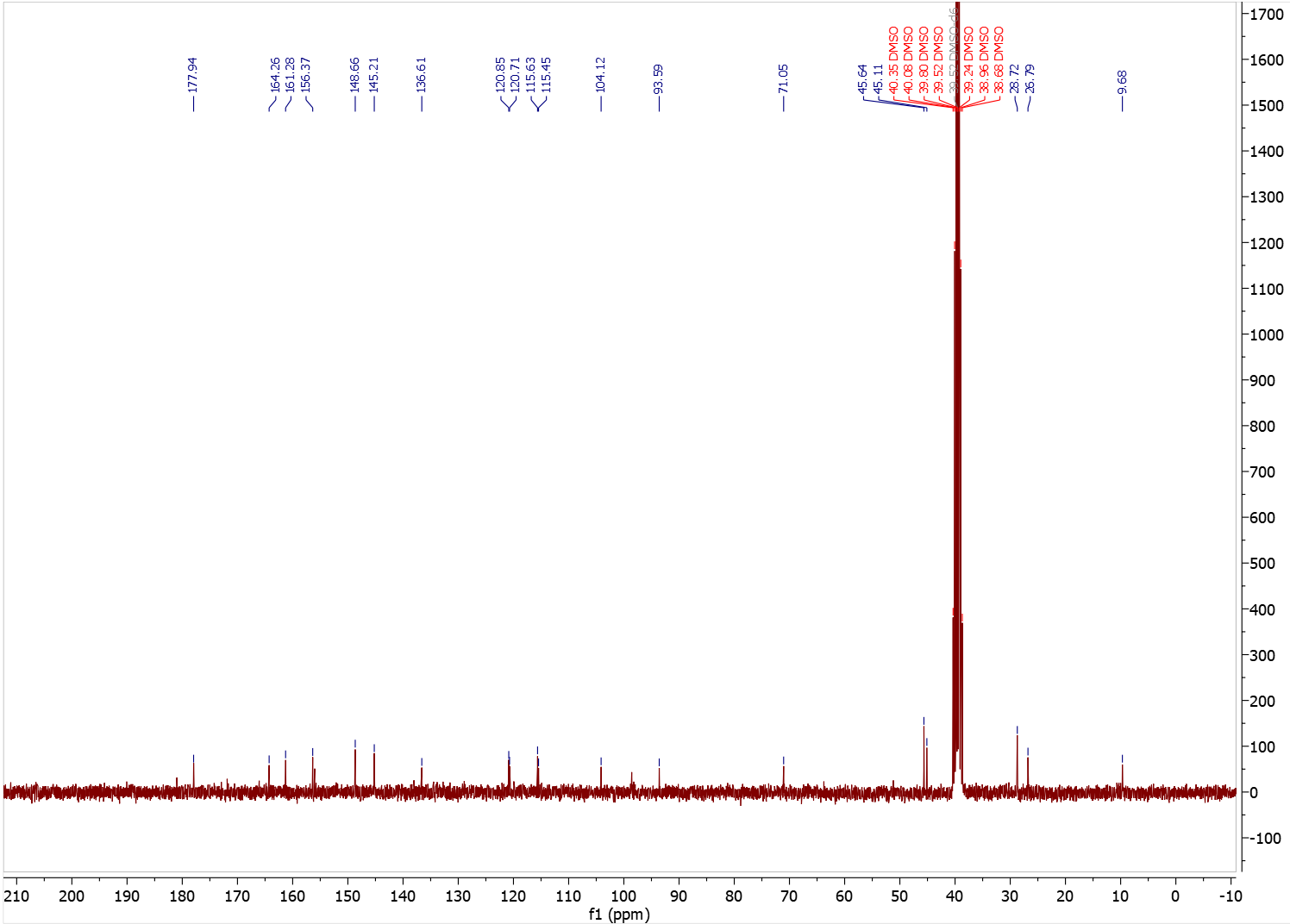

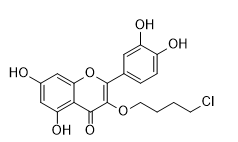


**Figure S8.** ^13^C NMR (75 MHz, DMSO-*d*_6_) spectrum of 3-(4-chlorobutoxy)-2-(3,4-dihydroxyphenyl)-5,7-dihydroxy-4H-chromen-4-one.


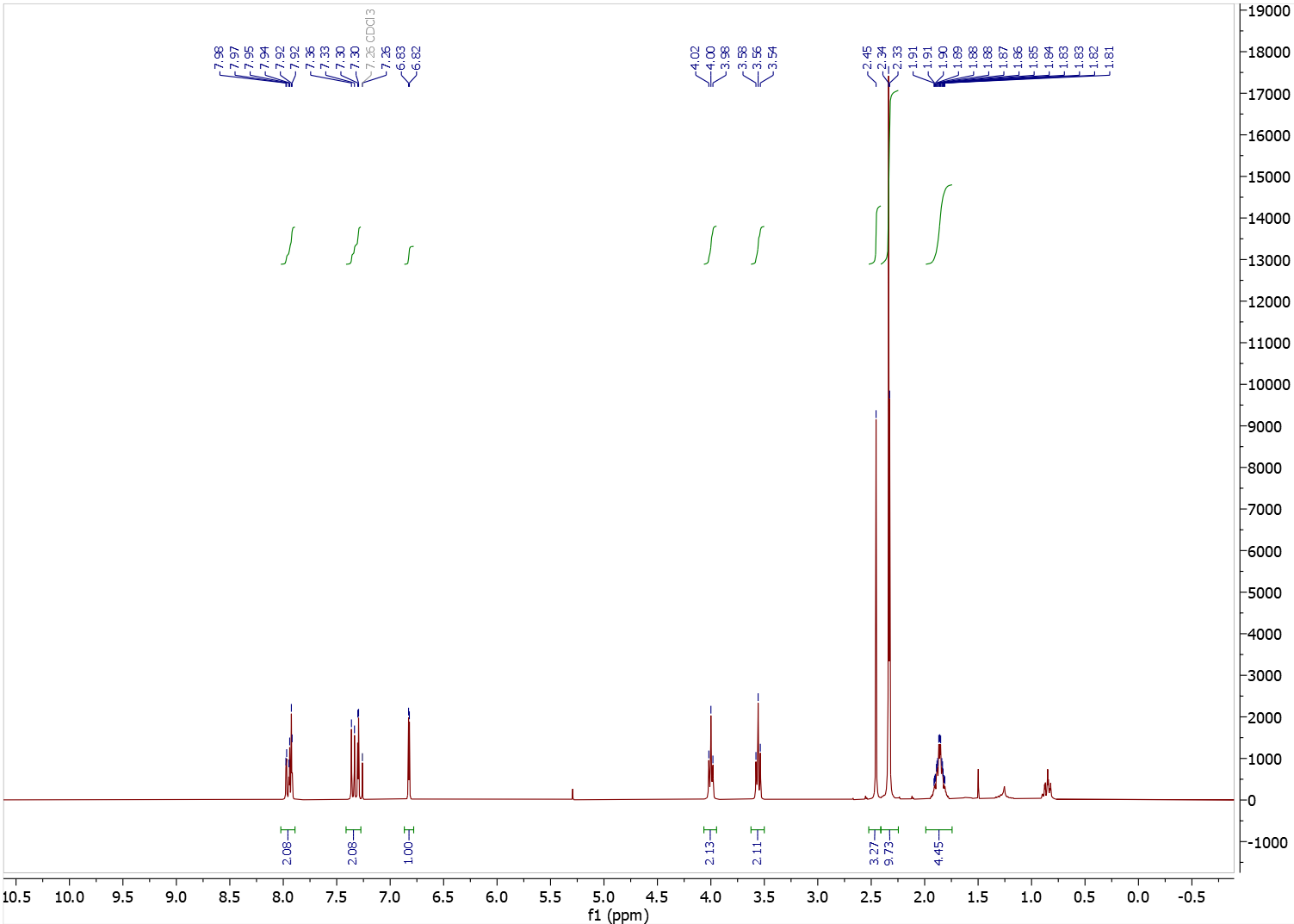

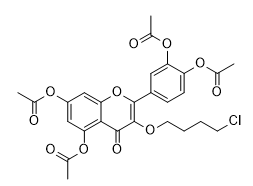


**Figure S9.** ^1^H NMR (300 MHz, CDCl_3_) spectrum of 3-(4-chlorobutoxy)-2-(3,4-diacetoxyphenyl)-4-oxo-4H-chromene-5,7-diyl diacetate.


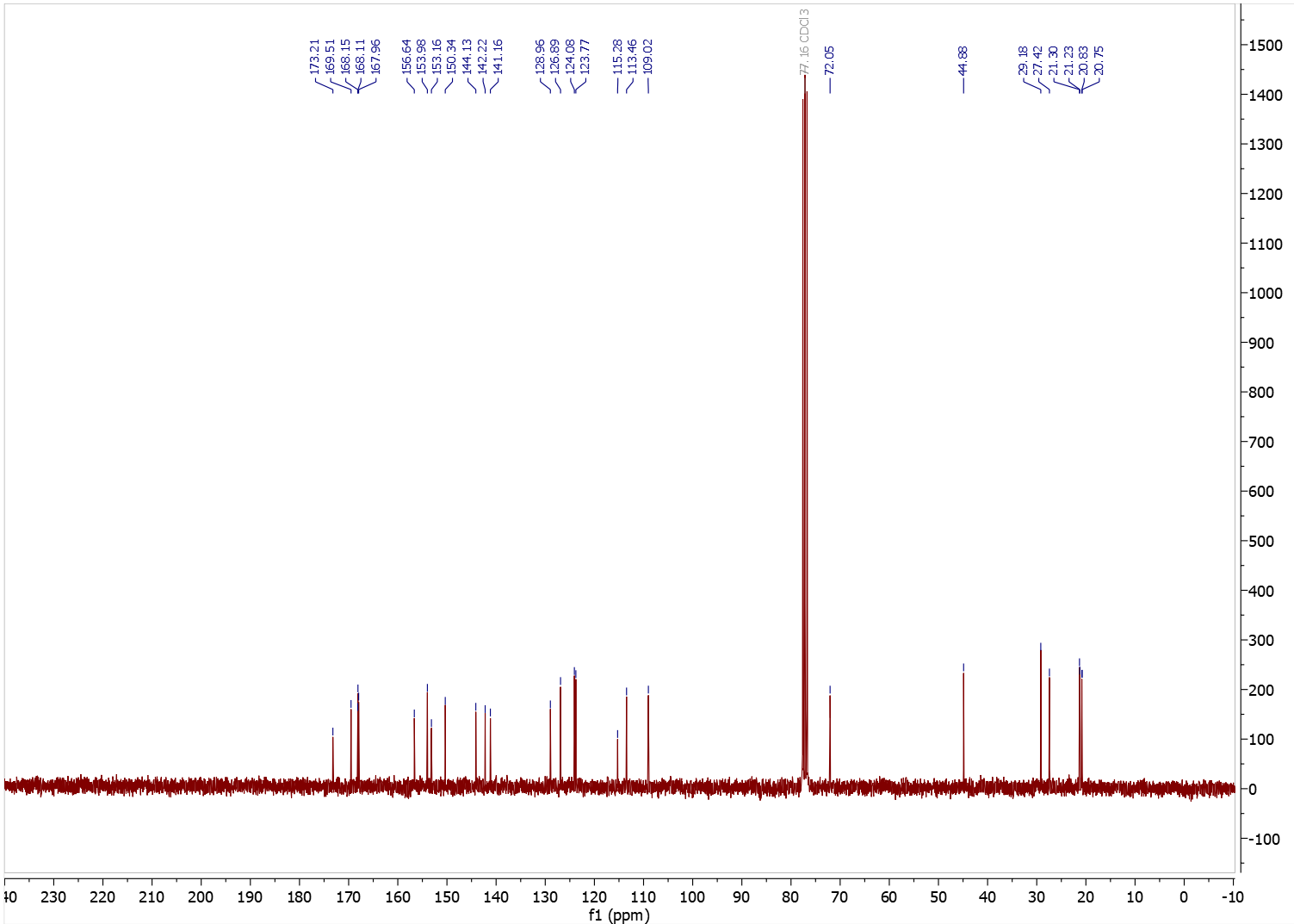

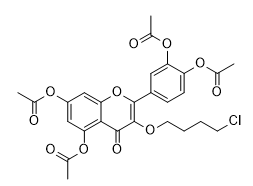


**Figure S10.** ^13^C NMR (75 MHz, CDCl_3_) spectrum of 3-(4-chlorobutoxy)-2-(3,4-diacetoxyphenyl)-4-oxo-4H-chromene-5,7-diyl diacetate.

**Figure S11.** ^1^H NMR (300 MHz, CDCl_3_) spectrum of 4-(5,7-diacetoxy-3-(4-iodobutoxy)-4-oxo-4H-chromen-2-yl)-1,2-phenylene diacetate.


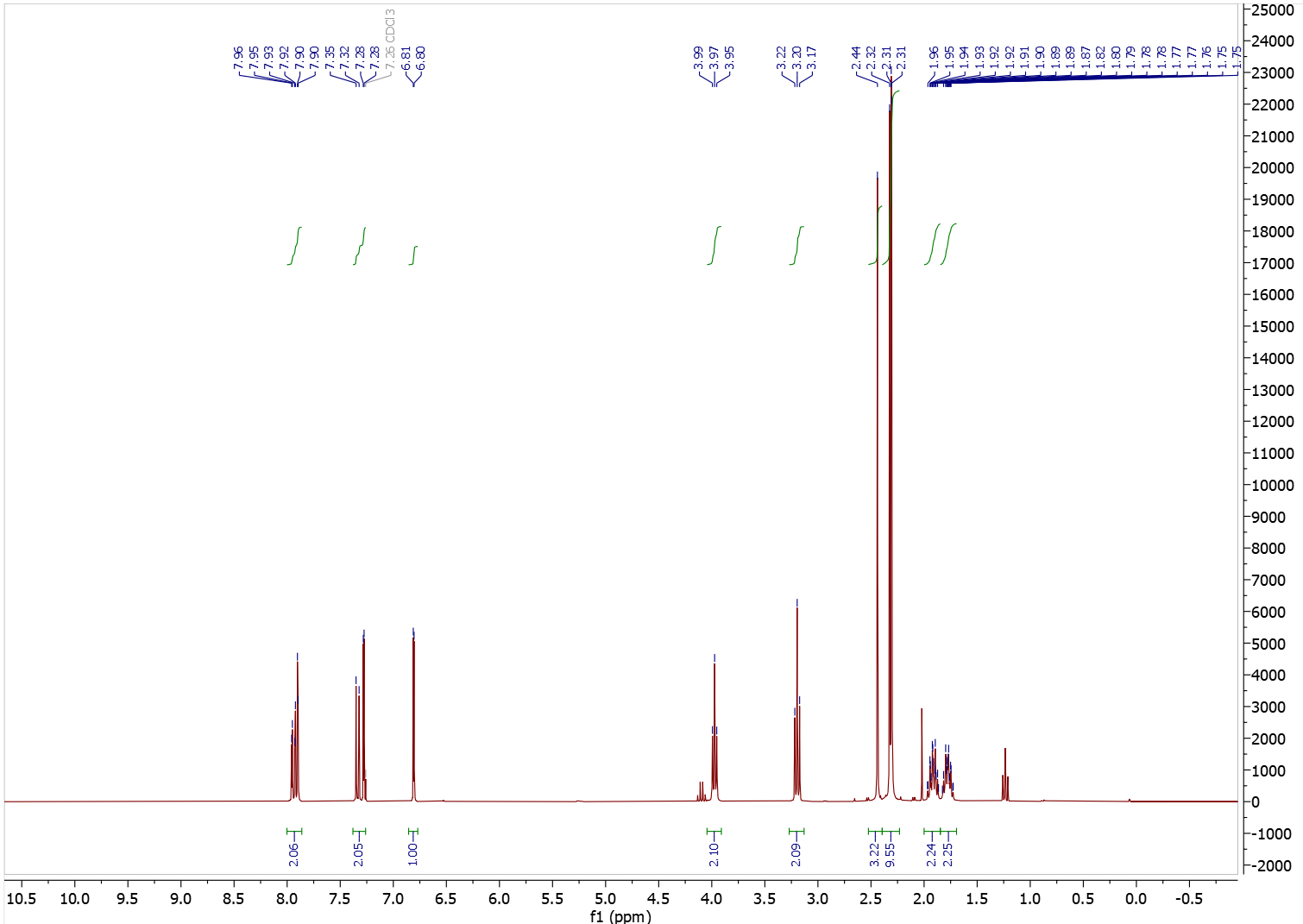

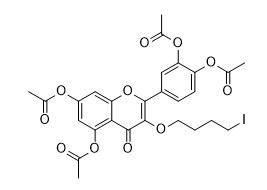


traces of ethyl acetate

traces of ethyl acetate

traces of ethyl acetate


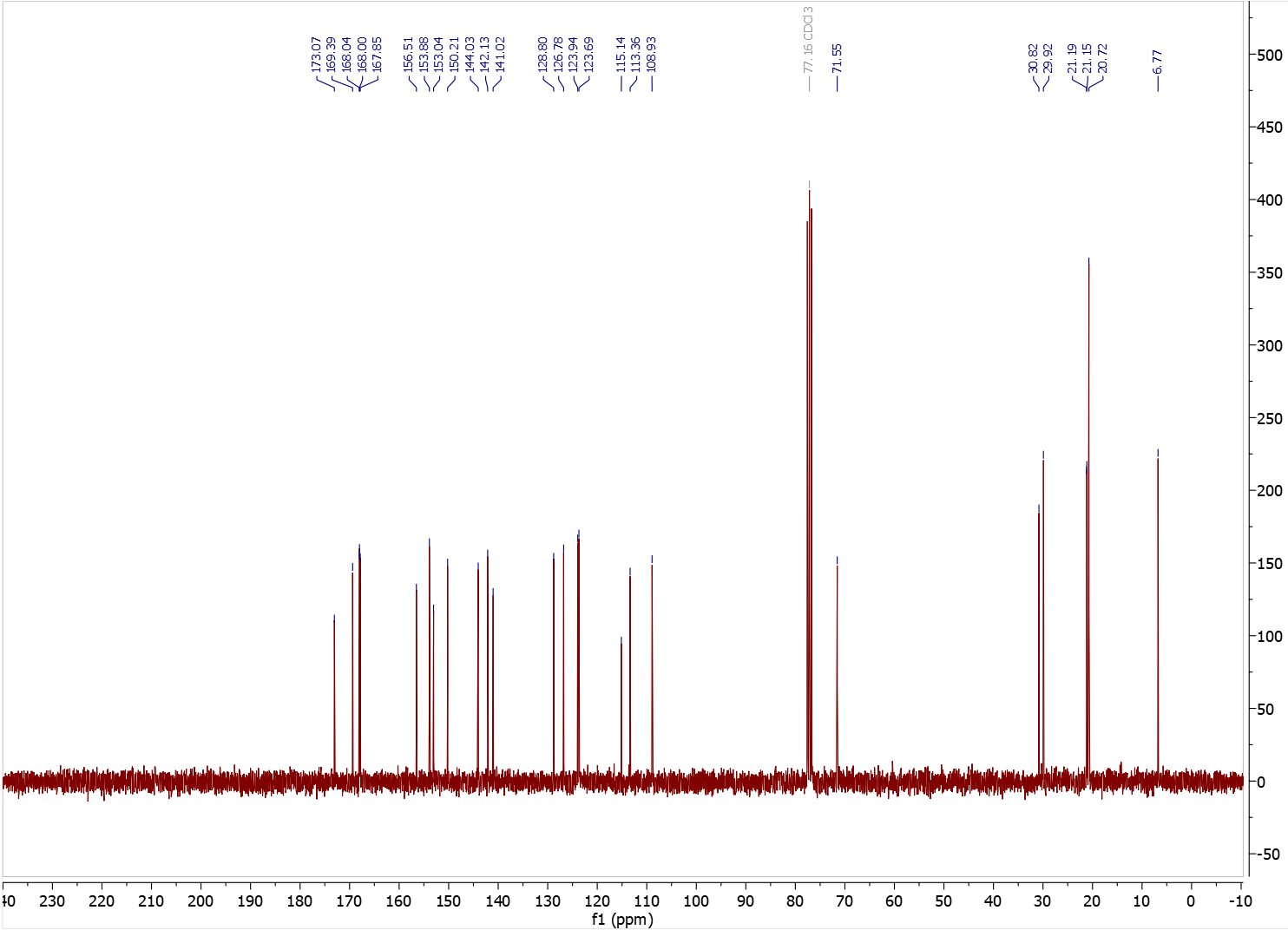

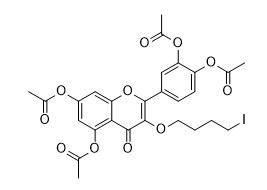


**Figure S12.** ^13^C NMR (75 MHz, CDCl_3_) spectrum of 4-(5,7-diacetoxy-3-(4-iodobutoxy)-4-oxo-4H-chromen-2-yl)-1,2-phenylene diacetate.


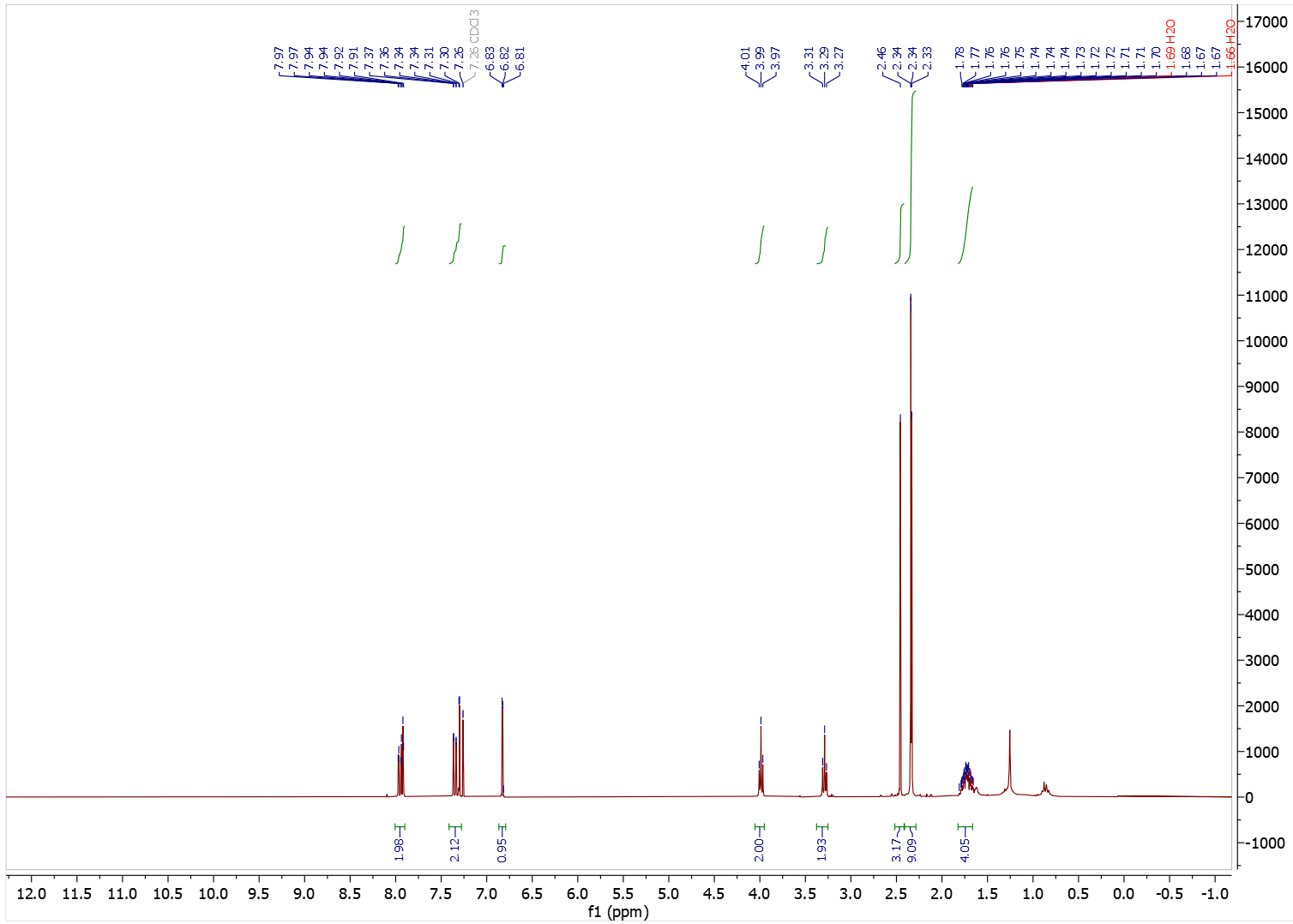

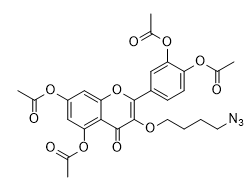


traces of petroleum ether

**Figure S13.** ^1^H NMR (300 MHz, CDCl_3_) spectrum of 3-(4-azidobutoxy)-2-(3,4-diacetoxyphenyl)-4-oxo-4H-chromene-5,7-diyl diacetate (**3-N_3_Bu-Q(OAc)_4_**).


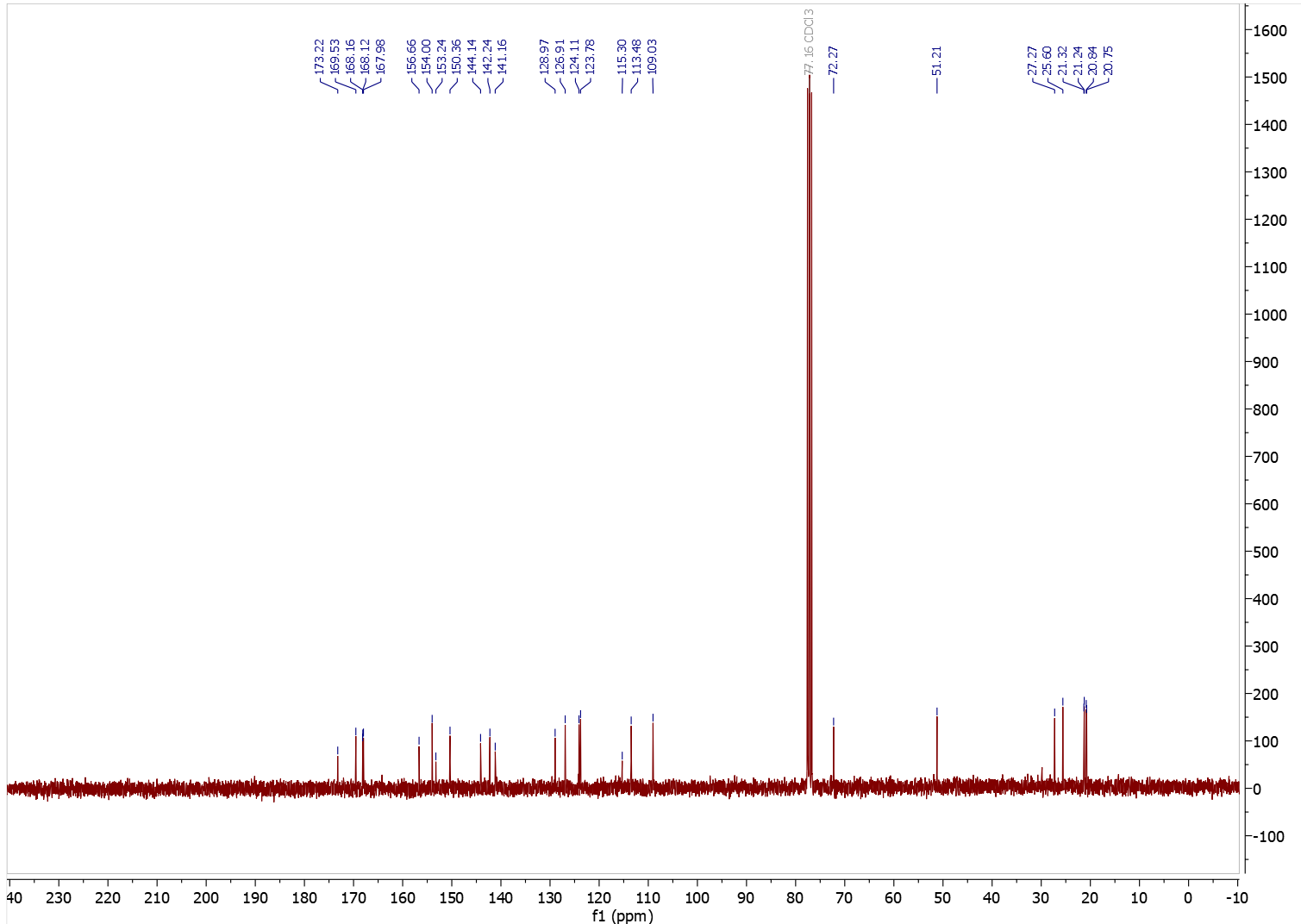

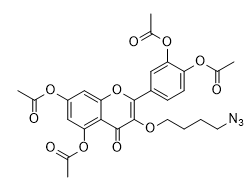


**Figure S14.** ^13^C NMR (75 MHz, CDCl_3_) spectrum of 3-(4-azidobutoxy)-2-(3,4-diacetoxyphenyl)-4-oxo-4H-chromene-5,7-diyl diacetate (**3-N_3_Bu-Q(OAc)_4_**).

**3.2. Compound 5-N_3_Bu-Q(OAc)_4_**

*Full name: 5-(4-azidobutoxy)-2-(3,4-diacetoxyphenyl)-4-oxo-4H-chromene-3,7-diyl diacetate*

***Step I***

*full name: 3,3′,4′,7-tetrabenzyl quercetin, alternative name:*

*3,7-bis(benzyloxy)-2-(3,4-bis(benzyloxy)phenyl)-5-hydroxy-4H-chromen-4-one*

Quercetin (1.82 g, 6.01 mmol) and K_2_CO_3_ (2.90 g, 21.0 mmol) were dissolved in DMF (60 ml), cooled to 0°C and BnBr (2.50 ml, 21.0 mmol) was added dropwise with vigorous stirring. After 2 h, the reaction mixture was allowed to warm to RT and the stirring was continued overnight. Then, after TLC confirmation that all the substrate had reacted, the mixture was diluted with 150 ml of ethyl acetate and washed with 1N HCl (3x100 ml). Organic phase was dried with anhydrous MgSO_4_ and the solvent was evaporated under reduced pressure. Purification by column chromatography using petroleum ether:DCM:ethyl acetate (6:2:1) mixture as eluent led to obtain 3,3′,4′,7-tetrabenzyl quercetin as yellow solid with isolated yield: 68% (2.69 g, 4.06 mmol).

^1^H NMR (300 MHz, CDCl_3_) δ 12.67 (s, 1H), 7.71 (d, *J* = 2.1 Hz, 1H), 7.56 (dd, *J* = 8.6, 2.1 Hz, 1H), 7.50 – 7.27 (m, 16H), 7.26 – 7.21 (m, 3H), 6.97 (d, *J* = 8.6 Hz, 1H), 6.51 – 6.41 (m, 2H), 5.25 (s, 2H), 5.13 (s, 2H), 5.05 (s, 2H), 5.00 (s, 2H). The obtained spectral data are consistent with the literature reports[5].

***Step C***

*full name: 3,3′,4′,7-tetrabenzyl-5-(4-O-chlorobutyl) quercetin, alternative name:*

*3,7-bis(benzyloxy)-2-(3,4-bis(benzyloxy)phenyl)-5-(4-chlorobutoxy)-4H-chromen-4-one*

3,3′,4′,7-tetrabenzyl quercetin (2.69 g, 4.06 mmol) and K_2_CO_3_ (0.62 g, 4.47 mmol) were dissolved in DMF (15 ml) and 1-bromo-4-chlorobutane (1.05 g, 6.10 mmol) was added. The reaction mixture was stirred at RT overnight. After confirming by TLC that all substrate had reacted, the reaction mixture was diluted with ethyl acetate (150 ml), transfer into the separating funnel and washed 3 times with 100 ml of 1N HCl. Organic layer was dried with anhydrous MgSO_4_. The solvent was evaporated and crude product was recrystallized from ethyl acetate/hexane mixture. The obtained product was slightly yellow solid with isolated yield: 87% (2.66 g, 3.53 mmol).

^1^H NMR (300 MHz, CDCl_3_) δ 7.72 (d, *J* = 2.1 Hz, 1H), 7.59 – 7.28 (m, 18H), 7.25 – 7.17 (m, 3H), 6.95 (d, *J* = 8.6 Hz, 1H), 6.52 (d, *J* = 2.2 Hz, 1H), 6.41 (d, *J* = 2.3 Hz, 1H), 5.23 (s, 2H), 5.13 (s, 2H), 5.07 (s, 2H), 4.95 (s, 2H), 4.20 – 4.03 (m, 2H), 3.77 – 3.64 (m, 2H), 2.12 (m, 4H). The obtained spectral data are consistent with the literature reports [5].

***Step H***

*full name: 5-(4-O-chlorobutyl) quercetin, alternative name:*

*5-(4-chlorobutoxy)-2-(3,4-dihydroxyphenyl)-3,7-dihydroxy-4H-chromen-4-one*

3,3′,4′,7-tetrabenzyl-5-(4-O-chlorobutyl) quercetin (2.66 g, 3.53 mmol) was dissolved in a mixture of ethanol : tetrahydrofuran 1:1 (150 ml). The mixture was deoxygenated with the use of nitrogen and catalytic amount of palladium on carbon 10 wt % was added. The reaction was stirred at RT under hydrogen atmosphere overnight. Then, the reaction mixture was filtered on Celite^®^ and washed with ethanol (250 ml). The filtrate was concentrated under reduced pressure to afford the crude product (1.37 g, 3.50 mmol, 99% yield), which was pure enough to be used in the next step without further purification.

^1^H NMR (300 MHz, Methanol-*d*_4_) δ 7.72 (d, *J* = 2.1 Hz, 1H), 7.62 (dd, *J* = 8.5, 2.0 Hz, 1H), 6.89 (d, *J* = 8.5 Hz, 1H), 6.49 (d, *J* = 1.8 Hz, 1H), 6.37 (s, 1H), 4.13 (s, 2H), 3.79 – 3.66 (m, 2H), 2.22 – 1.93 (m, 4H). The obtained spectral data are consistent with the literature reports [5].

***Step B***

*full name: 5-(4-O-chlorobutyl)-3′,4′,3,7-tetraacetyl quercetin, alternative name:*

*5-(4-chlorobutoxy)-2-(3,4-diacetoxyphenyl)-4-oxo-4H-chromene-3,7-diyl diacetate*

5-(4-O-Chlorobutyl)quercetin (1.27 g, 3.23 mmol) was dissolved in 50 ml of DCM, and triethylamine (2.15 ml, 15.5 mmol) was added. The solution was cooled to 0°C, followed by the dropwise addition of acetic anhydride (1.22 ml, 12.9 mmol). The reaction mixture was allowed to warm to RT and stirred until complete consumption of the starting material, as confirmed by TLC (cyclohexane:acetone, 1:1). Upon completion, the reaction mixture was diluted with 150 ml of DCM and washed three times with 100 ml portions of 1 N HCl. The organic layer was dried over anhydrous MgSO_4_, filtered, and concentrated under reduced pressure. The crude product was purified by column chromatography using a cyclohexane/acetone gradient (from 2:1 to 6:4) as the eluent, affording 5-(4-O-chlorobutyl)-3′,4′,3,7-tetraacetylquercetin as a white solid (1.09 g, 1.94 mmol, 60% yield).

^1^H NMR (300 MHz, CDCl_3_) δ 7.82 – 7.66 (m, 2H), 7.45 – 7.29 (m, 1H), 6.92 (d, *J* = 2.0 Hz, 1H), 6.56 (d, *J* = 2.1 Hz, 1H), 4.11 (d, *J* = 5.0 Hz, 2H), 3.75 – 3.58 (m, 2H), 2.42 – 2.27 (m, 12H), 2.08 (p, *J* = 3.2 Hz, 4H). ^13^C NMR (75 MHz, CDCl_3_) δ 170.5, 168.4, 168.2, 168.0, 167.9, 160.5, 157.9, 155.1, 152.4, 144.3, 142.3, 134.5, 128.2, 126.5, 124.0, 123.8, 112.4, 103.2, 102.0, 68.8, 45.0, 29.1, 26.3, 21.3, 20.8, 20.7.

***Step F***

*full name: 5-(4-O-iodobutyl)-3′,4′,3,7-tetraacetyl quercetin, alternative name:*

*4-(3,7-diacetoxy-5-(4-iodobutoxy)-4-oxo-4H-chromen-2-yl)-1,2-phenylene diacetate*

5-(4-O-Chlorobutyl)-3′,4′,3,7-tetraacetylquercetin (0.372 g, 0.66 mmol) and sodium iodide (1.99 g, 13.3 mmol) were dissolved in 10 ml of acetone and the mixture was heated to reflux overnight. The next day, a small aliquot of the reaction mixture was evaporated under reduced pressure and analyzed by ¹H NMR to determine reaction conversion, as the R_f_ values of the starting material and product were identical. If no traces of the starting material were observed in the NMR spectrum, the reaction mixture was cooled to RT, diluted with 100 ml of ethyl acetate, and filtered through paper directly into a separating funnel. The organic phase was washed three times with 50 ml portions of water, dried over anhydrous MgSO_4_, and concentrated. The crude product (0.394 g, 0.60 mmol) was obtained in 91% yield and was sufficiently pure to be used in the subsequent step without further purification.

^1^H NMR (300 MHz, CDCl_3_) δ 7.76 – 7.68 (m, 2H), 7.33 (dd, *J* = 8.4, 0.5 Hz, 1H), 6.92 (d, *J* = 2.1 Hz, 1H), 6.56 (d, *J* = 2.1 Hz, 1H), 4.11 – 4.05 (m, 2H), 3.31 (t, *J* = 6.4 Hz, 2H), 2.39 – 2.30 (m, 12H), 2.14 – 2.02 (m, 4H). ^13^C NMR (75 MHz, CDCl_3_) δ 168.4, 168.2, 168.0, 167.9, 160.5, 157.9, 155.1, 152.5, 144.3, 142.3, 134.6, 128.2, 126.5, 124.0, 123.9, 112.4, 103.2, 102.0, 68.5, 29.9, 29.7, 21.4, 20.8, 20.7, 7.2.

***Step G***

*full name: 5-(4-O-azidobutyl)-3′,4′,3,7-tetraacetyl quercetin (*5-N_3_Bu-Q(OAc)_4_ )*, alternative name: 5-(4-azidobutoxy)-2-(3,4-diacetoxyphenyl)-4-oxo-4H-chromene-3,7-diyl diacetate*

5-(4-O-Iodobutyl)-3′,4′,3,7-tetraacetylquercetin (0.394 g, 0.60 mmol) was placed in a round-bottom flask equipped with dried molecular sieves and sealed with a septum. The atmosphere was exchanged for nitrogen by performing three vacuum–nitrogen cycles. Then, 5 ml of dry DMF was added, followed by the slow dropwise addition of a solution of sodium azide (113 mg, 1.74 mmol) in 5 ml of dry DMF. The reaction was monitored by taking 50 μl aliquots every 20 min and recording the ¹H NMR spectrum in CDCl₃. After 1.5 h, the reaction was quenched. The mixture was diluted with 100 ml of ethyl acetate and washed three times with 50 ml portions of water. The organic layer was dried over anhydrous MgSO_4_, filtered, and concentrated under reduced pressure. Purification by column chromatography using a petroleum ether:acetone (8:2) mixture as eluent afforded 5-(4-O-azidobutyl)-3′,4′,3,7-tetraacetylquercetin (5-N_3_Bu-Q(OAc)_4_) as a light yellow solid (0.133 g, 0.23 mmol), corresponding to a 39% isolated yield. ^1^H NMR (300 MHz, CDCl_3_) δ 7.81 – 7.65 (m, 2H), 7.33 (dd, *J* = 8.3, 0.5 Hz, 1H), 6.92 (d, *J* = 2.0 Hz, 1H), 6.56 (d, *J* = 2.1 Hz, 1H), 4.09 (t, *J* = 5.9 Hz, 2H), 3.42 (t, *J* = 6.5 Hz, 2H), 2.43 – 2.25 (m, 12H), 2.08 – 1.82 (m, 4H). ^13^C NMR (75 MHz, CDCl_3_) δ 170.5, 168.4, 168.2, 168.0, 167.9, 160.4, 157.9, 155.0, 152.4, 144.2, 142.3, 134.6, 128.2, 126.4, 123.9, 123.8, 112.4, 103.2, 102.0, 69.0, 51.3, 26.2, 25.7, 21.3, 20.8, 20.7.


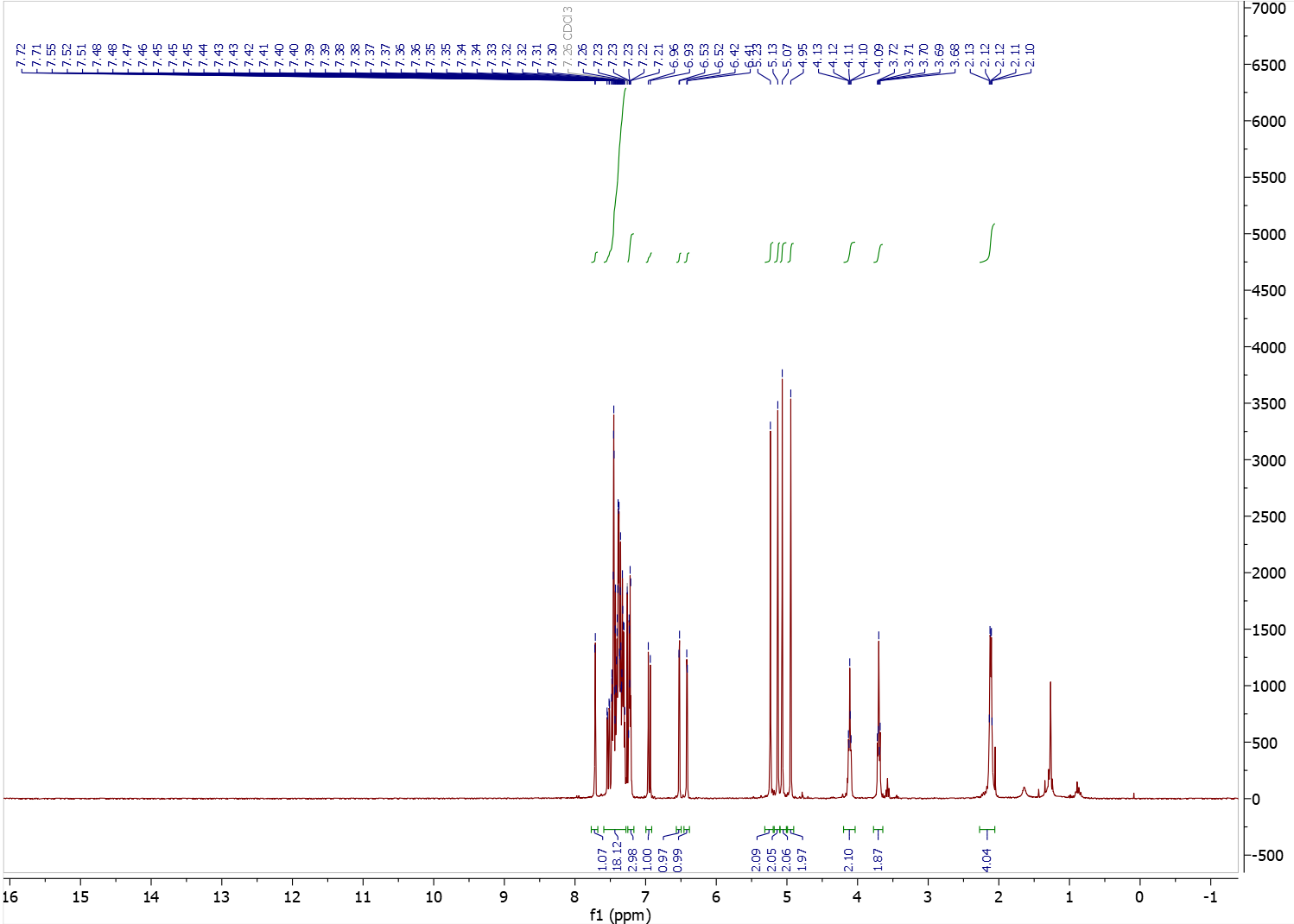

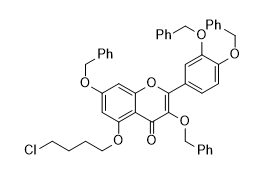


traces of water and n-hexane

**Figure S15.** ^1^H NMR (300 MHz, CDCl_3_) spectrum of 3,7-bis(benzyloxy)-2-(3,4-bis(benzyloxy)phenyl)-5-hydroxy-4H-chromen-4-one.


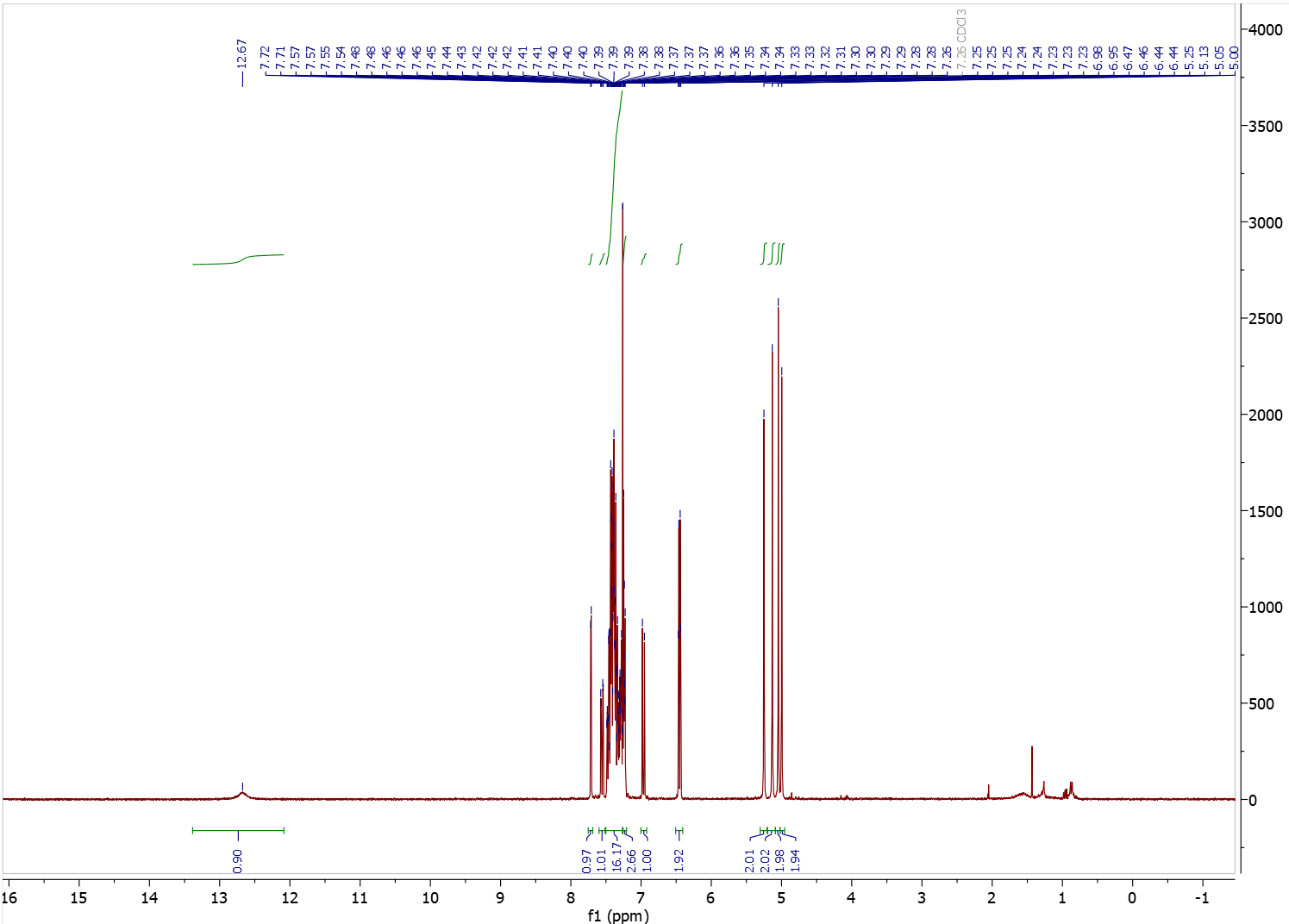

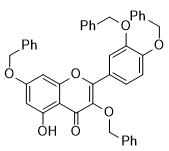


traces of water and petroleum ether

**Figure S16.** ^1^H NMR (300 MHz, CDCl_3_) spectrum of 3,7-bis(benzyloxy)-2-(3,4-bis(benzyloxy)phenyl)-5-(4-chlorobutoxy)-4H-chromen-4-one.


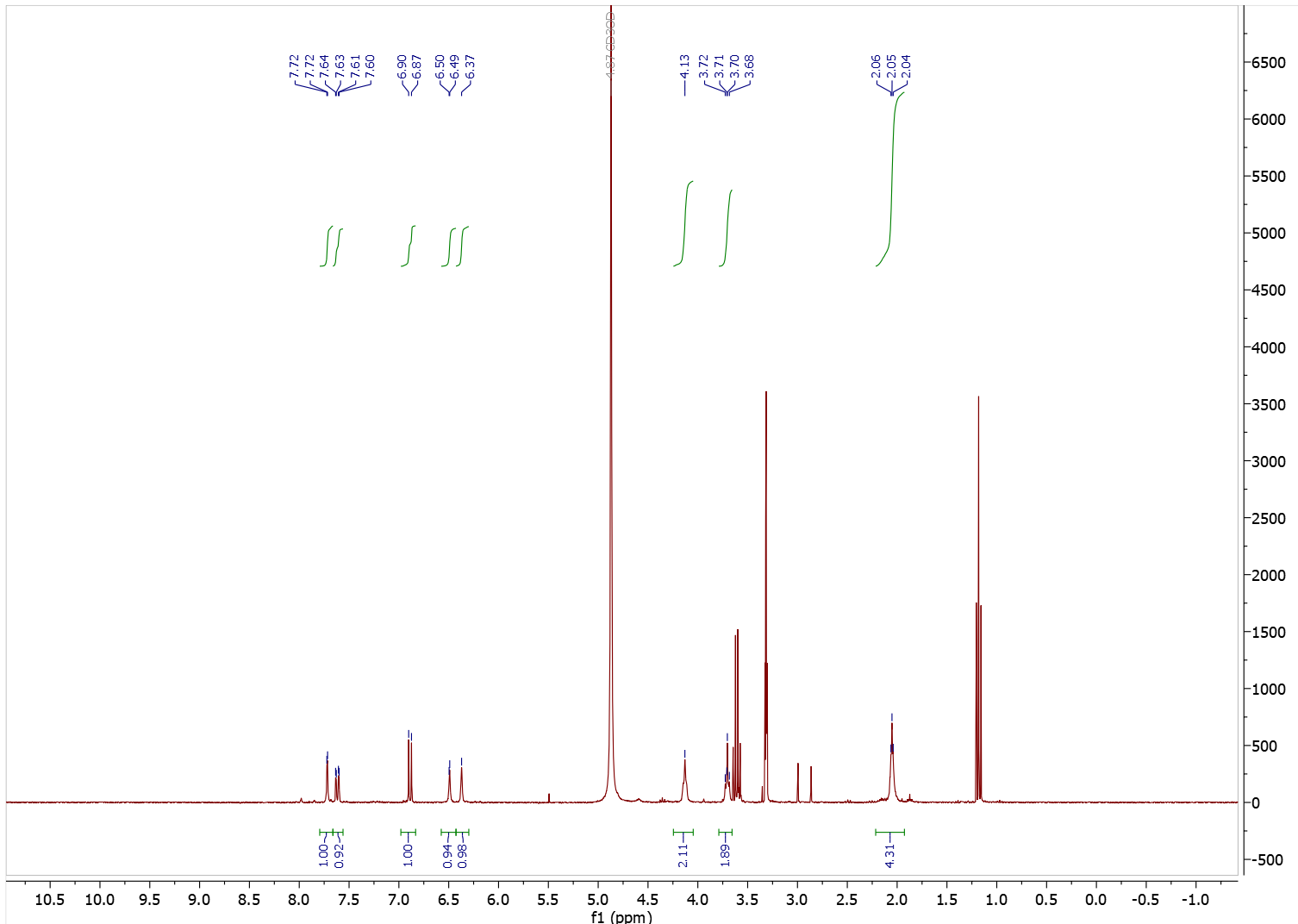

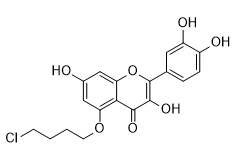


ethanol

ethanol

**Figure S17.** ^1^H NMR (300 MHz, CDCl_3_) spectrum of 5-(4-chlorobutoxy)-2-(3,4-dihydroxyphenyl)-3,7-dihydroxy-4H-chromen-4-one (crude).


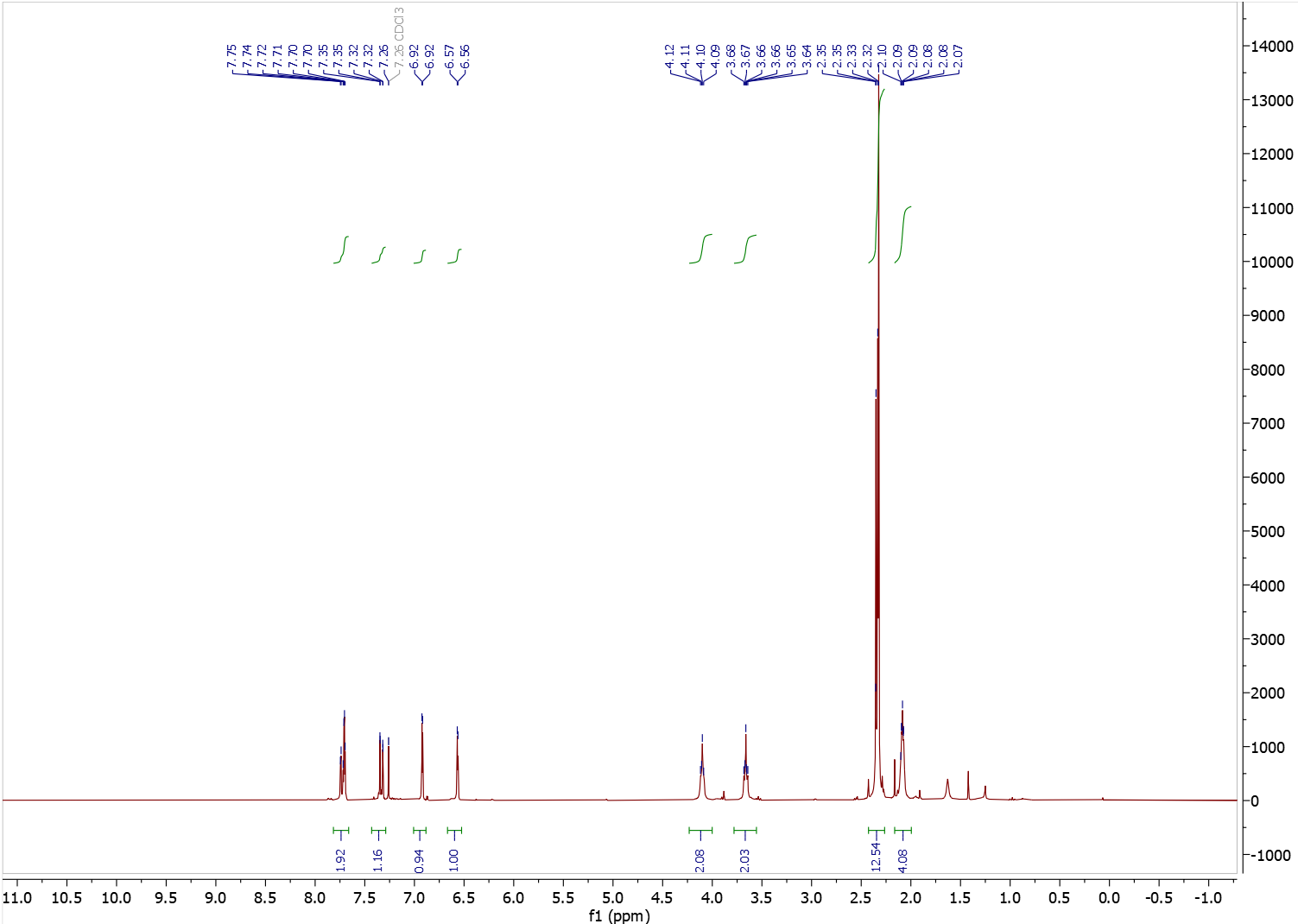

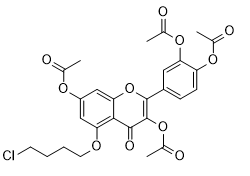


**Figure S18.** ^1^H NMR (300 MHz, CDCl_3_) spectrum of 5-(4-chlorobutoxy)-2-(3,4-diacetoxyphenyl)-4-oxo-4H-chromene-3,7-diyl diacetate.


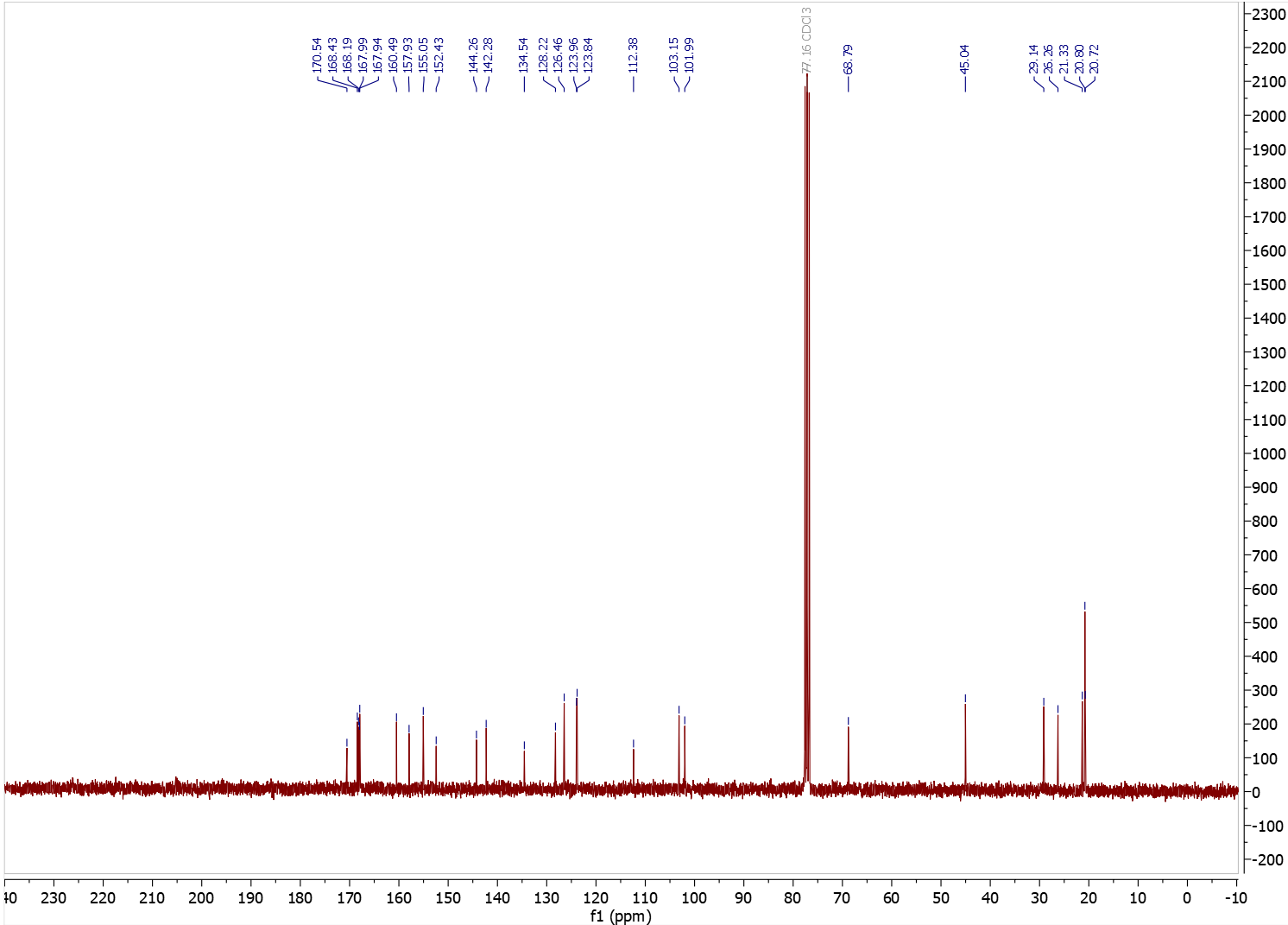

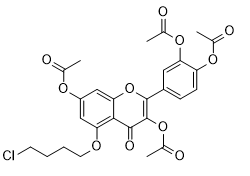


**Figure S19.** ^13^C NMR (75 MHz, CDCl_3_) spectrum of 5-(4-chlorobutoxy)-2-(3,4-diacetoxyphenyl)-4-oxo-4H-chromene-3,7-diyl diacetate.


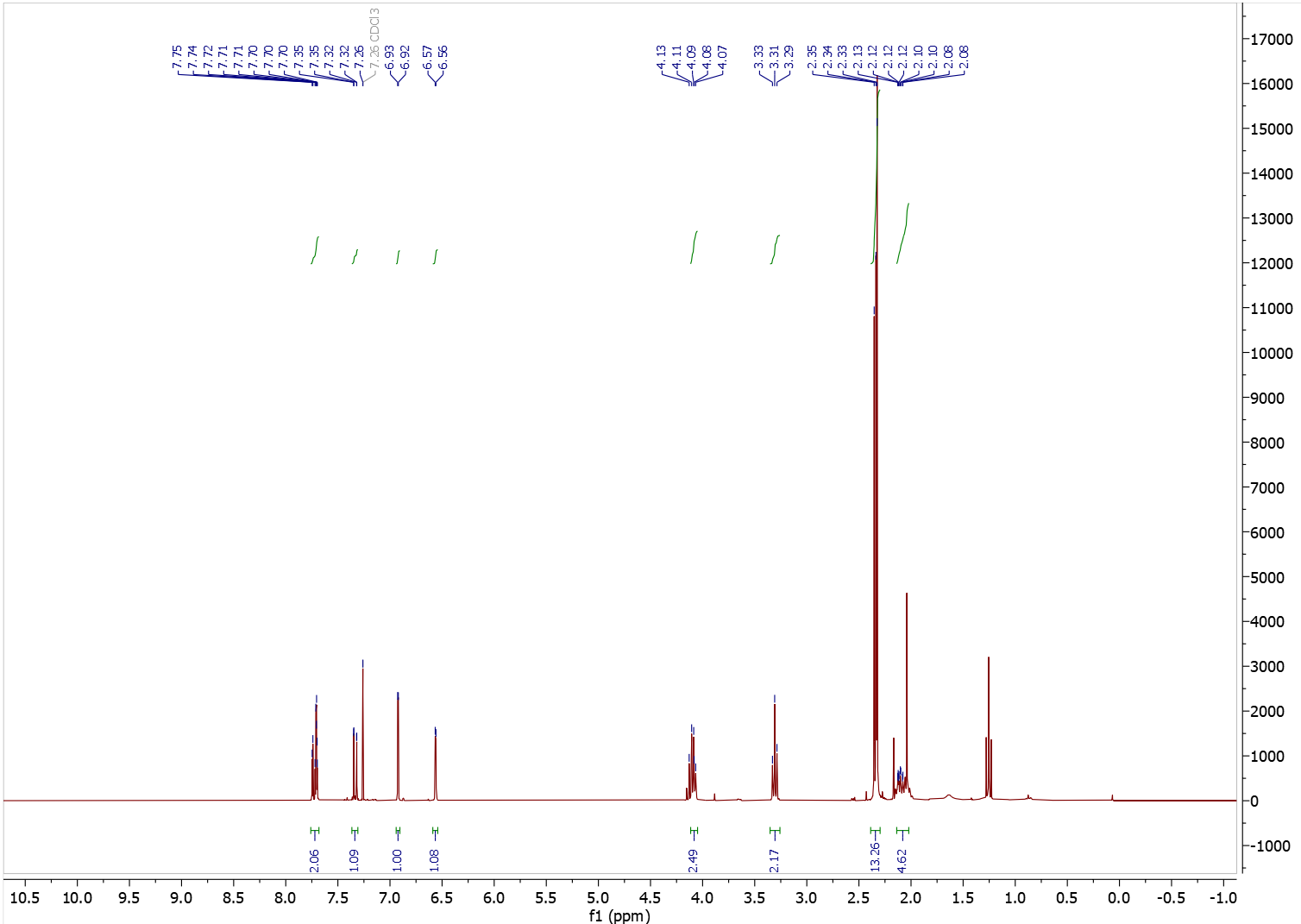

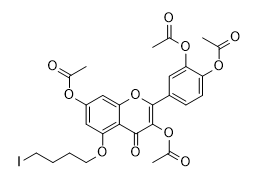


traces of ethyl acetate

traces of ethyl acetate

**Figure S20.** ^1^H NMR (300 MHz, CDCl_3_) spectrum of 4-(3,7-diacetoxy-5-(4-iodobutoxy)-4-oxo-4H-chromen-2-yl)-1,2-phenylene diacetate.


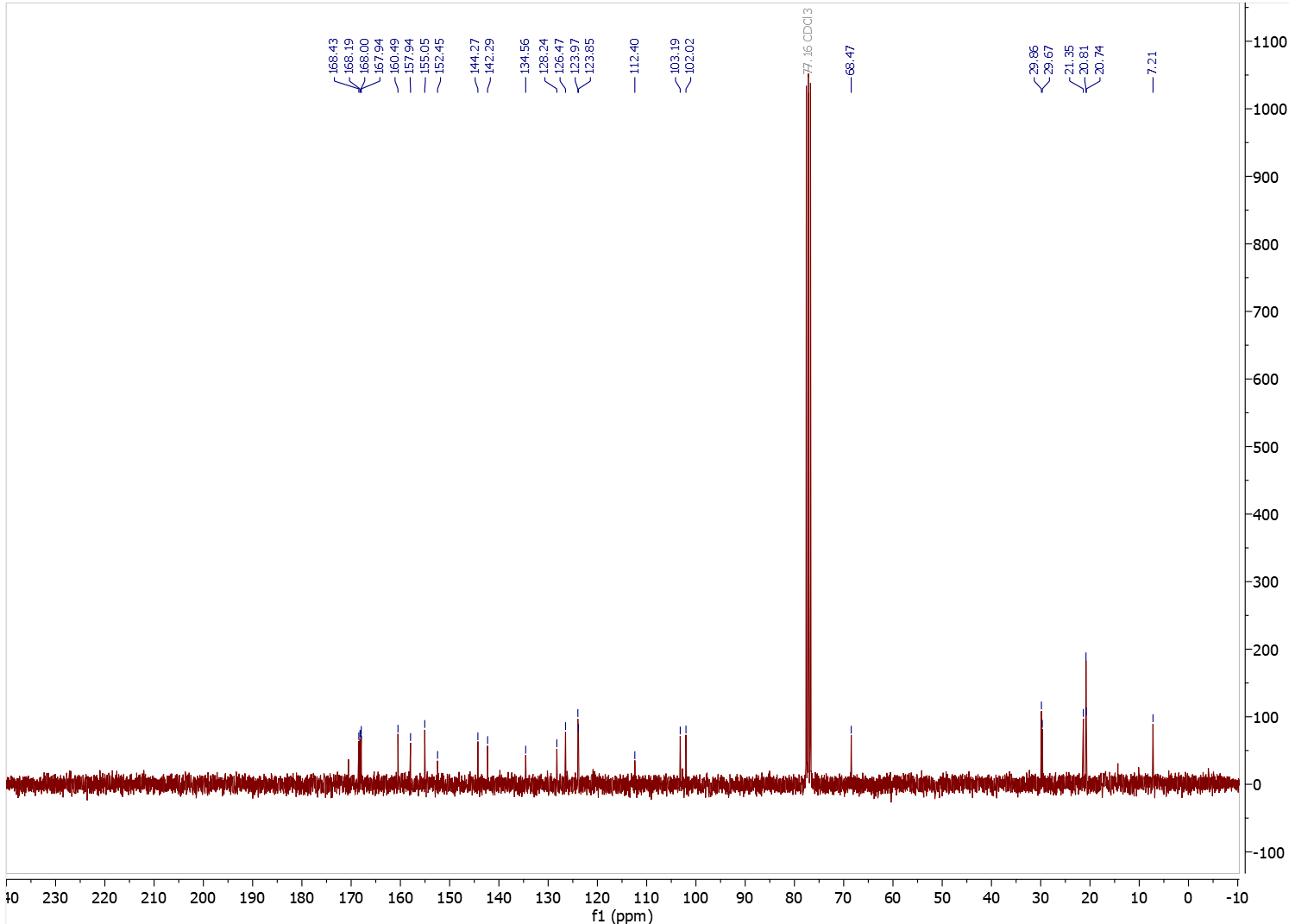

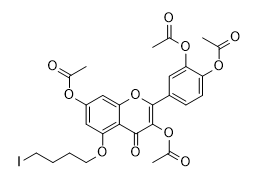


**Figure S21.** ^13^C NMR (75 MHz, CDCl_3_) spectrum of 4-(3,7-diacetoxy-5-(4-iodobutoxy)-4-oxo-4H-chromen-2-yl)-1,2-phenylene diacetate.


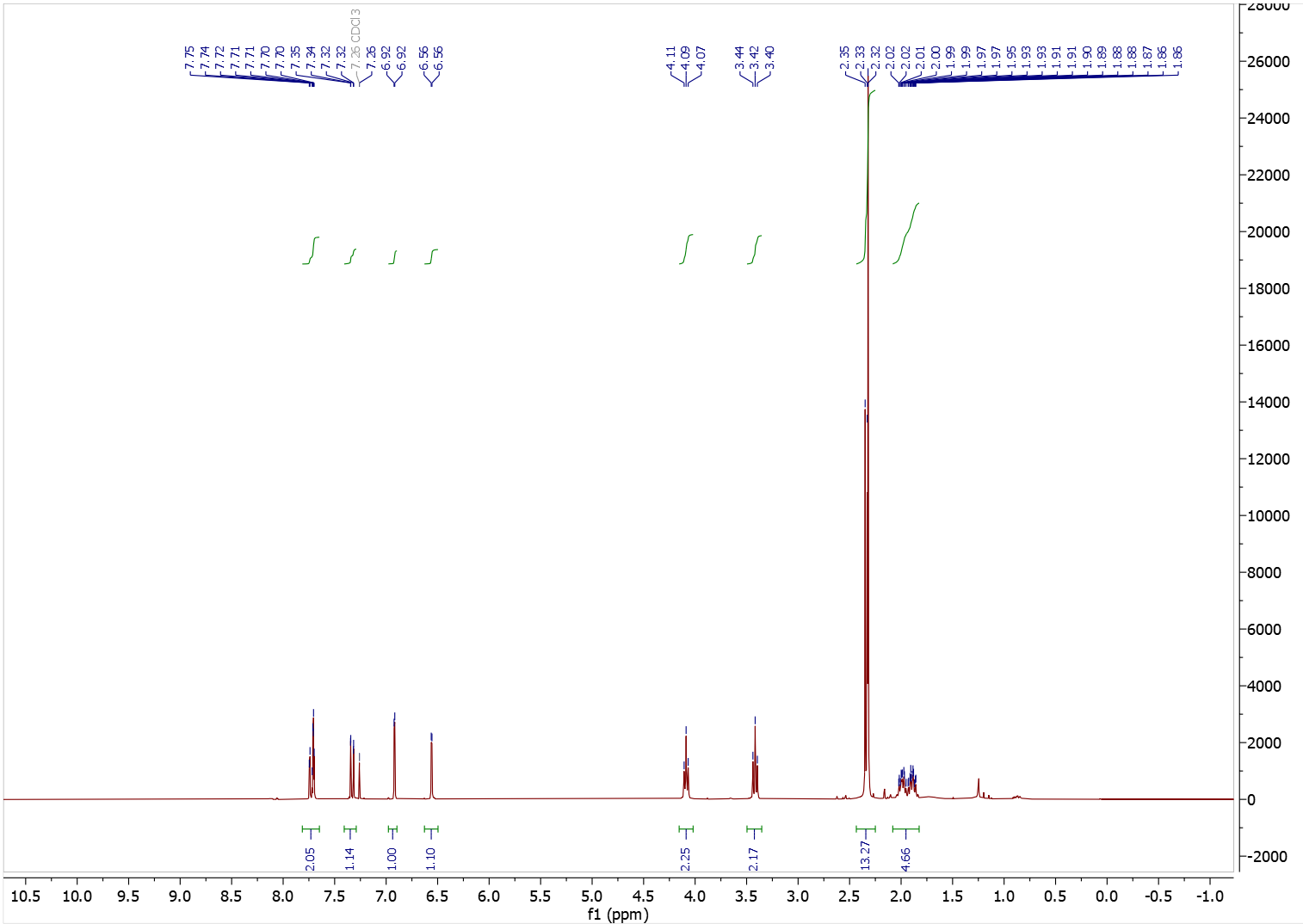

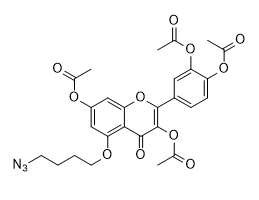


**Figure S22.** ^1^H NMR (300 MHz, CDCl_3_) spectrum of 5-(4-azidobutoxy)-2-(3,4-diacetoxyphenyl)-4-oxo-4H-chromene-3,7-diyl diacetate (**5-N_3_Bu-Q(OAc)_4_**).


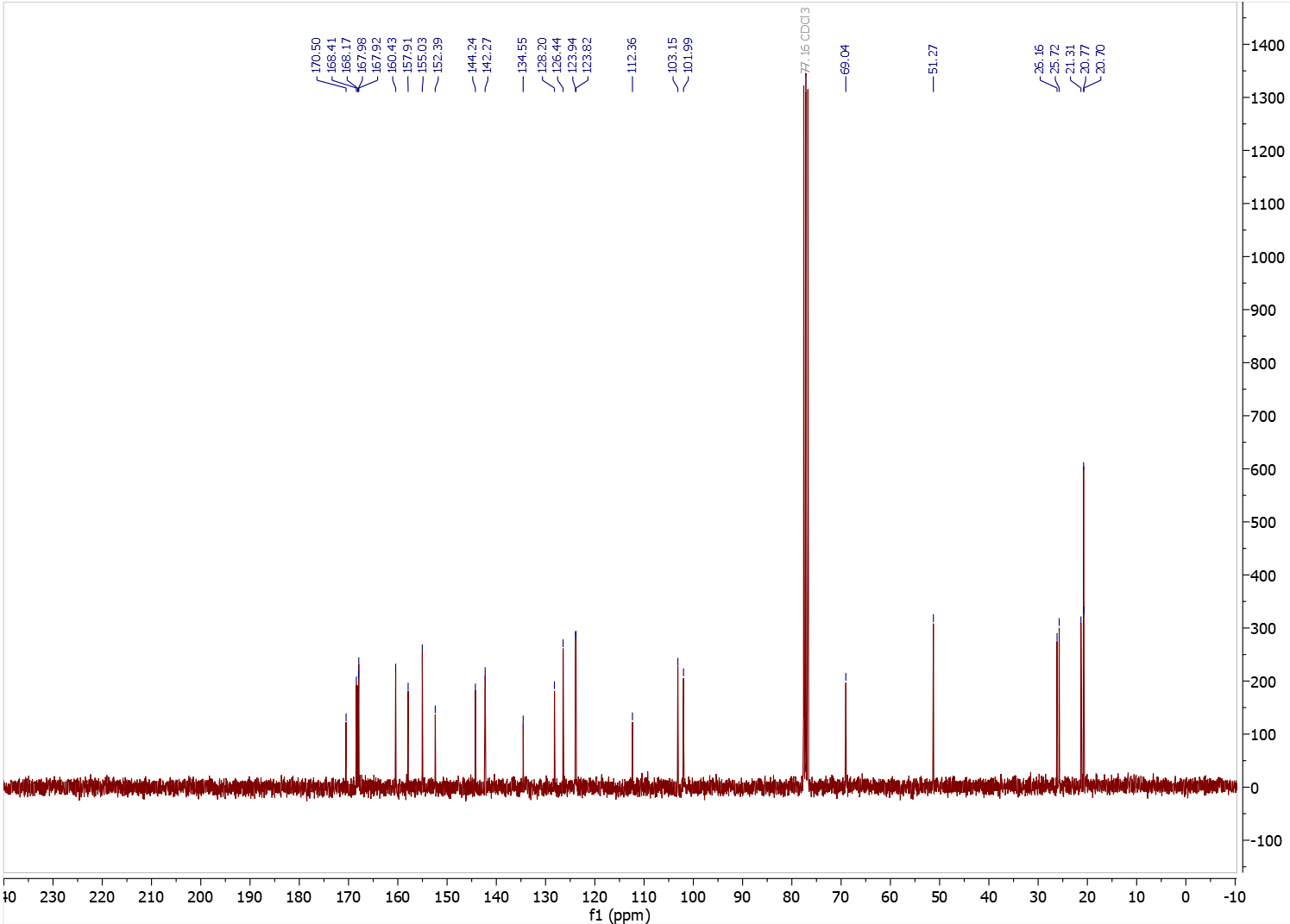

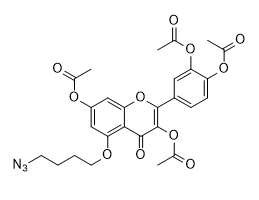


**Figure S23.** ^13^C NMR (75 MHz, CDCl_3_) spectrum of 5-(4-azidobutoxy)-2-(3,4-diacetoxyphenyl)-4-oxo-4H-chromene-3,7-diyl diacetate (**5-N_3_Bu-Q(OAc)_4_**).

**3.3. Compound 7-N_3_Bu-Q(OAc)_4_**

*full name: 7-(4-azidobutoxy)-2-(3,4-diacetoxyphenyl)-4-oxo-4H-chromene-3,5-diyl diacetate*

***Step A***

*full name: 3′,4′-O-diphenylmethane quercetin, or alternative name:*

*2-(2,2-diphenylbenzo[d][1,3]dioxol-5-yl)-3,5,7-trihydroxy-4H-chromen-4-one*

See above in the section 3.1

***Step B***

*full name: 3′,4′-O-diphenylmethane-3,7-diacetyl quercetin, or alternative name:*

*2-(2,2-diphenylbenzo[d][1,3]dioxol-5-yl)-5-hydroxy-4-oxo-4H-chromene-3,7-diyl diacetate*

3′,4′-*O*-diphenylmethane quercetin (1.86 g, 3.99 mmol) was dissolved in 20 ml of DCM and Et_3_N (2.76 ml, 19.9 mmol) was added. The solution was cooled to 0°C and acetic anhydride (0.75 ml, 7.97 mmol) was added dropwise. The reaction mixture was allowed to warm up to RT and it was stirred until starting material disappeared completely (monitored by TLC, with petroleum ether/ethyl acetate 7:1 as an eluent). After finishing, the reaction mixture was diluted by adding 150 ml of DCM and washed 3 times with 100 ml of 1N HCl. Organic phase was dried over anhydrous MgSO_4_. After solvent evaporation, the crude product was recrystallized from ethyl acetate/DCM/petroleum ether mixture to give pure 3′,4′-*O*-diphenylmethane-3,7-diacetyl quercetin as pale yellow solid with isolated yield 70% (1.54 g, 2.80 mmol).

^1^H NMR (300 MHz, CDCl_3_) δ 12.21 (s, 1H), 7.73 – 7.51 (m, 4H), 7.51 – 7.32 (m, 8H), 7.00 (d, *J* = 8.3 Hz, 1H), 6.82 (d, *J* = 2.0 Hz, 1H), 6.58 (d, *J* = 2.0 Hz, 1H), 2.36 (s, 3H), 2.33 (s, 3H). The obtained spectral data are consistent with the literature reports [5].

***Step C***

*full name: 7-(4-O-chlorobutyl)-3′,4′-O-diphenylmethane-3,5-diacetyl quercetin, or alternative name:*

*7-(4-chlorobutoxy)-2-(2,2-diphenylbenzo[d][1,3]dioxol-5-yl)-4-oxo-4H-chromene-3,5-diyl diacetate*

3′,4′-*O*-diphenylmethane-3,7-diacetyl quercetin (1.53 g, 2.77 mmol) and K_2_CO_3_ (0.42 g, 3.05 mmol) were dissolved in DMF (11 ml) and 1-bromo-4-chlorobutane (0.71 g, 4.16 mmol) was then added. The reaction mixture was stirred at RT overnight. After confirming by TLC that all substrate had reacted, the reaction mixture was diluted with ethyl acetate (150 ml), transfer into the separating funnel and washed 3 times with 100 ml of 1N HCl. Organic layer was dried with anhydrous MgSO_4_. The crude product was purified by column chromatography with elucidation by hexane:DCM:ethyl acetate (38:10:2) mixture to give 7-(4-*O*-chlorobutyl)- 3′,4′-*O*-diphenylmethane-3,5-diacetyl quercetin as off-white solid with isolated yield 41% (0.73 g, 1.14 mmol).

^1^H NMR (300 MHz, CDCl_3_) δ 7.69 – 7.50 (m, 4H), 7.50 – 7.30 (m, 8H), 6.97 (dd, *J* = 8.2, 0.5 Hz, 1H), 6.80 (d, *J* = 2.4 Hz, 1H), 6.60 (d, *J* = 2.4 Hz, 1H), 4.07 (d, *J* = 5.5 Hz, 2H), 3.74 – 3.54 (m, 2H), 2.42 (s, 3H), 2.31 (s, 3H), 2.00 (d, *J* = 3.4 Hz, 4H). The obtained spectral data are consistent with the literature reports [5].

**Step D**

*full name: 7-(4-O-chlorobutyl) quercetin, or alternative name:*

*7-(4-chlorobutoxy)-2-(3,4-dihydroxyphenyl)-3,5-dihydroxy-4H-chromen-4-one*

3′,4′-O-Diphenylmethan-3-(4-O-chlorobutyl)quercetin (0.73 g, 1.14 mmol, 1 eq.) was dissolved in a mixture of acetic acid and water (8:2, 50 ml). The solution was heated to reflux and stirred for 2 h. Upon completion (based on TLC), the reaction mixture was cooled to RT, diluted with 200 ml of ethyl acetate, and transferred to a separating funnel. The aqueous phase was neutralized by the gradual addition of a saturated aqueous solution of sodium bicarbonate (approx. 200 ml) until effervescence ceased and the pH was neutral. The organic layer was separated and washed with an additional 100 ml portion of saturated NaHCO_3_ solution. The organic phase was then dried over anhydrous MgSO_4_, filtered, and concentrated under reduced pressure. The crude product was purified by flash column chromatography using a chloroform:acetone (8:2) mixture as eluent to afford 7-(4-O-chlorobutyl)quercetin as yellow solid with the yield of 81% (0.36 g, 0.92 mmol). The product was immediately used in the next synthesis step without further analysis.

**Step E**

*full name: 7-(4-O-chlorobutyl)-3′,4′,3,5-tetraacetyl quercetin, or alternative name:*

*7-(4-chlorobutoxy)-2-(3,4-diacetoxyphenyl)-4-oxo-4H-chromene-3,5-diyl diacetate*

7-(4-O-chlorobutyl) quercetin (0.36 g, 0.92 mmol) was dissolved in 25 ml of DCM and Et_3_N (1.46 ml, 10.5 mmol) was then added. The solution was cooled to 0°C and acetic anhydride (0.83 ml, 8.8 mmol) was added dropwise. The reaction mixture was allowed to warm up to RT and it was stirred until starting material disappeared completely (monitored by TLC, with hexane:acetone 7:3 as an eluent). After finishing (about 3 days of reaction lasting), the reaction mixture was diluted with 150 ml of DCM and washed 3 times with 100 ml of 1N HCl. Organic phase was dried over anhydrous MgSO_4_. After solvent evaporation, the crude product was purified by silica gel column chromatography (20-25% acetone in petroleum ether as eluent) to give a product as a light yellow solid with isolated yield: 49% (0.30 g, 0.53 mmol).

^1^H NMR (300 MHz, CDCl_3_) δ 7.75 – 7.63 (m, 2H), 7.32 (dd, *J* = 8.4, 0.5 Hz, 1H), 6.81 (d, *J* = 2.4 Hz, 1H), 6.61 (d, *J* = 2.4 Hz, 1H), 4.16 – 3.97 (m, 2H), 3.70 – 3.52 (m, 2H), 2.41 (s, 3H), 2.34 – 2.29 (m, 9H), 1.98 (dd, *J* = 3.7, 2.3 Hz, 4H). The obtained spectral data are consistent with the literature reports [6].

***Step F***

*full name: 7-(4-O-iodobutyl)-3′,4′,3,5-tetraacetyl quercetin, or alternative name:*

*4-(3,5-diacetoxy-7-(4-iodobutoxy)-4-oxo-4H-chromen-2-yl)-1,2-phenylene diacetate*

7-(4-O-chlorobutyl)-3′,4′,3,5-tetraacetyl quercetin (0.30 g, 0.53 mmol) and NaI (1.60 g, 10.7 mmol) were dissolved in acetone (5 ml) and heated at reflux overnight. Next day, small amount of solution was taken, evaporated under reduced pressure and measured by ^1^H NMR to calculate the reaction conversion (Rf of substrate and product are the same). If there was no substrate traces on ^1^H NMR spectrum, the reaction mixture was cooled, diluted with 100 ml of ethyl acetate, filtered through paper filter directly into the separating funnel and washed 3 times with 50 ml of water. Organic layer was dried with anhydrous MgSO_4_. The crude product (0.268 g, 0.41 mmol, 77% of isolated yield) was pure enough to be used in the next step without further purification.

^1^H NMR (300 MHz, CDCl_3_) δ 7.77 – 7.57 (m, 2H), 7.33 (dd, *J* = 8.4, 0.8 Hz, 1H), 6.81 (dd, *J* = 2.4, 0.9 Hz, 1H), 6.62 (dd, *J* = 2.5, 0.9 Hz, 1H), 4.07 (t, *J* = 5.7 Hz, 2H), 3.39 – 3.14 (m, 2H), 2.42 (s, 3H), 2.36-2.26 (m, 9H), 2.09 – 1.90 (m, 4H).

^13^C NMR (75 MHz, CDCl_3_) δ 170.1, 169.7, 168.2, 168.0, 167.9, 163.2, 158.2, 153.2, 150.8, 144.3, 142.3, 134.0, 128.2, 126.5, 124.0, 123.8, 111.2, 109.2, 99.4, 67.8, 29.9, 29.8, 21.2, 20.8, 20.7, 6.0.

***Step G***

*full name: 7-(4-O-azidobutyl)-3′,4′,3,5-tetraacetyl quercetin* (7-N_3_Bu-Q(OAc)_4_ )*, or alternative name:*

*7-(4-azidobutoxy)-2-(3,4-diacetoxyphenyl)-4-oxo-4H-chromene-3,5-diyl diacetate*

7-(4-O-Iodobutyl)-3′,4′,3,5-tetraacetylquercetin (0.245 g, 0.38 mmol) was placed in a round-bottom flask equipped with dried molecular sieves and sealed with a septum. The atmosphere was exchanged for nitrogen by performing three vacuum–nitrogen cycles. Then, 2 ml of dry DMF was added, followed by the slow dropwise addition of a solution of sodium azide (49 mg, 0.75 mmol) in 3 ml of dry DMF. The reaction was monitored by taking 50 μl aliquots every 20 min and recording the ¹H NMR spectrum in CDCl_3_. After 1 h, the reaction was quenched. The mixture was diluted with 100 ml of ethyl acetate and washed three times with 50 ml portions of water. The organic layer was dried over anhydrous MgSO_4_, filtered, and concentrated under reduced pressure. Purification by column chromatography using a petroleum ether:acetone (8:2) mixture as eluent afforded 7-(4-O-azidobutyl)-3′,4′,3,5-tetraacetyl quercetin (7-N_3_Bu-Q(OAc)_4_) as a light yellow solid (0.110 g, 0.19 mmol), corresponding to a 51% isolated yield.

^1^H NMR (300 MHz, CDCl_3_) δ 7.79 – 7.63 (m, 2H), 7.33 (dd, *J* = 8.4, 0.5 Hz, 1H), 6.81 (d, *J* = 2.4 Hz, 1H), 6.61 (d, *J* = 2.4 Hz, 1H), 4.06 (t, *J* = 6.0 Hz, 2H), 3.37 (t, *J* = 6.6 Hz, 2H), 2.42 (s, 3H), 2.34 – 2.29 (m, 9H), 2.04 – 1.70 (m, 4H).

^13^C NMR (75 MHz, CDCl_3_) δ 170.1, 169.6, 168.1, 168.0, 167.9, 163.2, 158.2, 153.2, 150.8, 144.3, 142.3, 133.9, 128.2, 126.5, 124.0, 123.8, 111.1, 109.2, 99.4, 68.3, 51.1, 31.0, 26.2, 25.6, 21.2, 20.8, 20.7, 20.6.


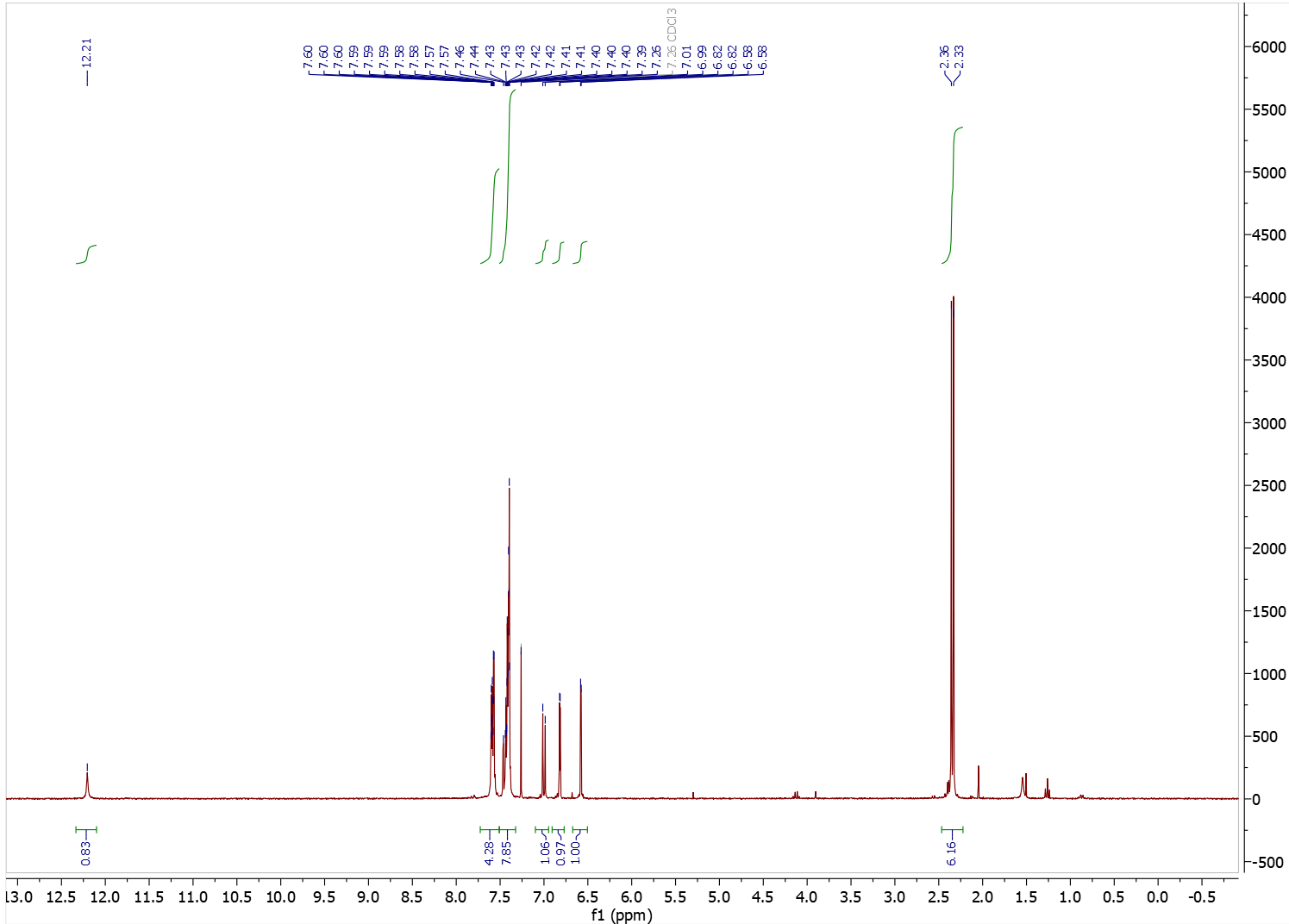

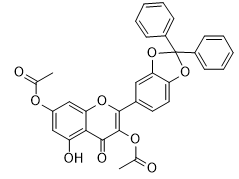


**Figure S24.** ^1^H NMR (300 MHz, CDCl_3_) spectrum of 2-(2,2-diphenylbenzo[d][1,3]dioxol-5-yl)-5-hydroxy-4-oxo-4H-chromene-3,7-diyl diacetate.


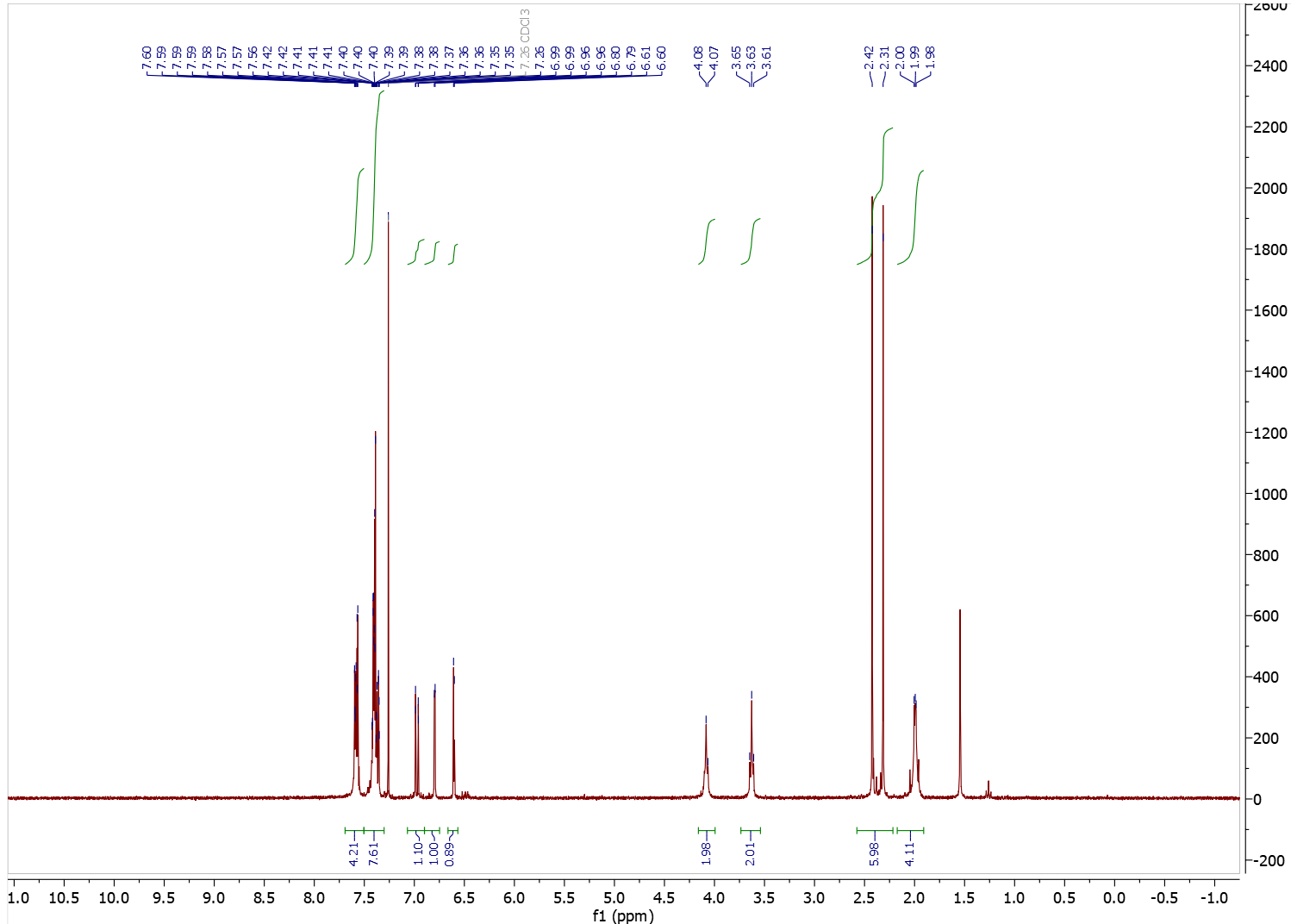

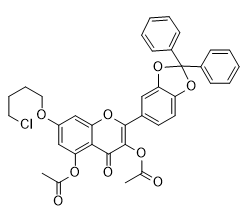


**Figure S25.** ^1^H NMR (300 MHz, CDCl_3_) spectrum of 7-(4-chlorobutoxy)-2-(2,2-diphenylbenzo[d][1,3]dioxol-5-yl)-4-oxo-4H-chromene-3,5-diyl diacetate.


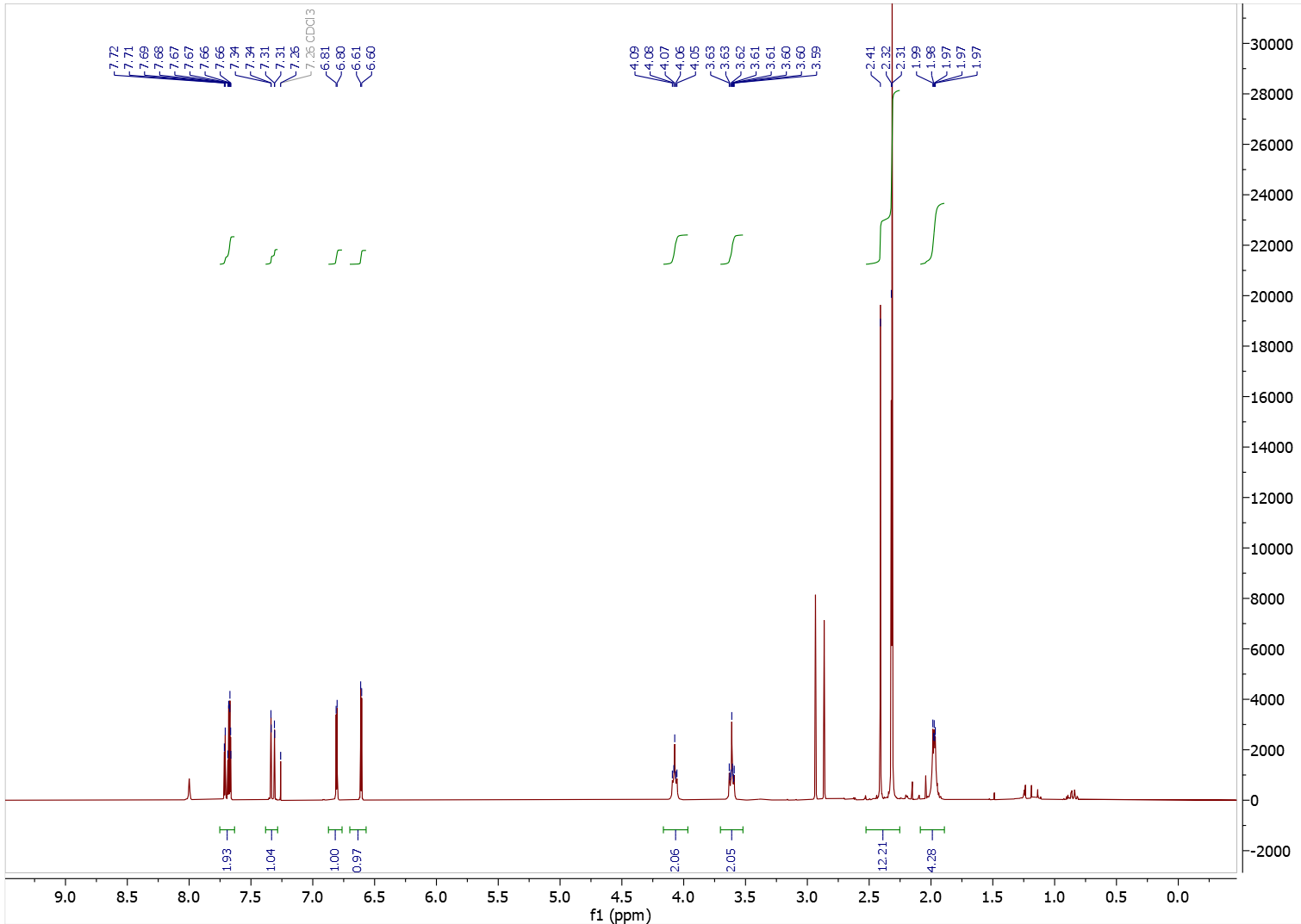

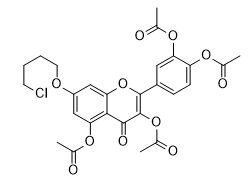


**Figure S26.** ^1^H NMR (300 MHz, CDCl_3_) spectrum of 7-(4-chlorobutoxy)-2-(3,4-diacetoxyphenyl)-4-oxo-4H-chromene-3,5-diyl diacetate.


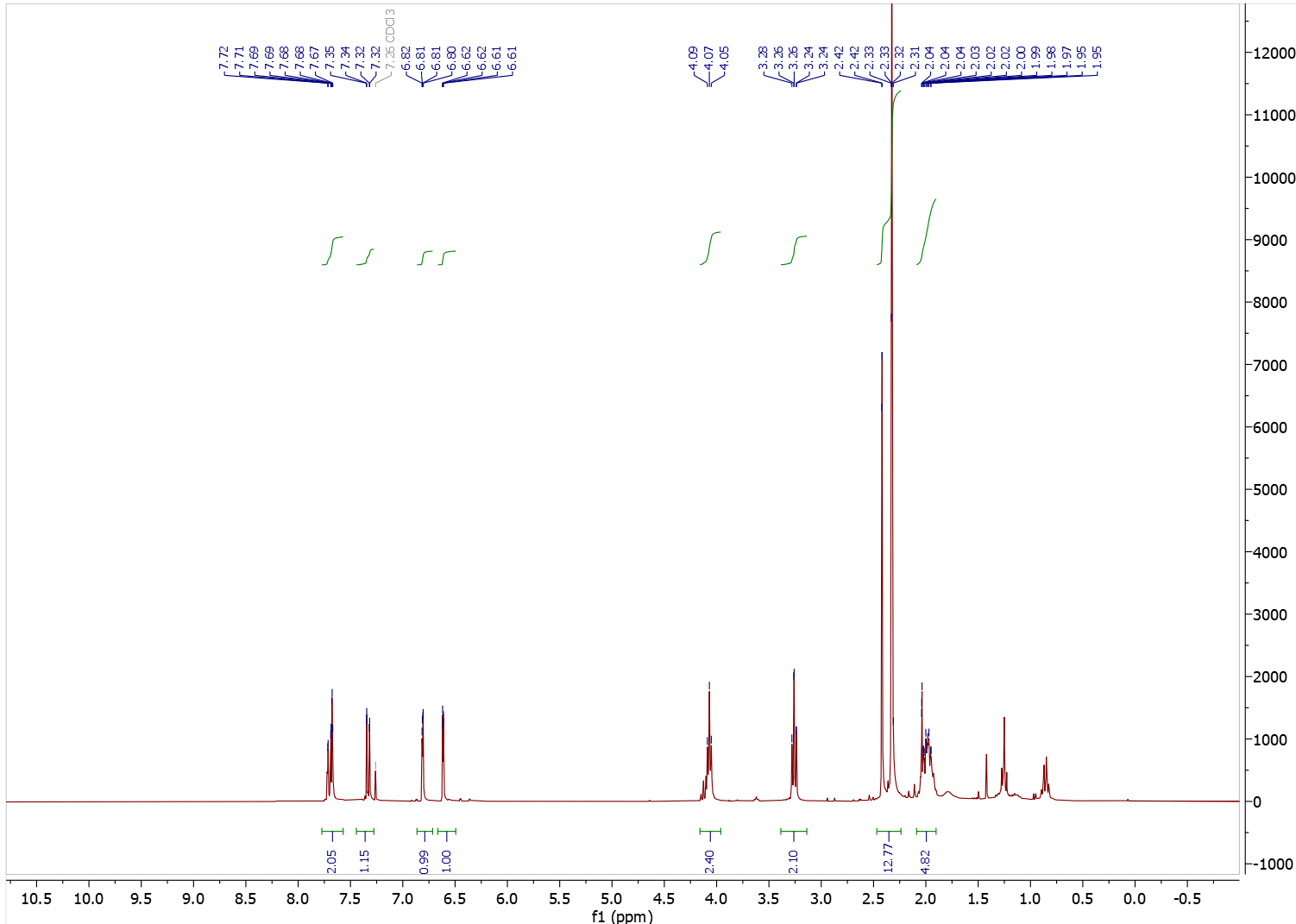

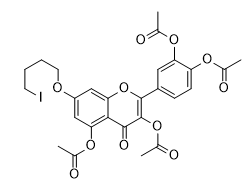


**Figure S27.** ^1^H NMR (300 MHz, CDCl_3_) spectrum of 4-(3,5-diacetoxy-7-(4-iodobutoxy)-4-oxo-4H-chromen-2-yl)-1,2-phenylene diacetate.


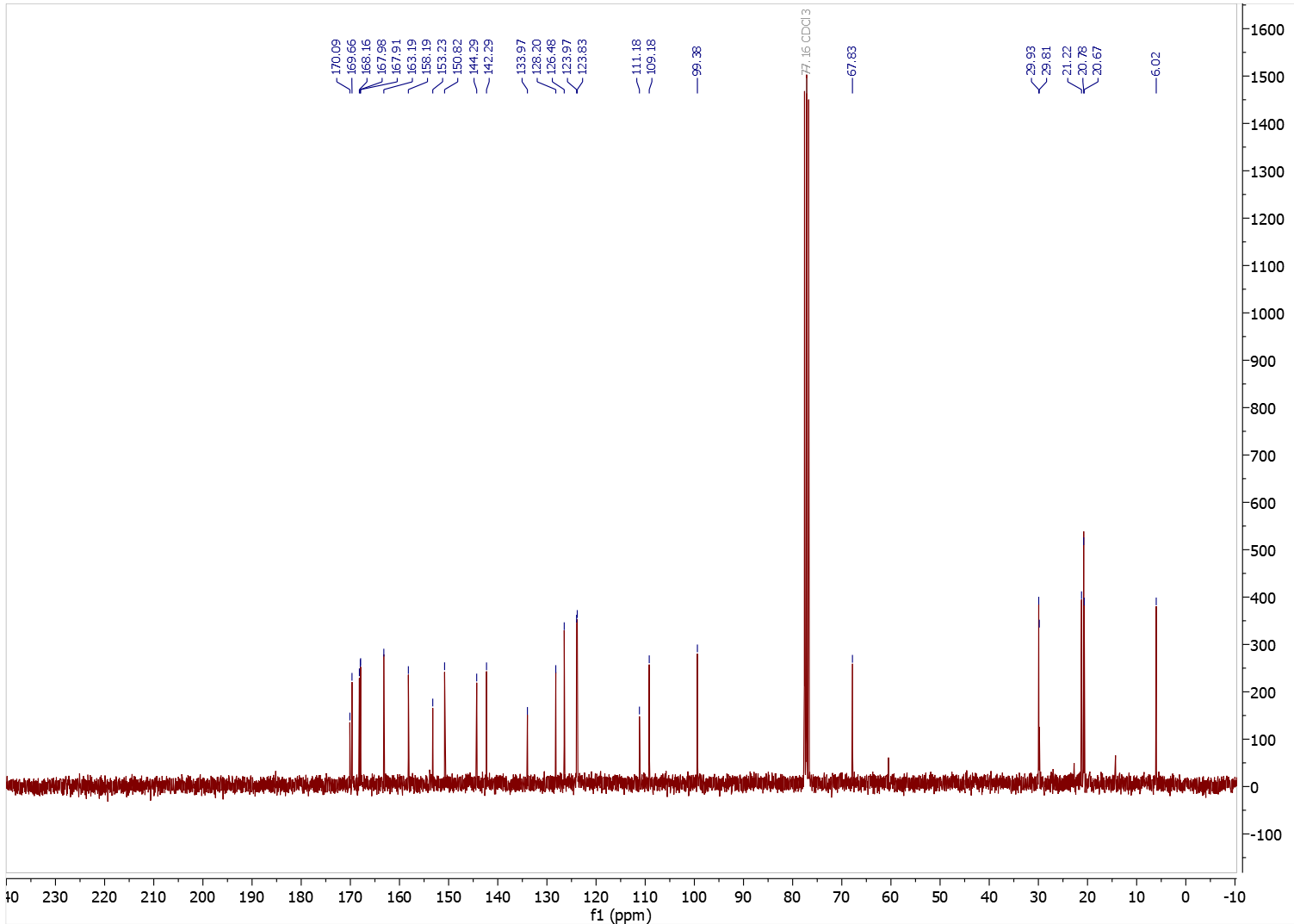

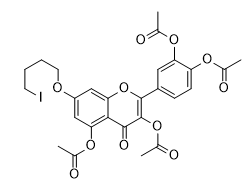


**Figure S28.** ^13^C NMR (75 MHz, CDCl_3_) spectrum of 4-(3,5-diacetoxy-7-(4-iodobutoxy)-4-oxo-4H-chromen-2-yl)-1,2-phenylene diacetate.


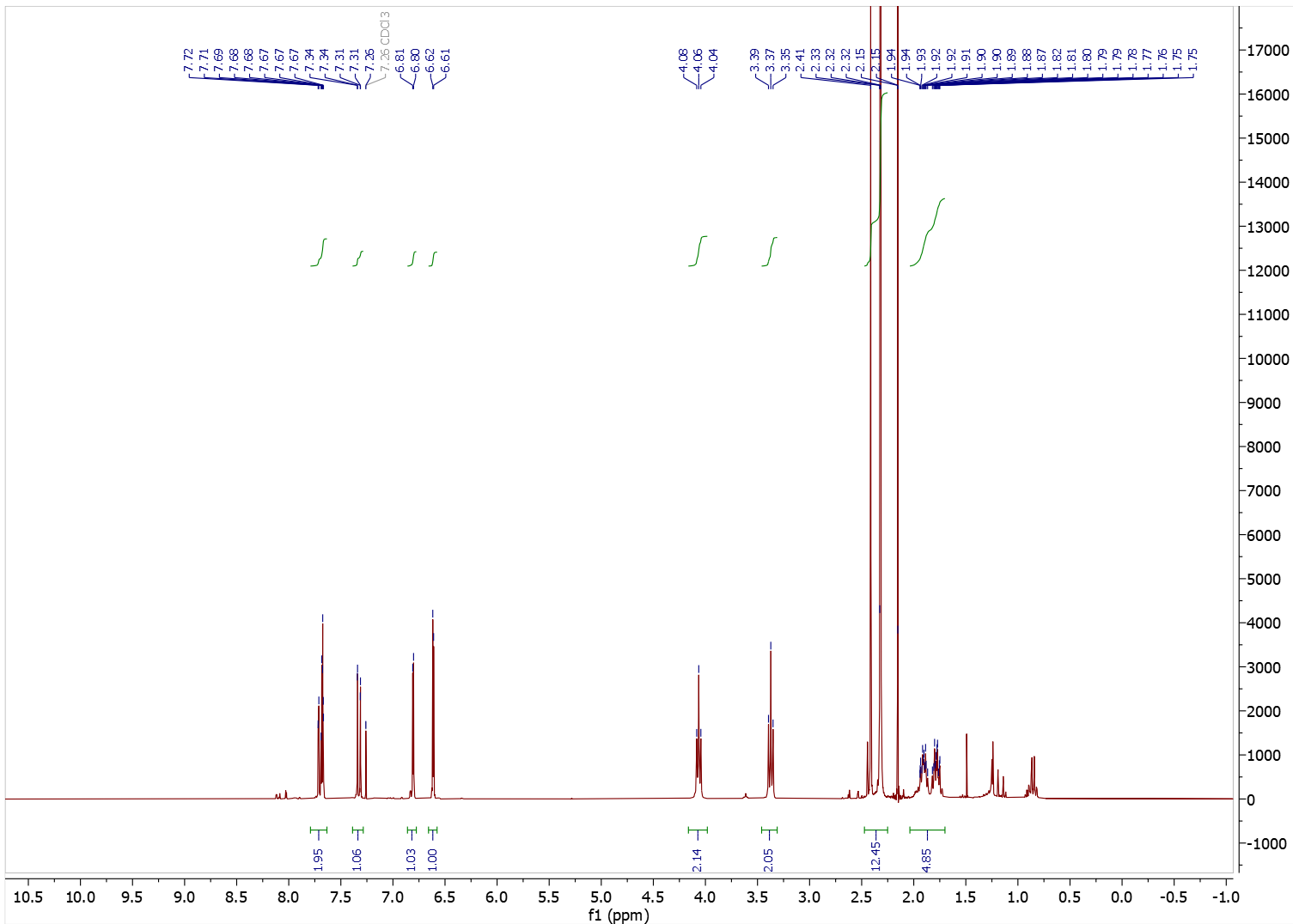

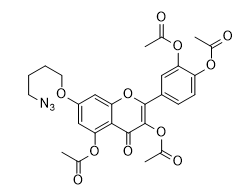


traces of petroleum ether

**Figure S29.** ^1^H NMR (300 MHz, CDCl_3_) spectrum of 7-(4-azidobutoxy)-2-(3,4-diacetoxyphenyl)-4-oxo-4H-chromene-3,5-diyl diacetate (**7-N_3_Bu-Q(OAc)_4_**).


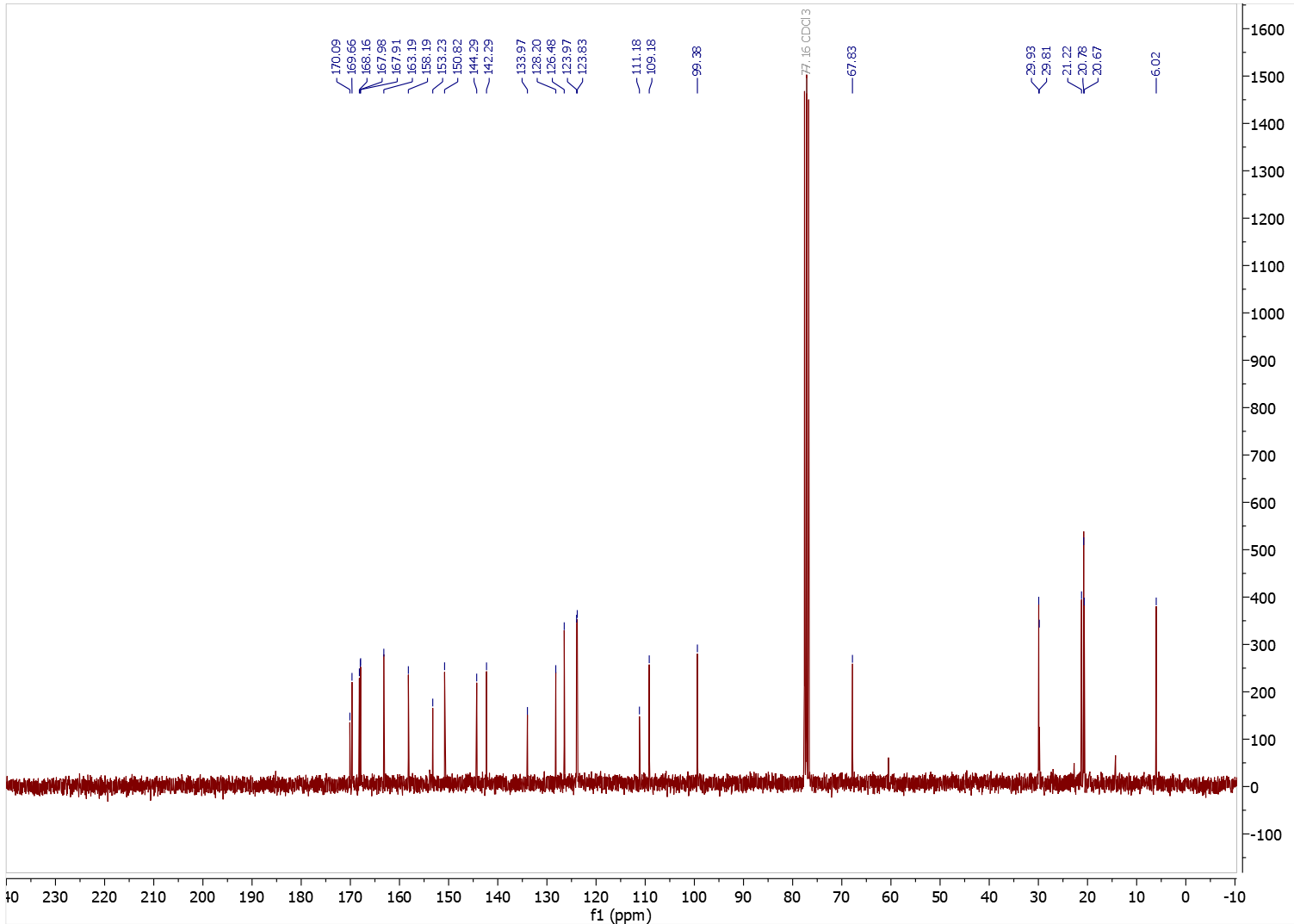

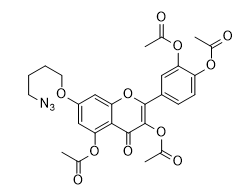


**Figure S30.** ^13^C NMR (75 MHz, CDCl_3_) spectrum of 7-(4-azidobutoxy)-2-(3,4-diacetoxyphenyl)-4-oxo-4H-chromene-3,5-diyl diacetate (**7-N_3_Bu-Q(OAc)_4_**).

**4. Preparation of aza-quercetin (azaQ) and its derivatives.**

**4.1. Compound azaQ(OMe)_5_**

*Full name: 2-(3,4-dimethoxyphenyl)-3,5,7-trimethoxyquinolin-4(1H)-one*

Synthesis of azaQ(OMe)_5_ involved three steps A-C described below.

***Step A***

A cooled solution of 3,5-dimethoxyaniline (3.8 g, 0.025 mol) in CH_2_Cl_2_ (30 ml) under a nitrogen atmosphere was added to BCl_3_ (1.0 M in CH_2_Cl_2_, 25 ml, 0.025 mol). The resulting suspension was stirred in an ice bath for 45 min. Subsequently, methoxyacetonitrile (1.95 g, 2.04 ml, 0.0275 mol) was added, followed by TiCl_4_ (1.0 M in CH_2_Cl_2_, 3.25 ml, 0.00325 mol). The reaction mixture was then heated at reflux for 22 h. After cooling to RT (25°C), 2 M HCl (50 ml) was added dropwise. The mixture was again heated at reflux for 1 h, cooled, and basified with 5 M NaOH (20 ml). The product was extracted with DCM. Organic layers were combined, dried with MgSO_4_, filtered and concentrated *in vacuo* to receive tan solid (2.86 g, 51%) [7].

^1^H NMR (300 MHz, DMSO) δ 7.38 (s, 2H), 5.90 (d, J = 2.4 Hz, 1H), 5.74 (d, J = 2.4 Hz, 1H), 4.37 (s, 2H), 3.77 (s, 3H), 3.72 (s, 3H), 3.28 (s, 3H).

***Step B***

A solution of 3,5-dimethoxy-2-(methoxyacetyl)aniline ( 2.20 g, 9.77 mmol) in pyridine (50 ml) was treated with 3,4-dimethoxybenzoyl chloride (2.10 g, 10.5 mmol). The reaction mixture was heated to reflux under an atmosphere of nitrogen for 18 h. After completion, the mixture was poured into crushed ice (500 ml). The resulting precipitate was collected by vacuum filtration, washed with water, and air-dried to afford the compound as an faint pink solid (2.62 g, 69%) [7].

^1^H NMR (300 MHz, CDCl3) δ 13.23 (s, 1H), 8.38 (d, J = 2.4 Hz, 1H), 7.75 (dd, J = 8.5, 2.2 Hz, 1H), 7.65 (d, J = 2.2 Hz, 1H), 6.96 (d, J = 8.5 Hz, 1H), 6.21 (d, J = 2.4 Hz, 1H), 4.63 (s, 2H), 3.99 (s, 3H), 3.96 (s, 3H), 3.93 (s, 3H), 3.91 (s, 3H), 3.50 (s, 3H).

***Step C***

A mixture of the substrate (1.01 g, 2.59 mmol) and sodium *tert*-butoxide (1.27 g, 13.25 mmol) in *t*‑butanol (38 ml) was stirred in a sealed tube at 80°C overnight. The reaction mixture was cooled to RT and quenched with saturated aqueous ammonium chloride (128 ml). The aqueous phase was extracted with DCM/propan-2-ol (5:1, 4 × 77 ml), water (50 ml) and DCM/propan-2-ol (30 ml). The combined organic extracts were dried with MgSO_4_, filtered and concentrated under reduced pressure. The crude residue was purified by column chromatography (dichloromethane/methanol 20:1) to afford the flavone product as tan powder (0.69 g, 72%) [8].

^1^H NMR (300 MHz, DMSO-d6) δ 10.95 (s, 1H), 7.25 – 7.17 (m, 2H), 7.11 (d, *J* = 9.0 Hz, 1H), 6.67 (d, *J* = 2.3 Hz, 1H), 6.26 (d, *J* = 2.2 Hz, 1H), 3.83 (s, 3H), 3.83 (s, 3H), 3.80 (s, 3H), 3.78 (s, 3H), 3.60 (s, 3H)

^13^C NMR (75 MHz, DMSO-d6) δ 171.8, 161.5, 160.8, 149.7, 148.2, 142.8, 139.7, 139.1, 124.3, 121.7, 112.6, 111.4, 111.0, 93.8, 91.22, 59.0, 55.6, 55.2, 54.9.

**4.2. Compound azaQ**

*Full name: 2-(3,4-dihydroxyphenyl)-3,5,7-trihydroxyquinolin-4(1H)-one*

A sample of the substrate (0.088 g, 0.24 mmol) was dissolved in 48% aqueous hydrobromic acid (10 ml, 88 mmol) and the mixture was heated under reflux for 4 h. After cooling to RT, the solution was neutralized with saturated aqueous sodium bicarbonate, leading to the formation of a precipitate. The solid was collected by vacuum filtration, washed thoroughly with water, and dried under vacuum to give the product as a green solid (0.044 g 62%) [7, 8].

^1^H NMR (300 MHz, DMSO-d6) δ 14.09 (s, 1H), 11.29 (s, 1H), 10.02 (s, 1H), 9.30 (s, 2H), 8.22 (s, 1H), 7.19 (d, J = 2.2 Hz, 1H), 7.04 (dd, J = 8.2, 2.2 Hz, 1H), 6.87 (d, J = 8.3 Hz, 1H), 6.43 (d, J = 2.1 Hz, 1H), 5.96 (d, J = 2.1 Hz, 1H).

^13^C NMR (75 MHz, DMSO-d6) δ 172.3, 161.2, 146.8, 144.9, 140.0, 134.6, 133.6, 122.8, 120.7, 116.7, 115.3, 105.5, 96.2, 91.1.

HMRS (ESI) calc. for [M+H]^+^ (m/z) C_15_H_11_NO_6_ 302.0659 found 302.0665.

**4.3. Compound acyl-azaQ(OAc)_5_**

*Full name: N-acetyl-2-(3,4- diacetoxyphenyl)-3,5,7-triacetoxyquinolin-4(1H)-one.*

Suspension of aza-quercetin (30 mg, 0.10 mmol) in acetic anhydride (10 ml) was treated dropwise with triethylamine (1 ml, 7.2 mmol). The reaction mixture was stirred at RT for 48 h. The mixture changed color to brown. After completion of the reaction (monitored by TLC), the mixture was poured into water and extracted with EtOAc, dried over anhydrous MgSO_4_ and concentrated *in vacuo*. To remove traces of acetic acid, toluene was added and evaporated under reduced pressure. Product was purified by flash column chromatography (DCM : EtOAc; 4:1) to afford a yellowish product (23 mg, 42%).

^1^H NMR (300 MHz, Chloroform-*d*) δ 7.84 (d, *J* = 2.2 Hz, 1H), 7.75 (dd, *J* = 8.4, 2.1 Hz, 1H), 7.66 (d, *J* = 2.0 Hz, 1H), 7.33 (d, *J* = 8.5 Hz, 1H), 7.15 (d, *J* = 2.2 Hz, 1H), 2.42 (s, 3H), 2.38 (s, 3H), 2.35 (s, 3H), 2.32 (s, 3H), 2.31 (s, 3H), 2.24 (s, 3H).

^13^C NMR (75 MHz, Chloroform-d) δ 168.6, 168.6, 168.172, 168.0, 167.8, 167.0, 154.2, 150.8, 148.5, 145.1, 145.1, 143.4, 141.9, 135.2, 134.8, 127.5, 124.4, 123.8, 119.4, 117.4, 115.4, 21.3, 21.2, 20.8, 20.8, 20.7, 20.5.

HMRS (ESI) calc. for [M+H]^+^ (m/z) C_27_H_23_NO_12_ 554.1293 found 554.1283.

**Figure S31.** ^1^H NMR (300 MHz, DMSO) of 3,5-dimethoxy-2-(methoxyacetyl)aniline.

**Figure S32.** ^1^H NMR (300 MHz, DMSO) of N-(3,5-dimethoxy-2-methoxyacetylphenyl)-3,4-dimethoxybenzamide.

**Figure S33.** ^1^H NMR (300 MHz, DMSO) of 2-(3,4-dimethoxyphenyl)-3,5,7-trimethoxyquinolin-4(1H)-one (**azaQ(OMe)_5_**).

**Figure S34.** ^13^C NMR (75 MHz, DMSO) of 2-(3,4-dimethoxyphenyl)-3,5,7-trimethoxyquinolin-4(1H)-one (**azaQ(OMe)_5_**).

**Figure S35.** ^1^H NMR (300 MHz, DMSO) of 2-(3,4-dihydroxyphenyl)-3,5,7-trihydroxyquinolin-4(1H)-one (**azaQ**).


**Figure S36.** ^13^C NMR (75MHz, DMSO) of 2-(3,4-dihydroxyphenyl)-3,5,7-trihydroxyquinolin-4(1H)-one (**azaQ**).

**Figure S37.** ^1^H NMR (300 MHz, DMSO) of N-acetyl-2-(3,4- diacetoxyphenyl)-3,5,7-triacetoxyquinolin-4(1H)-one (**acyl-azaQ(OAc)_5_**).

**Figure S38.** ^13^C NMR (75 MHz, DMSO) of N-acetyl-2-(3,4- diacetoxyphenyl)-3,5,7-triacetoxyquinolin-4(1H)-one (**acyl-azaQ(OAc)_5_**).

**5. Preparation of thioquercetin (thioQ) and its derivatives.**

**Scheme S3.** Overall synthetic pathway for thioQ and its derivatives. Conditions: **(a)** MeI, K_2_CO_3_, DMF, rt.; **(b)** 1. Lawesson’s reagent, THF, RT, 2. BBr_3_, DCM, RT; (c) Lawesson’s reagent, PhMe, 100°C; **(d)** Et_3_N, Ac_2_O, DCM, RT.

**5.1. Compound thioQ**

*Full name: 2-(3,4-dihydroxyphenyl)-3,5,7-trihydroxy-4H-chromene-4-thione*

2-(3,4-dimethoxyphenyl)-3,5,7-trimethoxy-4H-chromen-4-one (2.00 g, 5.37 mmol, 1 eq.) was dissolved in dry THF (15 ml). Lawesson’s reagent was added (2.54 g, 6.28 mmol, 1.2 eq.) and the mixture was stirred under argon at RT for 72 h. The crude mixture was dried *in vacuo*, dissolved in DCM and filtrated on silica pad. The solvent was removed *in vacuo* and the obtained solid was subjected to the next step without further purification. The obtained solid was dissolved in DCM (10 ml) and cooled in ice/water bath. Boron tribromide (6 ml) was slowly added, and the mixture was stirred at room temperature overnight. DCM was removed *in vacuo* and ice-cold water was slowly added and the resulting suspension was stirred for 1 h. The precipitate was collected by filtration, washed extensively with water and dried *in vacuo*. 2-(3,4-dihydroxyphenyl)-3,5,7-trihydroxy-4H-chromene-4-thione was obtained as a brick-red solid (1.08 g, 3.39 mmol, 63%).

^1^H NMR (300 MHz, DMSO-*d*_6_) *δ* 13.07 (*s*, 1H, OH), 11.08 (*s*, 1H, OH), 9.92 (*s*, 1H, OH), 9.44 (*s*, 1H, OH), 8.66 (*s*, 1H, OH), 7.78 (*d*, *J* = 2.2 Hz, 1H, Ar-H), 7.73 (*dd*, *J* = 8.5, 2.3 Hz, 1H, Ar-H), 6.94 (*d*, *J* = 8.5 Hz, 1H, Ar-H), 6.56 (*d*, *J* = 2.3 Hz, 1H, Ar-H), 6.36 (*d*, *J* = 2.3 Hz, 1H, Ar-H);

^13^C NMR (75 MHz, DMSO-*d*_6_) *δ* 181.7, 163.5, 160.2, 152.6, 149.2, 145.4, 142.7, 142.4, 121.7, 120.8, 115.9, 115.8, 111.3, 100.4, 93.9; ESI-HRMS (m/z) calc. for [M+H]^+^ C_15_H_10_O_6_S 319.0271, found 319.0270. The spectra are consistent with the literature [1].

**5.2. Compound thioQ(OMe)_5_**

*Full name: 2-(3,4-dimethoxyphenyl)-3,5,7-trimethoxy-4H-chromene-4-thione*

2-(3,4-dimethoxyphenyl)-3,5,7-trimethoxy-4H-chromen-4-one (100 mg, 0.27 mmol, 1 eq.) was dissolved in anhydrous toluene (10 ml). Lawesson’s reagent was added (65 mg, 0.16 mmol, 0.6 eq.) and the mixture was stirred at 100°C for 4 h. The crude mixture was dried *in vacuo* and purified by column chromatography on silica (eluent: cyclohexane:chloroform:ethyl acetate 2:4:1 v/v/v). 2-(3,4-dimethoxyphenyl)-3,5,7-trimethoxy-4H-chromene-4-thione was obtained as a red solid (74 mg, 0.19 mmol, 71%).

^1^H NMR (300 MHz, Chloroform-*d*) δ 7.82 (*d*, *J* = 2.1 Hz, 1H, Ar-H), 7.73 (*dd*, *J* = 8.6, 2.1 Hz, 1H, Ar-H), 6.96 (*d*, *J* = 8.6 Hz, 1H, Ar-H), 6.50 (*d*, *J* = 2.5 Hz, 1H, Ar-H), 6.36 (*d*, *J* = 2.5 Hz, 1H, Ar-H), 3.94 (*s*, 3H, OCH_3_), 3.93 (*s*, 3H, OCH_3_), 3.89 (*s*, 6H, 2×OCH_3_), 3.68 (*s*, 3H, OCH_3_). The spectrum is consistent with the literature [1].

**5.3. Compound thioQ(OAc)_4_**

*Full name:*

*2-(4-acetoxy-3-(prop-1-en-2-yloxy)phenyl)-5-hydroxy-4-thioxo-4H-chromene-3,7-diyl diacetate*

2-(3,4-dihydroxyphenyl)-3,5,7-trihydroxy-4H-chromene-4-thione (170 mg, 0.53 mmol, 1 eq.) and Et_3_N (370 µl, 2.65 mmol, 5 eq.) were dissolved in dry DCM (5 ml). Ac_2_O (200 µl, 2.12 mmol, 4 eq.) was added and the mixture was stirred at room temperature until the reaction was completed with regard to TLC. The mixture was dried *in vacuo* and purified by column chromatography on silica (eluent: cyclohexane/ethyl acetate 4:1 v/v). 2-(4-acetoxy-3-(prop-1-en-2-yloxy)phenyl)-5-hydroxy-4-thioxo-4H-chromene-3,7-diyl diacetate was obtained as a yellow solid (137 mg, 0.28 mmol, 53%).

^1^H NMR (300 MHz, Chloroform-*d*) *δ* 13.62 (*s*, 1H, OH), 7.84 – 7.73 (*m*, 2H, Ar-H), 7.39 – 7.35 (m, 1H, Ar-H), 6.91 (*d*, *J* = 2.3 Hz, 1H, Ar-H), 6.71 (*d*, *J* = 2.3 Hz, 1H, Ar-H), 2.38 (*s*, 3H, C(O)CH_3_), 2.34 (*s*, 3H, C(O)CH_3_), 2.33 (*s*, 6H, 2×C(O)CH_3_);

^13^C NMR (75 MHz, Chloroform-*d*) *δ* 192.9, 168.2, 167.9, 167.9, 167.8, 162.4, 155.7, 152.2, 149.4, 144.9, 142.4, 140.4, 127.5, 127.0, 124.4, 124.2, 116.8, 107.1, 101.1, 21.3, 20.9, 20.8; ESI-HRMS (m/z) calc. for [M+H]^+^ C_23_H_18_O_10_S 487.0693, found 487.0693.

**5.4. Compound thioQ(OAc)_5_**

*Full name: 2-(3,4-diacetoxyphenyl)-4-thioxo-4H-chromene-3,5,7-triyl triacetate*

2-(3,4-dihydroxyphenyl)-3,5,7-trihydroxy-4H-chromene-4-thione (235 mg, 0.74 mmol, 1 eq.) and Et_3_N (2.07 ml, 14.8 mmol, 20 eq.) were dissolved in dry DCM (5 ml). Ac_2_O (1.05 ml, 11.1 mmol, 15 eq.) was added and the mixture was stirred at room temperature until the reaction was completed with regard to TLC. The mixture was dried *in vacuo* and purified by column chromatography on silica (eluent: cyclohexane/ethyl acetate 4:1 v/v). 2-(3,4-diacetoxyphenyl)-4-thioxo-4H-chromene-3,5,7-triyl triacetate was obtained as a green solid (203 mg, 0.38 mmol, 52%).

^1^H NMR (300 MHz, Chloroform-*d*) *δ* 7.80 – 7.74 (*m*, 2H, Ar-H), 7.41 (*d*, *J* = 2.4 Hz, 1H, Ar-H), 7.38 – 7.33 (*m*, 1H, Ar-H), 6.91 (*d*, J = 2.4 Hz, 1H, Ar-H), 2.37 (*s*, 3H, C(O)CH_3_), 2.34 (*s*, 3H, C(O)CH_3_), 2.33 (s, 3H, C(O)CH_3_), 2.33 (s, 6H, 2×C(O)CH_3_) ;

^13^C NMR (75 MHz, Chloroform-*d*) *δ* 191.4, 169.4, 167.9, 167.9, 167.9, 153.4, 152.6, 150.6, 147.0, 144.6, 143.1, 142.4, 128.1, 126.9, 124.3, 124.1, 122.5, 115.8, 109.5, 22.0, 21.3, 21.1, 20.8; ESI-HRMS (m/z) calc. for [M+H]^+^ C_25_H_20_O_11_S 529.0799, found 529.0797.

**Figure S39** ^1^H NMR (300 MHz, Chloroform-*d*) of (2-(3,4-dimethoxyphenyl)-3,5,7-trimethoxy-4H-chromene-4-thione) (**thioQ(OMe)_5_**).

**Figure S40.** ^1^H NMR (300 MHz, DMSO-*d_6_*) of (2-(3,4-dihydroxyphenyl)-3,5,7-trihydroxy-4H-chromene-4-thione) (**thioQ**).

**Figure S41.** ^13^C NMR (75 MHz, DMSO-*d_6_*) of (2-(3,4-dihydroxyphenyl)-3,5,7-trihydroxy-4H-chromene-4-thione) (**thioQ**).

**Figure S42.** ^1^H NMR (300 MHz, Chloroform-*d*) of (2-(4-acetoxy-3-(prop-1-en-2-yloxy)phenyl)-5-hydroxy-4-thioxo-4H-chromene-3,7-diyl diacetate) (**thioQ(OAc)_4_**).

**Figure S43.** ^13^C NMR (75 MHz, Chloroform-*d*) of (2-(4-acetoxy-3-(prop-1-en-2-yloxy)phenyl)-5-hydroxy-4-thioxo-4H-chromene-3,7-diyl diacetate) (**thioQ(OAc)_4_**).

**Figure S44.** ^1^H NMR (300 MHz, Chloroform-*d*) of (2-(3,4-diacetoxyphenyl)-4-thioxo-4H-chromene-3,5,7-triyl triacetate) (**thioQ(OAc)_5_**).

**Figure S45.** ^13^C NMR (75 MHz, Chloroform-*d*) of (2-(3,4-diacetoxyphenyl)-4-thioxo-4H-chromene-3,5,7-triyl triacetate) (**thioQ(OAc)_5_**).

**5.5. Determination of purity**

In Section 5.5, the results of the purity analyses of the thio-analogues are presented. A combined analytical method employing High-Performance Liquid Chromatography (HPLC) with a Photodiode Array (PDA) detector and Mass Spectrometry (MS) was used to separate, identify, and quantify the compounds. The analyses were performed using a QTOF LCMS-9030 system coupled with a UHPLC Shimadzu Nexera X3. Each report includes details on the methodology, mobile-phase composition, column conditions, and PDA detection wavelengths, with the main chromatographic peaks confirmed by MS. Although slight variations were observed depending on the detection wavelength, all samples exhibited very high purity. The lowest recorded purity was 97.2% for thioQ, 99.0% for thioQ(OAc)_4_, and 98.7% for thioQ(OAc)_5_. Complete analytical reports are presented below.

**5.5.1. HPLC purity profile of thioQ**

Method: Gradient: 3%-40% B in 9.5 min; Column: Kinetex C18 2.6 um 150 x 2.1 mm

**PDA 200 nm**

| Peak# | Ret. Time | | Area | Height | Area% |
| --- | --- | --- | --- | --- | --- |
| **1** | | **6.528** | **4640476** | **939326** | **100.000** |
| Total | |  | 4640476 | 939326 | 100.000 |

**PDA 262 nm**

| Peak# | Ret. Time | Area | Height | Area% |
| --- | --- | --- | --- | --- |
| 1 | 3.925 | 1375 | 263 | 0.073 |
| 2 | 4.309 | 2115 | 303 | 0.113 |
| 3 | 4.981 | 2427 | 234 | 0.129 |
| 4 | 5.216 | 6704 | 1141 | 0.357 |
| 5 | 5.653 | 2913 | 382 | 0.155 |
| 6 | 5.845 | 3904 | 531 | 0.208 |
| 7 | 6.133 | 11832 | 1161 | 0.63 |
| **8** | **6.528** | **1824938** | **391243** | **97.199** |
| 9 | 7.157 | 8644 | 1779 | 0.46 |
| 10 | 7.243 | 12670 | 1664 | 0.675 |
| Total |  | 1877520 | 398701 | 100 |

**PDA 370 nm**

| Peak# | Ret. Time | | Area | Height | Area% |
| --- | --- | --- | --- | --- | --- |
| 1 | | 4.309 | 1224 | 305 | 0.047 |
| 2 | | 5.216 | 5790 | 1229 | 0.221 |
| 3 | | 5.845 | 1911 | 419 | 0.073 |
| 4 | | 6.133 | 9988 | 1291 | 0.381 |
| **5** | | **6.528** | **2592639** | **529861** | **98.815** |
| 6 | | 7.157 | 5429 | 1532 | 0.207 |
| 7 | | 7.243 | 6740 | 1337 | 0.257 |
| Total | |  | 2623722 | 535975 | 100 |

**PDA 454 nm**

| Peak# | Ret. Time | | Area | Height | Area% |
| --- | --- | --- | --- | --- | --- |
| 1 | | 5.461 | 1058 | 224 | 0.031 |
| 2 | | 5.643 | 1875 | 324 | 0.054 |
| 3 | | 6.133 | 11653 | 1535 | 0.338 |
| **4** | | **6.528** | **3415769** | **704363** | **99.18** |
| 5 | | 7.157 | 6202 | 1748 | 0.18 |
| 6 | | 7.243 | 7454 | 1490 | 0.216 |
| Total | |  | 3444011 | 709683 | 100 |

TIC (+) blank

TIC (+) thioQ

XIC (+) 319.0271 m/z

**5.5.2 HPLC purity profile of thioQ(OAc)_4_**

Method: Gradient: 50%-97% B in 9.5 min; Column: Kinetex C18 2.6 um 150 x 2.1 mm

**PDA 200 nm**

| Peak# | Ret. Time | | Area | Height | Area% |
| --- | --- | --- | --- | --- | --- |
| 1 | | 4.128 | 45593 | 11273 | 0.682 |
| **2** | | **4.288** | **6619410** | **1241685** | **99.064** |
| 3 | | 4.469 | 2653 | 1450 | 0.04 |
| 4 | | 4.576 | 3523 | 1642 | 0.053 |
| 5 | | 5.045 | 10778 | 2394 | 0.161 |
| Total | |  | 6681957 | 1258444 | 100 |

**PDA 330 nm**

| Peak# | Ret. Time | Area | Height | Area% |
| --- | --- | --- | --- | --- |
| 1 | 2.976 | 1387 | 322 | 0.043 |
| 2 | 3.232 | 738 | 172 | 0.023 |
| 3 | 4.128 | 10372 | 2556 | 0.318 |
| **4** | **4.288** | **3239823** | **633967** | **99.366** |
| 5 | 4.469 | 1225 | 674 | 0.038 |
| 6 | 4.565 | 1445 | 683 | 0.044 |
| 7 | 5.045 | 5511 | 1234 | 0.169 |
| Total |  | 3260502 | 639607 | 100 |

TIC (+) blank

TIC (+) thioQ(OAc)_4_

XIC (+) 487.0690 m/z

**5.5.3. HPLC purity profile of thioQ(OAc)_5_**

Method: Gradient: 3%-97% B in 9.5 min; Column: Kinetex C18 2.6 um 150 x 2.1 mm

**PDA 200 nm**

| Peak# | Ret. Time | | Area | Height | Area% |
| --- | --- | --- | --- | --- | --- |
| **1** | | **7.819** | **1644264** | **356371** | **99** |
| 2 | | 7.989 | 4042 | 2201 | 0.243 |
| 3 | | 8.064 | 6087 | 2431 | 0.367 |
| 4 | | 8.971 | 6482 | 2758 | 0.39 |
| Total | |  | 1660874 | 363761 | 100 |

**PDA 240 nm**

| Peak# | Ret. Time | Area | Height | Area% |
| --- | --- | --- | --- | --- |
| 1 | 6.763 | 1144 | 144 | 0.128 |
| 2 | 7.104 | 2792 | 567 | 0.312 |
| 3 | 7.659 | 2185 | 441 | 0.244 |
| **4** | **7.819** | **882642** | **173425** | **98.704** |
| 5 | 7.989 | 1782 | 962 | 0.199 |
| 6 | 8.064 | 3031 | 1189 | 0.339 |
| 7 | 8.309 | 658 | 176 | 0.074 |
| Total |  | 894232 | 176904 | 100 |

**PDA 365 nm**

| Peak# | Ret. Time | | Area | Height | Area% |
| --- | --- | --- | --- | --- | --- |
| 1 | | 7.093 | 2958 | 547 | 0.476 |
| 2 | | 7.659 | 1197 | 279 | 0.193 |
| **3** | | **7.819** | **613219** | **122042** | **98.76** |
| 4 | | 7.989 | 1194 | 654 | 0.192 |
| 5 | | 8.064 | 1429 | 598 | 0.23 |
| 6 | | 8.309 | 923 | 230 | 0.149 |
| Total | |  | 620919 | 124350 | 100 |

**PDA 385 nm**

| Peak# | Ret. Time | | Area | Height | Area% |
| --- | --- | --- | --- | --- | --- |
| 1 | | 7.093 | 2821 | 498 | 0.322 |
| 2 | | 7.659 | 1297 | 317 | 0.148 |
| **3** | | **7.819** | **865892** | **172307** | **98.905** |
| 4 | | 7.989 | 1961 | 1040 | 0.224 |
| 5 | | 8.064 | 2620 | 1033 | 0.299 |
| 6 | | 8.309 | 888 | 219 | 0.101 |
| Total | |  | 875479 | 175414 | 100 |

TIC (+) blank

TIC (+) thioQ(OAc)_5_

XIC (+) 529.0799 m/z

**
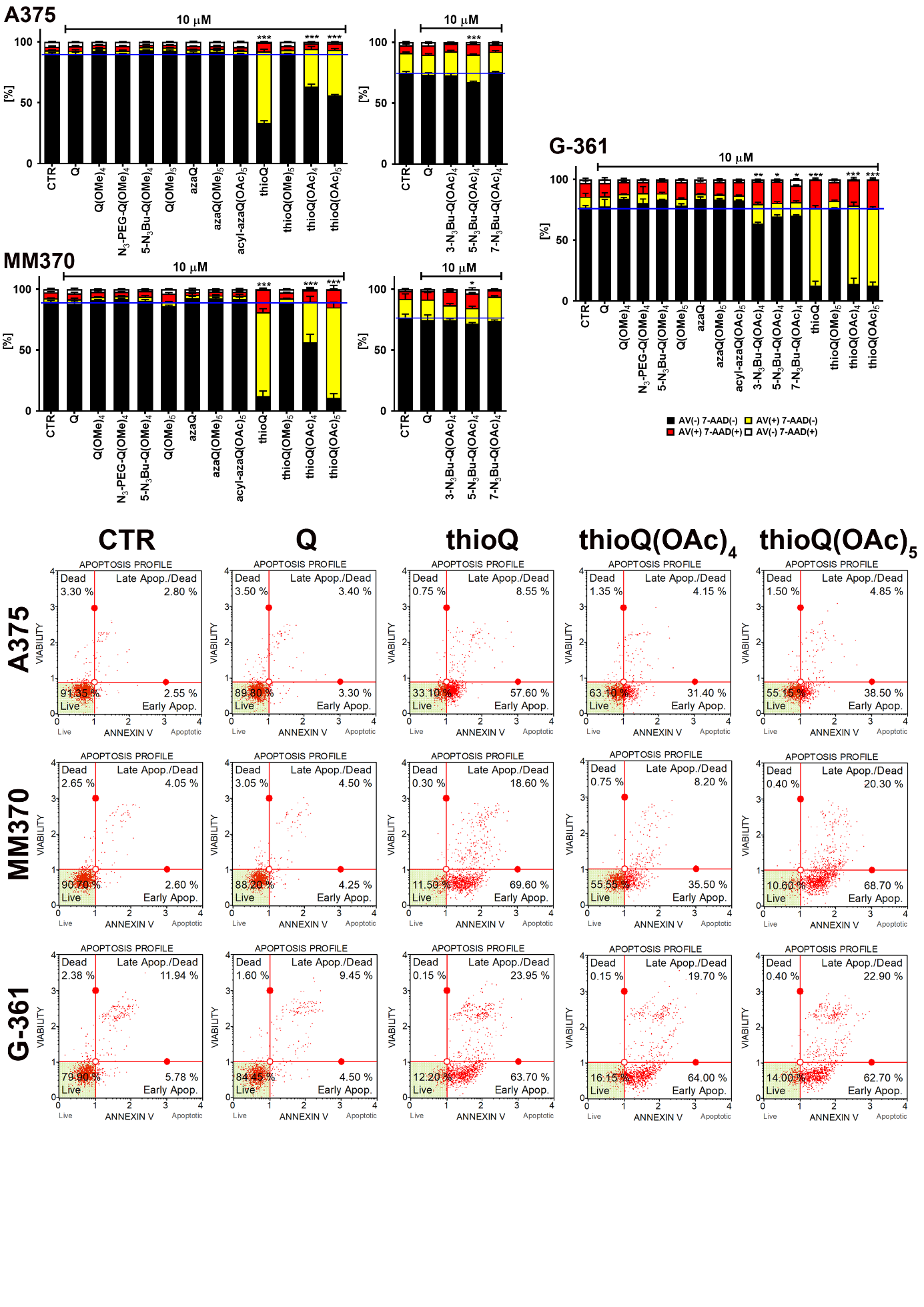
**

**Figure S46.** Quercetin derivative-mediated apoptotic cell death in three melanoma cell lines, namely A375, MM370, and G-361. Cells were treated with quercetin and fourteen quercetin derivatives (10 µM for 24 h). Apoptosis and necrosis were evaluated using dual staining based on Annexin V staining and 7-AAD staining, and flow cytometry. Bars indicate SD, *n*=3, ^***^*p* < 0.001, ^**^*p* < 0.01, ^*^*p* < 0.05 compared to control (CTR) (ANOVA and Dunnett’s a posteriori test). A blue horizontal line is used to emphasize the action of quercetin derivatives compared to CTR. Representative dot-plots are also shown. CTR, control conditions; Q, quercetin treatment; 17D, treatment with a HSP90 inhibitor alvespimycin (17-DMAG).


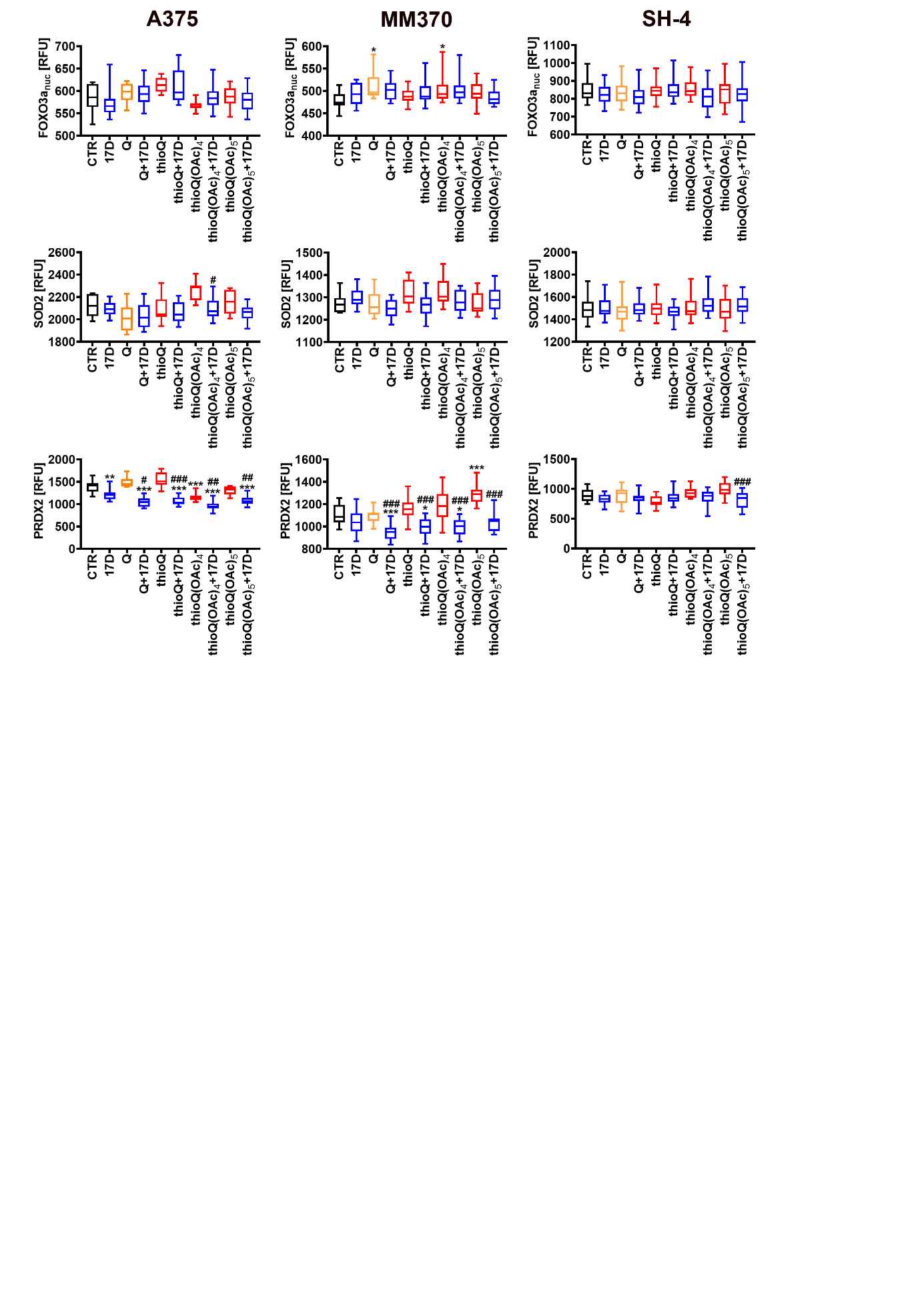


**Figure S47.** Thioquercetin-associated adaptive response to oxidative stress induction in three melanoma cell lines (A375, MM370, and SH-4 cells). Synergistic action of HSP90 inhibition was also considered. Cells were co-treated with 5 µM quercetin or thioquercetins (thioQ, thioQ(OAc)_4_, and thioQ(OAc)_5_) and 100 nM 17-DMAG, a HSP90 inhibitor, for 24 h. The activation of a regulator of antioxidant adaptive response, namely FOXO3a transcription factor and the levels of selected antioxidant enzymes SOD2 and PRDX2 were studied using dedicated antibodies, immunofluorescence protocol, and imaging cytometry. The nuclear levels of FOXO3a and total levels of SOD2 and PRDX2 are presented as relative fluorescence units (RFU). Box and whisker plots are shown, *n* = 3, ^***^*p* < 0.001, ^**^*p* < 0.01, ^*^*p* < 0.05 compared to control (CTR) (ANOVA and Dunnett’s a posteriori test), ^###^*p* < 0.001, ^##^*p* < 0.01, ^#^*p* < 0.05 compared to the treatment with quercetin or corresponding thioquercetin derivative (ANOVA and Tukey’s a posteriori test). CTR, control conditions; Q, quercetin treatment; 17D, treatment with a HSP90 inhibitor alvespimycin (17-DMAG).


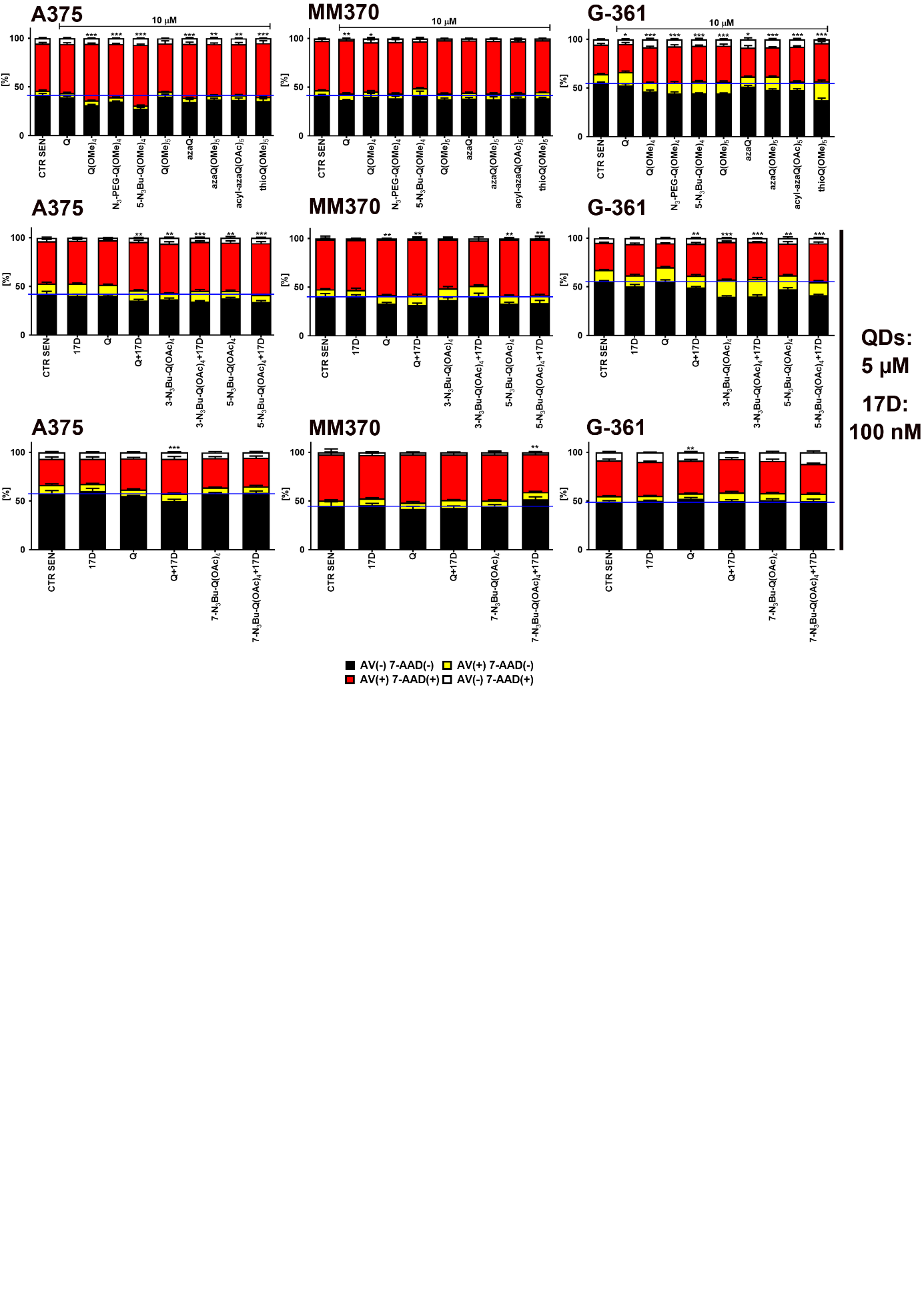


**Figure S48.** Quercetin derivative-mediated senolytic activity in drug-induced senescent melanoma cells. Synergistic action of HSP90 inhibition was also considered in selected experimental settings. To activate drug-induced senescence program, melanoma cells were treated with cisplatin (2-4 µM depending on cell line used) for 24 h, and then the drug was removed, and cells were cultured for additional 7 days for the development of cisplatin-induced senescence phenotype. Senescent cells were then co-treated with 5 or 10 µM quercetin or quercetin derivatives (QDs) and 100 nM 17-DMAG, a HSP90 inhibitor, for 24 h to analyze senolytic activity of tested compounds. Apoptosis and necrosis were evaluated using dual staining based on Annexin V staining and 7-AAD staining, and flow cytometry. Bars indicate SD, *n*=3, ^***^*p* < 0.001, ^**^*p* < 0.01, ^*^*p* < 0.05 compared to senescence control (CTR SEN) (ANOVA and Dunnett’s a posteriori test). A blue horizontal line is used to emphasize the action of quercetin derivatives and HSP90 inhibitor compared to CTR SEN. Representative dot-plots are also shown. CTR SEN, control senescence conditions; Q, quercetin treatment; 17D, treatment with a HSP90 inhibitor alvespimycin (17-DMAG).

**References**

1. Ravishankar, D., K.A. Watson, F. Greco and H.M.I. Osborn, *Novel synthesised flavone derivatives provide significant insight into the structural features required for enhanced anti-proliferative activity.* RSC Advances, 2016. **6**(69): p. 64544-64556. 10.1039/C6RA11041J.

2. de la Torre, M.D.L., A.C. Tomé, A.M.S. Silva and J.A.S. Cavaleiro, *Synthesis of [60]fullerene–quercetin dyads.* Tetrahedron Letters, 2002. **43**(26): p. 4617-4620. <https://doi.org/10.1016/S0040-4039(02)00867-5>.

3. Piotrowski, P., J. Pawłowska, J. Pawłowski, A. Więckowska, R. Bilewicz*, et al.*, *Nanostructured films of in situ deprotected thioacetyl-functionalized C60-fullerenes on a gold surface.* Journal of Materials Chemistry A, 2014. **2**(7): p. 2353-2362. 10.1039/C3TA13844E.

4. D'Hooghe, M., Z. Szakonyi, F. Fülöp and N.D. Kimpe, *Synthesis of n-(4-chlorobutyl)butanamide, a chlorinated amide isolated from aloe sabaea.* Organic Preparations and Procedures International, 2003. **35**(5): p. 501-507. 10.1080/00304940309355861.

5. Przybylski, P., A. Lewińska, I. Rzeszutek, D. Błoniarz, A. Moskal*, et al.*, *Mutation Status and Glucose Availability Affect the Response to Mitochondria-Targeted Quercetin Derivative in Breast Cancer Cells.* Cancers, 2023. **15**(23). 10.3390/cancers15235614.

6. Mattarei, A., L. Biasutto, E. Marotta, U. De Marchi, N. Sassi*, et al.*, *A Mitochondriotropic Derivative of Quercetin: A Strategy to Increase the Effectiveness of Polyphenols.* ChemBioChem, 2008. **9**(16): p. 2633-2642. <https://doi.org/10.1002/cbic.200800162>.

7. Sui, Z., V.N. Nguyen, J. Altom, J. Fernandez, J.J. Hilliard*, et al.*, *Synthesis and topoisomerase inhibitory activities of novel aza-analogues of flavones.* European Journal of Medicinal Chemistry, 1999. **34**(5): p. 381-387. <https://doi.org/10.1016/S0223-5234(99)80087-7>.

8. Zima, V., K. Radilová, M. Kožíšek, C.B. Albiñana, E. Karlukova*, et al.*, *Unraveling the anti-influenza effect of flavonoids: Experimental validation of luteolin and its congeners as potent influenza endonuclease inhibitors.* European Journal of Medicinal Chemistry, 2020. **208**: p. 112754. <https://doi.org/10.1016/j.ejmech.2020.112754>.
